# Transcription co-activator P300 activates Elk1-aPKC-ι signaling mediated epithelial-to-mesenchymal transition and malignancy in hepatocellular carcinoma

**DOI:** 10.1038/s41389-020-0212-5

**Published:** 2020-03-06

**Authors:** Chaoqun Ma, Shuhong Huang, Lei Xu, Li Tian, Yan Yang, Jianming Wang

**Affiliations:** 1 Department of Biliary and Pancreatic Surgery/Cancer Research Center, Affiliated Tongji Hospital, Tongji Medical College, Huazhong University of Science and Technology, 430030 Wuhan, China; 2 Institute of Basic Medicine, Shandong Academy of Medical Sciences, 250000 Jinan, China; 3 Department of Hepatopancreatobiliary Surgery, The First Affiliated Hospital, Hainan Medical University, 570102 Haikou, China; 4 Department of Hepatobiliary Pancreatic Surgery, Affiliated Tianyou Hospital, Wuhan University of Science & Technology, 430064 Wuhan, China

**Keywords:** Liver cancer, Oncogenes, Metastasis, Cell signalling, Cancer genetics

## Abstract

Epithelial-to-mesenchymal transition (EMT) plays an important role in invasion and metastasis of hepatocellular carcinoma (HCC). Our previous study found that atypical protein kinase C-ι (aPKC-ι) promoted the EMT process in HCC. However, how the aPKC-ι signaling pathway is regulated in HCC has not been elucidated. In this study, vector transfection was utilized to study the invasion of HCC cells, and the mechanism between P300 and aPKC-ι signaling pathways in regulating the EMT process of HCC was further elucidated in vitro and in vivo. We found both P300 and aPKC-ι were highly expressed in HCC and they were correlated with tumor progression and poor survival in HCC patients. P300 knockdown inhibited EMT, invasion and other malignant events of HCC cells but promoted cell apoptosis and cycle arrest. However, the effects mediated by P300 knockdown were abolished by aPKC-ι overexpression. Further studies showed that P300 upregulates aPKC-ι expression through increasing the transcription of Elk1, a transcriptional activator of aPKC-ι, and stabilizing Elk1 protein and its phosphorylation. In conclusion, our work uncovered the molecular mechanism by which oncogenic aPKC-ι is upregulated in HCC and suggests that P300, like aPKC-ι, may be used as a prognostic biomarker and therapeutic target in patients with HCC.

## Introduction

Hepatocellular carcinoma (HCC) is the sixth most common human malignancy and the second leading cause of cancer-related deaths world-wide^1,2^. The risk of 5-year recurrence of HCC following hepatic resection is as high as 50–70% due to its propensity for invasion and frequent development of intra-hepatic and/or extra-hepatic metastases^3,4^. Epithelial–mesenchymal transition (EMT) has been shown to be a pivotal mechanism contributing to cancer invasion and metastasis, as epithelial cells lose their polarity and acquire the migratory properties of mesenchymal cells. Although several EMT-related transcription factors such as Snail, Twist, and zinc finger E-box binding protein 1 (ZEB1) have demonstrated involvement in the process of EMT in HCC^5,6^, the molecular mechanism underlying the regulation of EMT in HCC has not been fully elucidated.

Atypical protein kinase C-ι (aPKC-ι) is a aPKC, which is regarded as a human oncogene and a potential therapeutic target in various human tumors, including HCC^7^. We previously reported that the expression of aPKC-ι was increased in HCC tumors when compared with peri-tumoral and normal liver tissues^8,9^. Accumulation of aPKC-ι in the cytoplasm and nucleus of HCC tissues was associated with the loss of polarity and tight junctions, as well as a significant decrease in E-cadherin and accumulation of cytoplasmic β-catenin in HCC^8^. We further found that the aPKC-ι-Par6 signaling pathway promoted the EMT phenotype and led to increased invasion and metastasis of HCC, as well as apoptosis resistance in an EMT cell model of HCC^10^. We also demonstrated that aurothiomalate (ATM), a drug being tested in phase I clinical trials in patients with lung cancer, exerted antitumor effects and could regulate the EMT process by inhibiting aPKC-ι-Par6 signaling^10^. Although our previous studies have provided evidence for the involvement of the aPKC-ι-Par6 signaling pathway in promoting EMT via the TGF-β1 and Ras signaling axis, our understanding of the mechanisms regulating aPKC-ι expression in HCC tumors is less well characterized.

P300 was originally discovered as a transcriptional co-activator that plays pivotal roles in integrating and mediating multiple signal-dependant transcription events^11^. The most studied function of P300 is as a histone acetyltransferase, modulating transcription via chromatin remodeling^12^. However, the effect of P300 as a transcriptional regulator of EMT remains poorly understood. Recently, it has been reported that high P300 expression in HCC tissues predicts a poor prognosis in association with EMT of HCC cells^13,14^. In the current study, we investigated the expression and relationship between P300 and aPKC-ι in HCC tissues with an attempt to identify the relationship between the occurrence, development, invasion and prognosis of HCC. In addition, our efforts were focused on clarifying the interaction between P300 and the aPKC-ι signaling pathways in regulating the EMT process of HCC in vitro and in vivo.

## Results

### Concomitant overexpression of P300 and aPKC-ι correlates with HCC progression and a poor prognosis

**Fig. 1:**
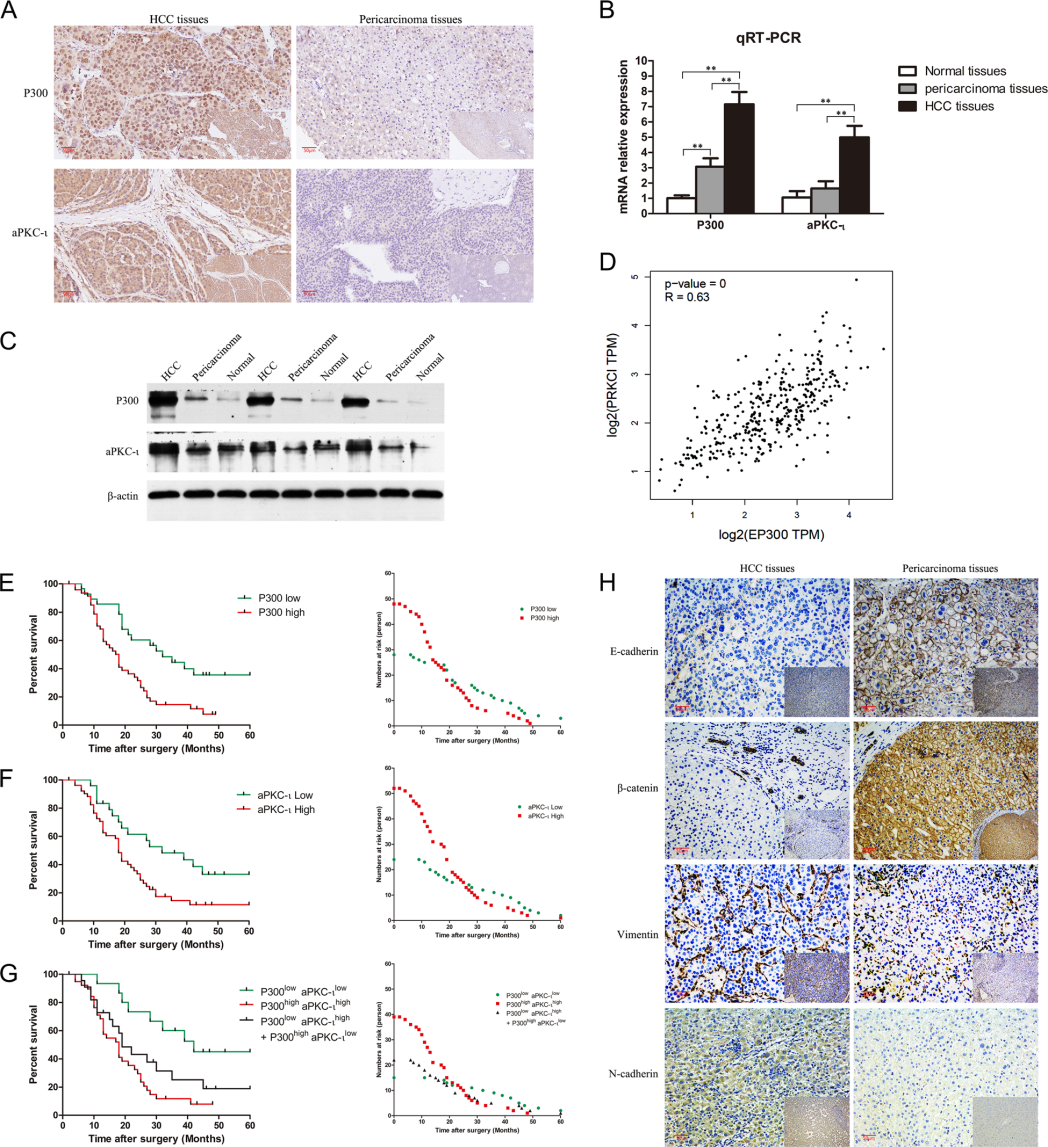
Concurrent overexpression of P300 and aPKC-ι promotes tumor progression and correlates with a poor prognosis in HCC patients. **a** Representative IHC staining of the expression of P300 and aPKC-ι protein in HCC tissue (left panels) and pericarcinoma tissues (right panels) were shown (×20). Scale bar, 50 μm. **b** qRT-PCR was used to measure the expression of HCC, pericarcinoma and normal tissues. Total RNA was isolated from at least three samples of each tissue and *ACTB* was used as the internal control. Fold changes were calculated through relative quantification (2^−ΔΔCt^). (*n* = 3, Student’s *t*-test, mean ± SD, **P < 0.01). **c** Expression of P300 and aPKC-ι in HCC, pericarcinoma and normal tissues were detected by WB analysis and β-actin was used as the internal control (Three different patients). **d** Correlation analysis of aPKC-ι (PRCKI) and P300 (EP300) gene expression in HCC tissues in the TCGA database. There was a positive correlation between the expression of aPKC-ι and P300 in HCC tissues (*R* = 0.63, *P* < 0.001). **e** Kaplan–Meier analysis. Patients in the low P300 group (*n* = 28) experienced a significantly longer overall survival (OS) than patients in the high P300 group (*n* = 48) (median OS: 32 months vs 18 months, *P* = 0.0012, log-rank test; left panel). The numbers of risk were shown in the right panel. **f** Kaplan–Meier analysis. Patients in the low aPKC-ι group (*n* = 24) experienced a significantly longer OS than patients in the high aPKC-ι group (*n* = 52) (median OS: 32 months vs 18 months, *P* = 0.0085, log-rank test; left panel). The numbers of risk were shown in the right panel. **g** Kaplan–Meier analysis. Patients in the P300^low^aPKC-ι^low^ group (*n* = 15) experienced a significantly longer OS than patients in the P300^high^aPKC-ι^high^ group (*n* = 39) as well as the group with overexpression of only P300 or aPKC-ι (P300^high^aPKC-ι^low^ + P300^low^aPKC-ι^high^) (*n* = 22) (median OS: 42 months vs 18 months *P* = 0.0005, log-rank test; median OS: 42 months vs 19 months *P* = 0.0474, log-rank test, respective; left panel). The numbers of risk were shown in the right panel. **h** Representative IHC staining of the expression of E-cadherin, β-catenin, Vimentin, and N-cadherin protein in HCC tissue (left panels) and pericarcinoma tissues (right panels) were shown (×20). Scale bar, 50 μm.

We examined the protein levels of P300 and aPKC-ι in 76 paired HCC and pericarcinoma tissue samples using Immunohistochemistry (IHC). As shown in Fig. 1a, P300 is predominantly localized in the nucleus, while aPKC-ι is distributed diffusely in both cytoplasm and nucleus. This is consistent with previous reports^8,13^. Interestingly, the levels of both P300 and aPKC-ι levels in HCC tumor cells were much higher than those in the pericarcinoma tissues. Additionally, we confirmed that the mRNA and protein levels of P300 and aPKC-ι in HCC tumors were significantly higher compared to those in pericarcinoma and normal tissues using qRT-PCR assay and WB, respectively (Fig. 1b, c). According to histopathologic evaluation, overexpression of P300 (score > 4) was detected in 63.16% (48/76) of HCC tissues vs. 32.89% (25/76) of pericarcinoma tissues (*χ*^2^ = 13.943, *P* < 0.001; Supplementary Table 1). Similarly, overexpression of aPKC-ι (score > 4) was detected in 68.42% (52/76) of HCC tissues vs. 48.68% (37/76) of pericarcinoma tissues (*χ*^2^ = 6.100, *P* = 0.014; Supplementary Table 1). Notably, overexpression of P300 in HCC tumors was related to AFP ≥20 ng/ml (*χ*^2^ = 8.586; *P* = 0.003), TNM stage III–IV (*χ*^2^ = 6.955; *P* = 0.008) and medium/poor differentiation (*χ*^2^ = 16.984; *P* < 0.001), while overexpression of aPKC-ι was related to tumor size >5 cm (*χ*^2^ = 14.226; *P* < 0.001), AFP ≥20 ng/ml (*χ*^2^ = 10.283, *P* = 0.001), vascular invasion (*χ*^2^ = 4.743, *P* = 0.029), TNM stage III-IV (*χ*^2^ = 6.610; *P* = 0.010) and medium/poor differentiation (*χ*^2^ = 14.032, *P* < 0.001; Supplementary Tables 1 and 2). The analysis of TCGA database indicated that the expression of P300 and aPKC-ι in HCC tissues was positively correlated (*R* = 0.63, *P* < 0.001, Fig. 1d). Moreover, overexpression of P300 and aPKC-ι were both negatively correlated with patient survival (Fig. 1e, f, Supplementary Table 3). Meanwhile, P300^high^aPKC-ι^high^ HCC patients and patients with overexpression of only P300 or aPKC-ι (P300^high^aPKC-ι^low^ + P300^low^aPKC-ι^high^) had a much shorter overall survival (OS) than that of P300^low^aPKC-ι^low^ HCC patients (Fig. 1g, Supplementary Table 3). Furthermore, both univariate and multivariate COX regression analyses indicated that P300, like TNM stage, AFP and tumor size, was an independent factor affecting the survival of HCC patients (Supplementary Table 4, *P* = 0.043). By combining this information with the TCGA database, we determined that there was a positive correlation between aPKC-ι and P300 in HCC patients. These data indicate that P300 and aPKC-ι interact to promote HCC progression and suggest that they may both act as potential prognostic indicators for predicting clinical outcomes for HCC patients.

### Knockdown of P300 or aPKC-ι inhibited EMT phenotype and invasion-associated events in HCC cells

In the current study, we examined the expression of EMT-associated markers, including E-cadherin, β-catenin, Vimentin and N-cadherin, in HCC tumors and pericarcinoma tissues (Fig. 1g). We found that the expression of E-cadherin and β-catenin in HCC tumors was significantly lower compared to pericarcinoma tissues; in contrast, the expression of Vimentin and N-cadherin in HCC tumors was significantly higher than in pericarcinoma tissues, which indicates a classic EMT phenotype of HCC tumors^10,15,16^. Since concomitant overexpression of P300 and aPKC-ι promoted the progression of HCC, we hypothesized that P300 and aPKC-ι may interact to enhance the EMT phenotype of HCC cells.

**Fig. 2:**
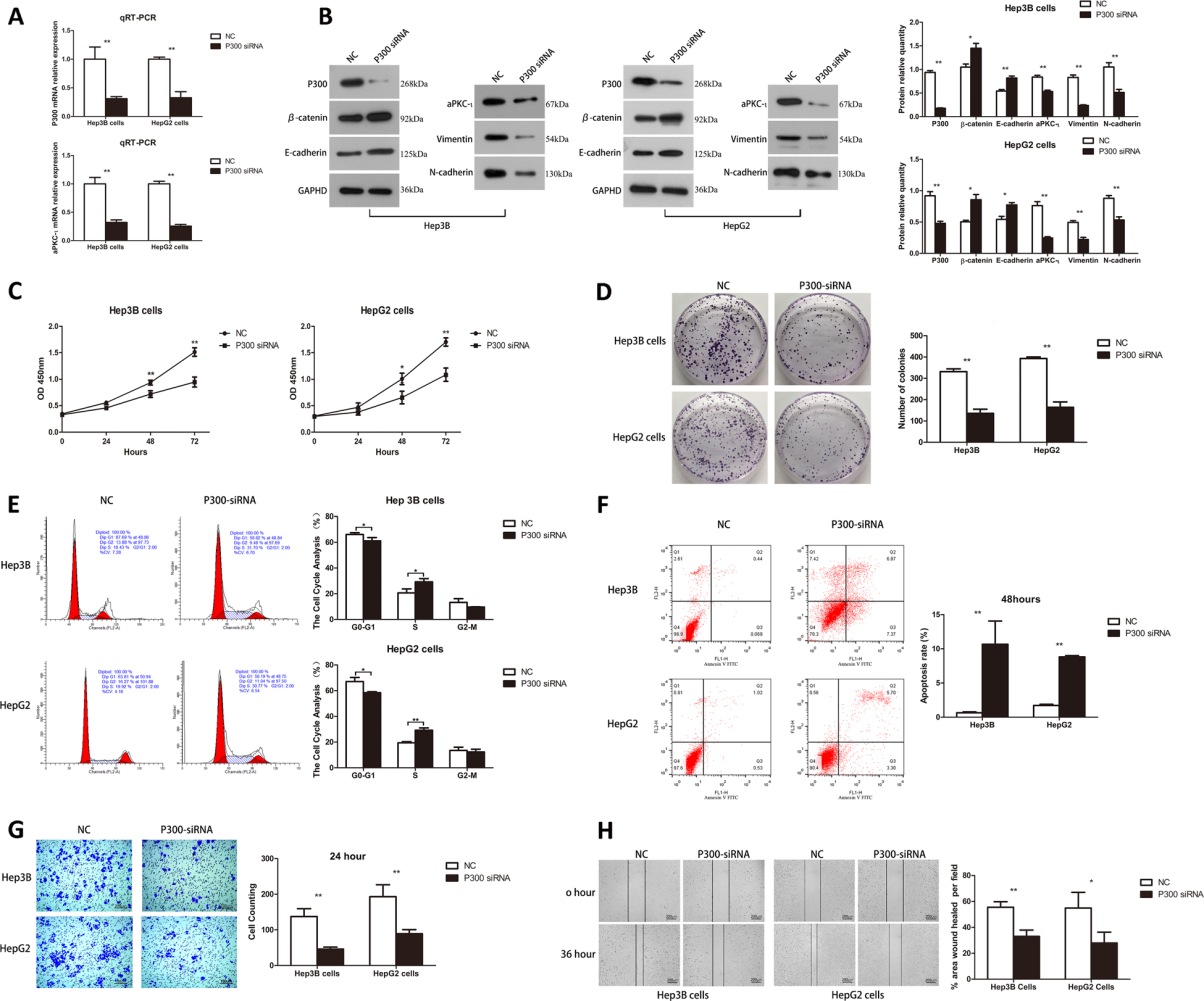
Knockdown of P300 reduced the expression of aPKC-ι, inhibited EMT, proliferation, invasion and migration, and promoted apoptosis and cell cycle arrest in HCC cell lines. **a** qRT-PCR was used to analyze mRNA expression of P300 (upper panel) or aPKC-ι (lower panel) in Hep3B and HepG2 cells (*n* = 3, Student’s *t-*test, mean ± SD, **P < 0.01). **b** WB was used to analyze the protein level of P300, aPKC-ι, and EMT markers (E-cadherin, β-catenin, N-cadherin and vimentin) in Hep3B (left panel) and HepG2 (middle panel) cells. GAPDH was used as the internal control. Statistical analysis of relative optical density of each band was shown (right panel; *n* = 3, Student’s *t*-test, mean ± SD, *P < 0.05, **P < 0.01). **c** CCK8 assay was used to investigate the proliferative kinetics of Hep3B (left panel) and HepG2 (right panel) cells in 72 h (*n* = 3, Student’s *t*-test, mean ± SD, *P < 0.05, **P < 0.01). **d** Colony formation assays were performed to evaluate the proliferative capability of Hep3B (upper panels) and HepG2 (lower panels) cells. A representative image was shown (left panels). Statistical comparison of the indicated groups was performed (right panel; *n* = 3, Student’s *t*-test, mean ± SD, **P < 0.01). **e** FACS analysis was used to investigate differences in cell cycle distribution following P300 silencing. Representative images were shown in the left panels (Hep3B upper, HepG2 lower). The results were statistically analyzed in the right panels (*n* = 3, Student’s *t*-test, mean ± SD, *P < 0.05, **P < 0.01). **f** Apoptosis of Hep3B (upper panels) and HepG2 (lower panels) cells was determined by flow cytometry. Representative images were shown in the left panels. The results were statistically analyzed in the right panel (*n* = 3, Student’s *t*-test, mean ± SD, **P < 0.01). **g** Transwell invasion assay was used to evaluate the invasion ability of Hep3B (upper panels) and HepG2 (lower panels). Representative images were shown in the left panels (×40). Scale bar, 100 μm. The results are statistically analyzed in the right panel (*n* = 3, Student’s *t*-test, mean ± SD, **P < 0.01). **h** Wound healing assay was performed to measure the migration ability of Hep3B and HepG2. Representative images were shown in the left panels (×4). Scale bar, 200 μm. The results were statistically analyzed in the right panel (*n* = 3, Student’s *t*-test, mean ± SD, *P < 0.05, **P < 0.01). For all experiments, cells transfected with P300 siRNA lentivirus were used as the experimental group, while cells transfected with empty vector were used as the negative control (NC).

To study the role of P300 in HCC proliferation and EMT phenotype, we stably knocked down P300 in Hep3B and HepG2 cells using lentivirus containing P300-shRNA. qRT-PCR and WB were utilized for confirmation (Fig. 2a, b). Interestingly, the mRNA and protein levels of aPKC-ι were also significantly decreased when P300 was knocked down (Fig. 2a, b), indicating the dependence of aPKC-ι expression on P300. Along with P300 knockdown, the expression of epithelial markers E-cadherin and β-catenin were significantly increased, while the expression of mesenchymal markers Vimentin and N-cadherin were significantly reduced, suggesting the correlation between P300 expression and EMT phenotype. Next, we examined the growth and proliferation of HCC cells after P300 knockdown. We found that P300 knockdown significantly decreased the proliferation and colony formation of Hep3B and HepG2 cells (Fig. 2c, d). Cell cycle analysis revealed that P300 knockdown resulted in a decrease in cell cycle activity by blocking cells in the S phase (Fig. 2e). Furthermore, P300 knockdown increased cell apoptosis in both Hep3B and HepG2 cells (Fig. 2f). Taken together, these data demonstrated that P300 loss significantly decreased the viability and proliferation of HCC cells in vitro. We further examined the effect of P300 loss on cell invasion and migration. It was shown that P300 knockdown decreased cell invasion and migration in both Hep3B and HepG2 cells (Fig. 2g, h), which was consistent with the reduced EMT phenotype induced by P300 knockdown.

We also stably knocked down aPKC-ι in Hep3B and HepG2 cells and confirmed that aPKC-ι deficiency resulted in downregulation of mesenchymal markers (Vimentin and N-cadherin) and upregulation of epithelial markers (E-cadherin and β-catenin), which indicated a suppressed EMT phenotype (Supplementary Fig. 1A). Interestingly, aPKC-ι knockdown also notably decreased the protein and mRNA expression of P300, suggesting a positive feedback loop between aPKC-ι and P300 pathways (Supplementary Fig. 1A and B). Moreover, aPKC-ι loss exhibited a similarly suppressive effect as P300 knockdown on the proliferation and colony formation of HCC cells by inducing cell cycle arrest at S phase and initiating cell apoptosis (Supplementary Fig. 1C–F). Consistent with the role of aPKC-ι in promoting the EMT phenotype^10^, aPKC-ι loss also inhibited the invasion and migration of HCC cells in vitro (Supplementary Fig. 1G and H). Taking these results together, we determined that the level of P300 is positively correlated with the expression of aPKC-ι; knockdown of either P300 or aPKC-ι impaired the EMT phenotype, viability, proliferation and invasion of HCC cells.

### P300 promotes the EMT and invasion of HCC cells through upregulation of the aPKC-ι signaling pathway

**Fig. 3:**
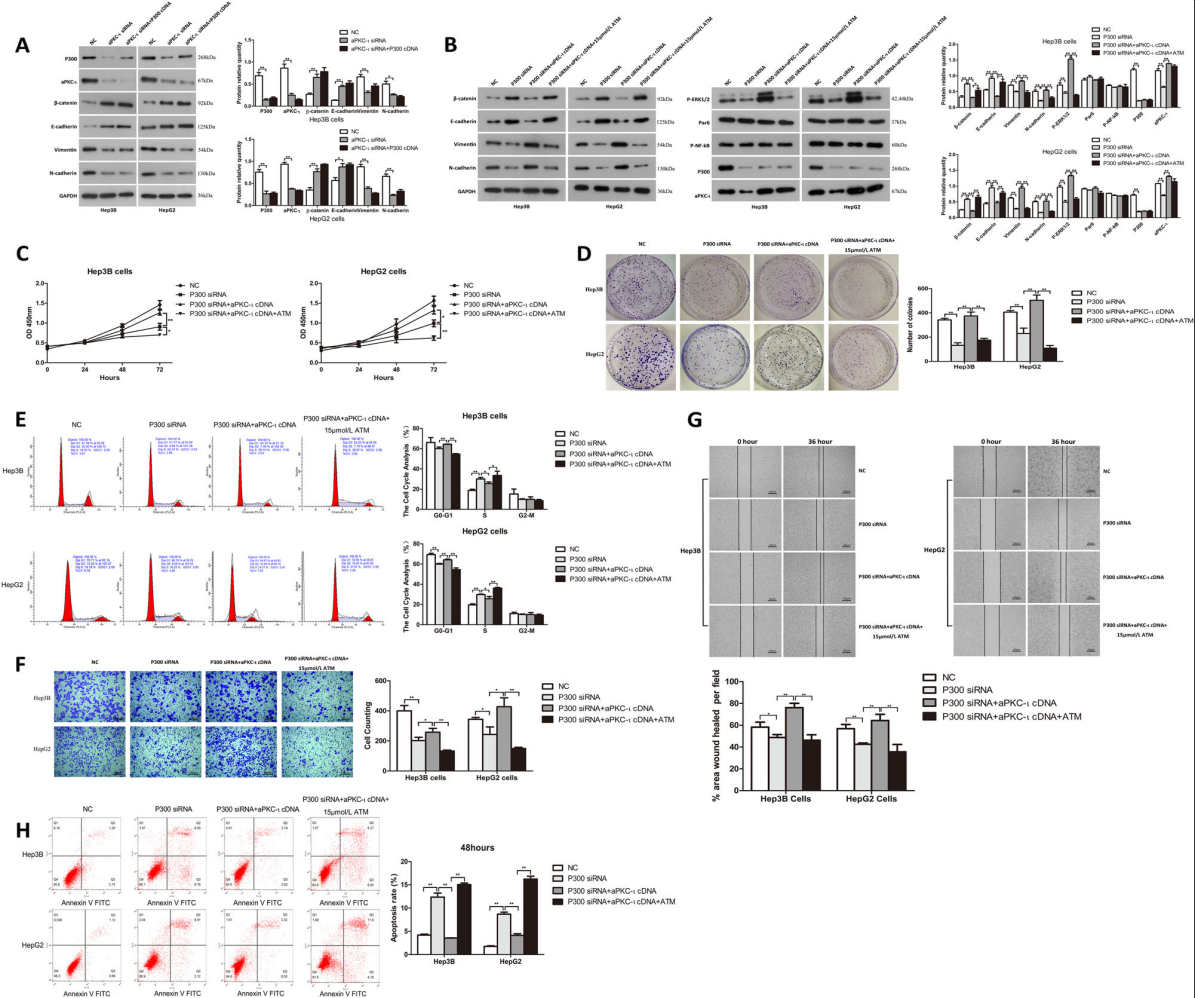
P300-knockdown-mediated phenotype was reversed by aPKC-ι rescue and further abolished by ATM treatment. **a** WB was used to analyze the protein level of P300, aPKC-ι and EMT markers (E-cadherin, β-catenin, N-cadherin and vimentin) in Hep3B and HepG2 cells (left panels). GAPDH was used as the internal control. Statistical analysis of relative optical density of each band was shown (right panel; *n* = 3, Student’s *t*-test, mean ± SD, *P < 0.05, **P < 0.01). **b** WB was used to analyze the protein level of P300, aPKC-ι, phospho-Erk1/2, Par6, phospho-NF-kB, and EMT markers (E-cadherin, β-catenin, N-cadherin and vimentin) in Hep3B and HepG2 cells (left and middle panels). GAPDH was used as the internal control. Statistical analysis of relative optical density of each band was shown (right panel; *n* = 3, Student’s *t*-test, mean ± SD, *P < 0.05, **P < 0.01). **c** CCK8 assay was used to investigate the proliferative kinetics of Hep3B (left panel) and HepG2 (right panel) cells (*n* = 3, Student’s *t*-test, mean ± SD, *P < 0.05, **P < 0.01). **d** Colony formation assays were performed to evaluate the proliferative capability of Hep3B and HepG2 cells. Representative images were shown (left panels). Statistical comparison of the indicated groups was performed (right panel). (*n* = 3, Student’s *t*-test, mean ± SD, **P < 0.01). **e** FACS analysis was used to investigate differences in cell cycle distribution in Hep3B and HepG2 cells. Representative images were shown in the left panels. The results were statistically analyzed in the right panels. (*n* = 3, Student’s *t*-test, mean ± SD, *P < 0.05, **P < 0.01). **f** Apoptosis of Hep3B (upper panels) and HepG2 (lower panels) cells was determined by flow cytometry. Representative images were shown in the left panels. The results were statistically analyzed in the right panel (*n* = 3, Student’s *t*-test, mean ± SD, **P < 0.01). **g** Transwell invasion assay was used to evaluate the invasion ability of Hep3B (upper panels) and HepG2 (lower panels). Representative images were shown in the left panels (×40). Scale bar, 100 μm. The results are statistically analyzed in the right panel (*n* = 3, Student’s *t*-test, mean ± SD, *P < 0.05, **P < 0.01). **h** Wound healing assay was performed to measure the migration ability of Hep3B and HepG2. Representative images were shown in the upper panels (×4). Scale bar, 200 μm. The results were statistically analyzed in the lower panel (*n* = 3, Student’s *t*-test, mean ± SD, *P < 0.05, **P < 0.01). For all experiments, cells transfected with empty vector were used as the negative control (NC).

Since aPKC-ι expression is positively correlated with the level of P300, a well-known transcriptional coactivator, we hypothesized that P300 promotes HCC progression, EMT and metastasis through upregulating aPKC-ι expression in HCC cells. To test this hypothesis, we restored the expression of P300 in aPKC-ι-knockdown Hep3B and HepG2 cells through transfection of recombinant plasmid carrying P300-cDNA. As shown in Fig. 3a, overexpression of P300-cDNA significantly increased the level of P300 in aPKC-ι-knockdown HCC cells; however, P300 rescue did not reverse the aPKC-ι knockdown-regulated expression pattern of EMT markers. These results suggest that aPKC-ι may be a downstream effector of P300 during invasion and metastasis of HCC. Next, we restored the expression of aPKC-ι in P300-knockdown Hep3B and HepG2 cells through transfection of recombinant plasmid carrying aPKC-ι-cDNA. As shown in Fig. 3b, overexpression of aPKC-ι-cDNA significantly increased the level of aPKC-ι in P300-knockdown HCC cells, and thus, the P300 knockdown-regulated expression pattern of EMT markers were all reversed by aPKC-ι rescue. Similarly, after aPKC-ι rescue, P300 knockdown-downregulated phosphorylated Erk1/2 (phospho-Erk1/2), which is the key downstream target of the aPKC-ι signaling pathway, were increased by aPKC-ι rescue^7^. However, the levels of the other two downstream targets (phospho-NF-kB and Par6) of the aPKC-ι signaling pathway were not significantly changed by aPKC-ι rescue. Furthermore, aPKC-ι overexpression rescued the cell proliferation, colony formation, cell cycle progression (via S phase arrest), invasion and migration of HCC cells (Fig. 3c–g), which were suppressed by P300 knockdown. Vise versa, aPKC-ι overexpression decreased HCC cell apoptosis (Fig. 3h), which was increased by P300 knockdown.

To further confirm that aPKC-ι is the downstream effector of P300, we treated the HCC cells with 15 μmol/L of ATM, an inhibitor of aPKC-ι, after P300 knockdown and aPKC-ι rescue^10^. We found that ATM abolished aPKC-ι overexpression-mediated rescue on HCC cell EMT markers, the activation of aPKC downstream targets (phospho-Erk1/2), proliferation and invasion-associated events as described above (Fig. 3). In conclusion, these data suggest that P300 promoted HCC EMT, proliferation and invasion through upregulation of the aPKC-ι pathway.

### P300 upregulates aPKC-ι through stabilizing transcription factor Elk1 and promoting its phosphorylation

**Fig. 4:**
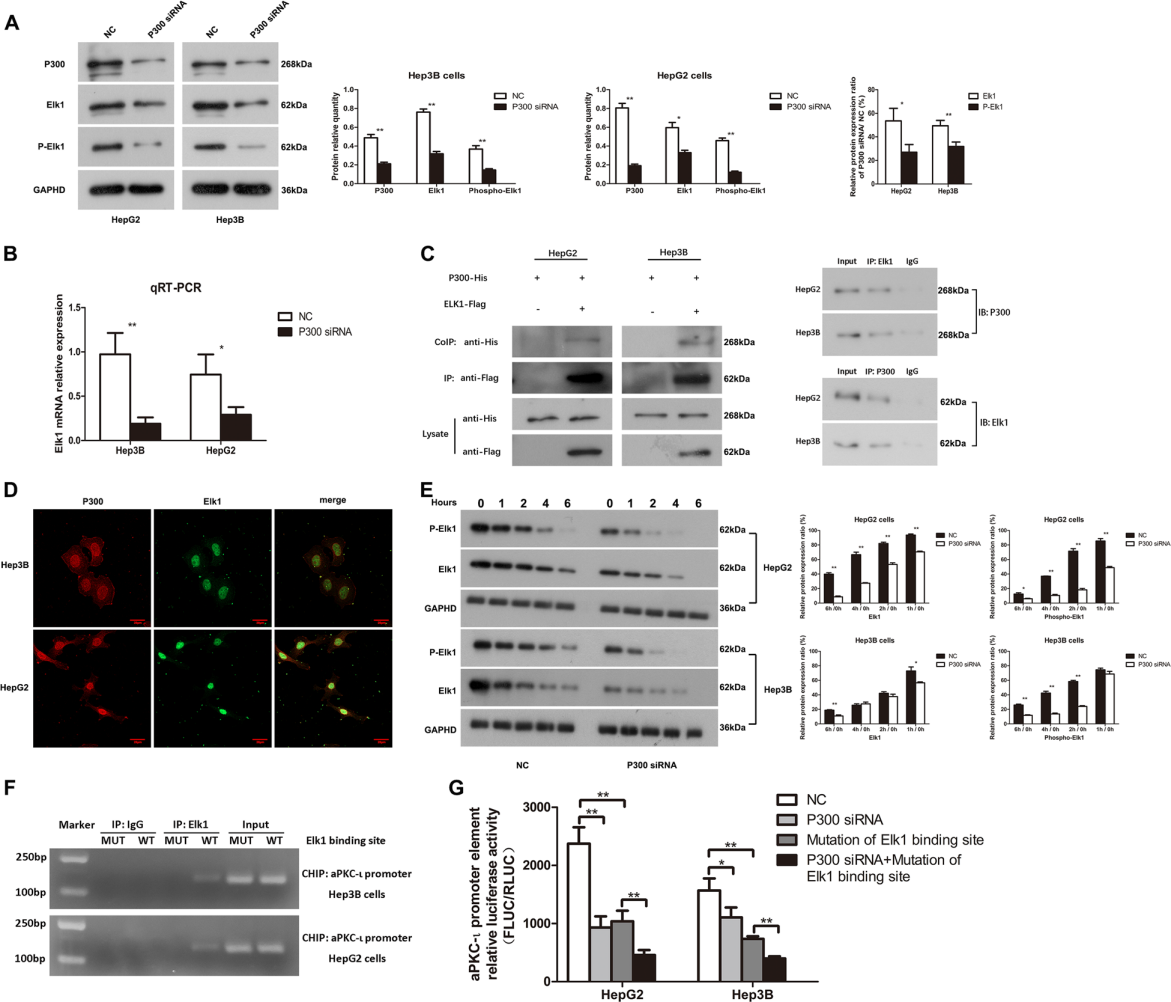
P300 promotes the transcription of aPKC-ι through upregulating Elk1 transcription and stabilizing its phosphorylation. **a** WB was used to analyze the protein level of P300, Elk1, and phospho-Elk1 in HepG2 and Hep3B cells after transfection with P300 siRNA lentivirus (left panel). GAPDH was used as the internal control. Statistical analysis of relative optical density of each band was shown (middle panel; *n* = 3, Student’s *t*-test, mean ± SD, *P < 0.05, **P < 0.01). The Elk1 and phospho-Elk1 relative expression ratio of P300 siRNA ^(Elk1/GAPHD; P-Elk1/GAPHD)^/NC ^(Elk1/GAPHD; P-Elk1/GAPHD)^ (%) was indicated in the right panel (*n* = 3, Student’s *t*-test, mean ± SD, *P < 0.05, **P < 0.01). **b** qRT-PCR was used to analyze mRNA expression of Elk1 in Hep3B and HepG2 cells (*n* = 3, Student’s *t*-test, mean ± SD, *P < 0.05, **P < 0.01). **c** The exogenous Co-IP was performed using lysates of HepG2 and Hep3B cells expressing either both His-tagged P300 and Flag-tagged Elk1 or only His-tagged P300 (left panel). Cell lysates were immunoprecipitated (IP) with anti-Flag antibody. The specific protein combination between P300 and Elk1 was determined by CO-IP with anti-His antibody. Cell lysates without immunoprecipitation underwent immunoblot analysis with indicated antibodies as the control. Specific protein-protein combinations between P300 and Elk1 in hepatoma cells were determined by endogenous Co-IP (right panel). The supernatants of the lysates without any antibody, named Input (10% total protein), were used as the positive control; proteins precipitated by IgG were used as the negative control. **d** Double immunofluorescent staining was used to evaluate the co-localization of P300 and Elk1 proteins in the hepatoma cells. The left panels were the representative picture of P300 protein (coraLite 594), the middle panels were the representative picture of Elk1 protein (CoraLite 488), and the right panels were the merged pictures (×40). Scale bar, 20 μm. **e** The rate of protein degradation of Elk1 and phospho-Elk1 in HepG2 and Hep3B cells was detected by WB. The cells were treated with 550 nM cycloheximide and then 5 time points (0, 1, 2, 4, 6 h) were selected for detection (left panel). GAPDH was used as the internal control. Statistical analysis of relative protein expression ratio was shown (right panel; *n* = 3, Student’s *t*-test, mean ± SD, *P < 0.05, **P < 0.01). **f** Specific protein-DNA combinations between Elk1 and aPKC-ι promoter in hepatoma cells were determined by Ch-IP. Hepatoma cells were transfected with recombinant plasmids to overexpress aPKC-ι promoter (WT group) or aPKC-ι promoter plasmids with a mutated Elk1 binding site (MUT group). Cell lysates without immunoprecipitation, named Input, were used as the positive control, and DNA precipitated by IgG were used as the negative control. **g** aPKC-ι promoter element relative luciferase activity (Fluc/Rluc) was detected by dual-luciferase reporter assay in Hep3B and HepG2 cells (*n* = 3, Student’s *t*-test, mean ± SD, *P < 0.05, **P < 0.01). Cells transfected with empty vector and a preserved ELK1 binding site vector were used as the negative control (NC).

Next, we studied the mechanism regarding P300 potentiation of aPKC-ι transcription. A previous study showed that the phosphorylation and activation of transcription factor Elk1 enhanced aPKC-ι transcription^17^. We hypothesized that P300 could function as a transcriptional co-activator to activate Elk1 and thus promote aPKC-ι transcription. To test this hypothesis, we first determined whether P300 could regulate Elk1 expression and phosphorylation in HCC cells. As shown in Fig. 4a, the levels of both total Elk1 and phosphorylated-Elk1 (phospho-Elk1) were decreased in P300-knockdown cells; the ratio of phospho-Elk1 to total Elk1 was also significantly decreased by P300-knockdown. Subsequently, we found that the mRNA level of Elk1 was significantly decreased in P300-knockdown HCC cells (Fig. 4b). These data suggested that P300 potentiated the transcription of Elk1 and thus increased its expression, and that P300 also promoted Elk1 phosphorylation in HCC cells.

To understand the interaction between P300 and Elk1, we performed the exogenous and endogenous co-immunoprecipitation (Co-IP) assay and revealed that P300 bound to Elk1 in HCC cells (Fig. 4c)^18^. Double immunofluorescent staining revealed the localization of P300 and Elk1 proteins were both in the nucleus of hepatoma cells (Fig. 4d). To further uncover the effect of P300 on the stability of total Elk1 protein and phospho-Elk1, we examined the protein level of Elk1 and its phosphorylation in both P300 knockdown and control cells using protein degradation experiments. Within 6 h after inhibition of protein synthesis using cycloheximide, the degradation of both total Elk1 and phospho-Elk1 levels was faster in P300 knockdown cells compared to control cells (Fig. 4e). These results indicated that P300 was beneficial to the stabilization of total Elk1 protein and its phosphorylation, which might be attributed to the binding between P300 and Elk1. To confirm the role of the P300/Elk1 pathway in promoting aPKC-ι transcription, we studied the specific protein-DNA combination between Elk1 and aPKC-ι promoter in hepatoma cells by Ch-IP assay (Fig. 4f). We found that aPKC-ι promoter can bind to Elk1 (WT group), but this binding effect disappeared when the Elk1 binding site of aPKC-ι promoter was deleted (MUT group). Furthermore, we studied the effects of P300-knockdown and mutated Elk1 binding site on aPKC-ι promoter activity using dual-luciferase reporter assay. We found that either P300 knockdown or mutation of the Elk1 binding site significantly impaired aPKC-ι promoter activity, and their combination further decreased aPKC-ι promoter activity (Fig. 4g). These data suggested that P300 promoted aPKC-ι promoter activity not only through upregulation of Elk1 transcription but also through stabilization of Elk1 protein and its phosphorylation.

### P300 knockdown deficiency of aPKC-ι signaling expression and repressed HCC tumor growth in vivo

**Fig. 5:**
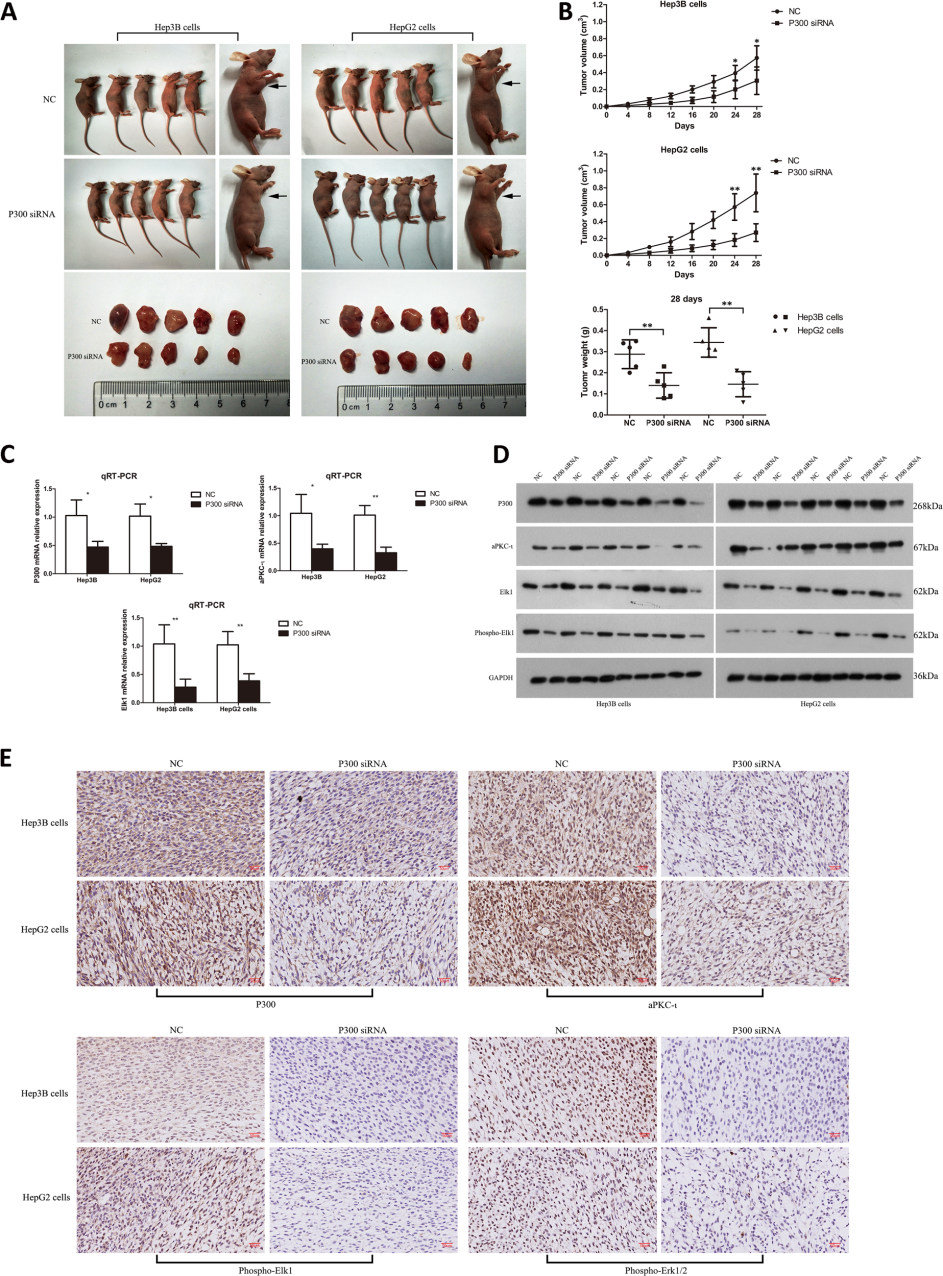
Knockdown of P300 downregulating the aPKC-ι signaling pathway and represses HCC tumorigenesis in vivo. **a** The effect of P300 silencing on tumor growth in vivo. Images of tumors formed in nude mice injected subcutaneously (arrows) with Hep3B (left panel) and HepG2 (right panel) cells. **b** Tumor growth curves show the volume of xenograft tumors for the Hep3B (upper panel) and HepG2 (middle panel) cells, and the final tumor weight was recorded and statistically analyzed (lower panel, *n* = 5, Student’s *t*-test, mean ± SD, *P < 0.05, **P < 0.01). **c** qRT-PCR was used to assess mRNA expression of P300 (upper left panel), aPKC-ι (upper right panel) and Elk1 (lower panel) in tumor xenograft tissues (*n* = 3, Student’s *t*-test, mean ± SD, *P < 0.05, **P < 0.01). **d** WB was used to assess the protein level of P300, aPKC-ι, Elk1 and phospho-Elk1 in tumor xenograft tissues. GAPDH was used as the internal control. **e** Representative images of IHC were used to detect the expression of P300 and aPKC-ι (upper panel), phospho-Elk1 and phospho-ERK1/2 (right panel) in tumor xenografts tissues of Hep3B and HepG2 cells (×40). Scale bar, 50 μm. For all experiments, cells transfected with P300 siRNA lentivirus were the experimental group, while cells transfected with empty vector were used as the negative control.

Since P300 is a significant contributor to the progression of HCC, we next studied the effect of P300 knockdown on HCC tumor growth in vivo using a subcutaneous xenograft tumor model. We found that P300 knockdown significantly suppressed the growth of both Hep3B and HepG2 tumors compared to the control (Fig. 5a, b). At day 28 after subcutaneous injection, there was a significant decrease in weight of the P300-knockdown tumors compared to the control tumors (Fig. 5b, lower panel). P300 loss and consequent deficiency of Elk1 and aPKC-ι in P300 knockdown tumors compared to control tumors were confirmed by qRT-PCR, WB and IHC (Fig. 5c–e). IHC further confirmed that P300-knockdown decreased phospho-Elk1 and downregulated expression of aPKC-ι downstream target phospho-Erk1/2 in HCC tumors (Fig. 5e). These data indicated that P300 promoted HCC tumor growth in vivo through upregulating the expression of aPKC-ι and its downstream pathway.

## Discussion

aPKC-ι is an oncogene and its signaling pathway is required for the maintenance of the transformed phenotype of cancer cells, promoting growth, survival and chemoresistance of several cancer types including HCC^7–9,19,20^. In this study, we further verified that aPKC-ι was highly expressed in HCC tissues accompanied with the classic EMT phenotype in HCC cells. Overexpression of aPKC-ι in HCC patients was correlated with a heavy tumor burden and poor prognosis^21^. Moreover, aPKC-ι loss significantly reduced the proliferation and colony formation of HCC cells by inducing cell cycle arrest and cell apoptosis. However, the mechanism of aPKC-ι pathway upregulation in HCC cells has not been elucidated. Interestingly, the expression level of transcriptional co-activator P300 is positively correlated with aPKC-ι, which indicates that P300 may be involved in the process of aPKC-ι signaling mediated malignant events.

The transcriptional co-activator P300 is involved in a variety of important cellular functions, such as cell proliferation, differentiation and apoptosis^22^. However, the role of P300 in EMT, invasion and metastasis of malignant tumors remains controversial. Some researchers have reported that P300, which is lowly expressed in malignant tumors, was a key factor that inhibited EMT and tumor growth. In studies on breast cancer, ovarian cancer and pancreatic cholangiocarcinoma, P300 is poorly expressed and played an important role in inhibiting EMT and sustaining E-cadherin expression in tumor cells through the regulation of P300-targeted miRNAs^23–26^. Nevertheless, some researchers have found that high expression of P300 in HCC predicted a poor prognosis in association with enhanced EMT of hepatocellular carcinoma^13,14^. In this study, we confirmed that P300 is highly expressed in HCC and is correlated with the EMT phenotype and poor prognosis of patients. We further proved that P300 knockdown resulted in low aPKC-ι expression, an inhibited EMT phenotype and decreased proliferation, migration and invasion of HCC cells, while also promoting apoptosis and cell cycle arrest in HCC cells. Silencing P300 in vivo had the same inhibitory effects on aPKC-ι expression and tumor growth. Our work revealed that the rescue experiment of aPKC-ι could reverse P300-knockdown-induced inhibition of EMT, proliferation, migration and invasion of HCC cells as well as cell cycle arrest and induction of cell apoptosis. Hence, P300 functions as a cancer promoter for HCC EMT and malignancy probably through upregulating the aPKC-ι signaling pathway. The opposite function of P300 in the context of different cancers might be related to different expression levels of aPKC-ι.

**Fig. 6:**
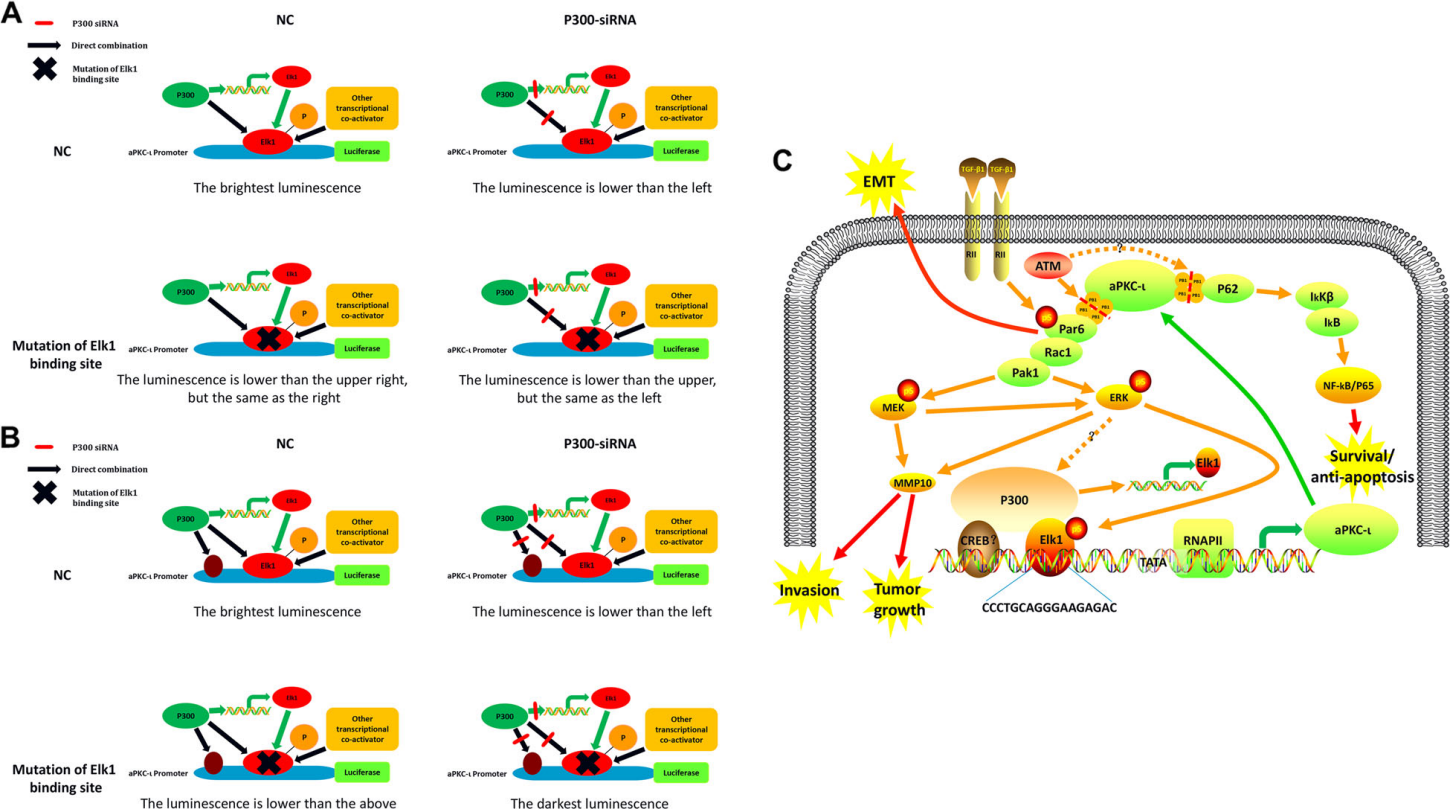
The hypothesis working model for the potential role of p300 in promoting the aPKC-ι signaling pathway. **a** Our hypothesis regarding the role of P300 in promoting aPKC-ι transcription is via binding to Elk1. In this hypothesis model, the luminescence levels of the Elk1-MUT and Elk1-MUT + P300-siRNA groups were identical, and lower than NC and P300-siRNA groups. Cells transfected with empty vector and a preserved ELK1 binding site vector were used as the negative control (NC). **b** The hypothesis model for the role of P300 in promoting aPKC-ι transcription via binding to Elk1 and other transcription factors. In this hypothesis model, the brightest luminescence level was in the NC group, and the darkest luminescence level was detected in the promoter Elk1-MUT + P300-siRNA group. Cells transfected with empty vector and a preserved ELK1 binding site vector were used as the negative control (NC). **c** The hypothesis regulatory network of the aPKC-ι signaling pathway in HCC: aPKC-ι-Par6-Rac1-Pak-Mek-Erk signaling axis and P300 jointly act on the Elk1 site of the aPKC-ι promoter to promote aPKC-ι transcription. TGF-β1 promotes EMT through the phosphorylation of Par6 and the involvement of aPKC-ι. aPKC-ι signals to downstream effectors such as Par6, Rac1, Mek, Erk, and NF-kB, which are important for tumor invasion, growth and anti-apoptosis. Arrows indicate the flow of signaling pathways; dotted arrows indicate potential flow of signaling pathways; the red dotted line indicates blocked binding; phosphorylation events are indicated by circled pS. See text for discussion and details.

P300 binds to multiple transcription factors as a co-activator to regulate gene transcription^27–29^. In this study, we confirmed that P300 promotes aPKC-ι transcription not only through upregulation of Elk1 transcription but also through stabilization of Elk1 protein and its phosphorylation. Therefore, we proposed two possible models representing the regulatory mechanism of aPKC-ι transcription by P300 and Elk1 (Fig. 6a, b). In the first model, shown in Fig. 6a, P300 only binds to Elk1 and regulates its activation. We proposed that P300 knockdown combined with the mutated Elk1 binding site would decrease aPKC-ι transcription to the same level as mutated Elk1 binding site alone, which is not consistent with our results. On the other hand, the second model is consistent with the results of our dual-luciferase assay, as it shows that P300 regulates aPKC-ι transcription through Elk1 and other possible transcription factors; hence, P300 knockdown combined with a mutated Elk1 binding site further reduces aPKC-ι transcription (Fig. 6b). P300 is closely related to CREB binding protein (CBP), and they share regions of very high sequence similarity including bromodomain, cysteine-histidine-rich regions and histone acetyltransferase domain^30^. In the model of CBP regulation by CD28 at the Fos gene, strong Erk-dependent phosphorylation of Elk-1 could stabilize the interaction between Elk-1 and CBP at the SRE of the Fos promoter^18^. CBP can be combined with CREB on the Fos gene promoter to regulate its transcriptional activity^18^, and our study found that P300 promotion of the aPKC-ι promoter transcriptional pathway is not unique. Herein, P300 perhaps interact with CREB on the aPKC-ι promoter simultaneously and synergize with Elk1 to activate the transcription of aPKC-ι (Fig. 6c).

In the study of NSCLC, it was found that aPKC-ι promoted EMT by increasing the phosphorylation of Par6 by type II TGF-β1 receptor. Additionally, the aPKC-ι-Par6-Rac1-Pak-Mek-Erk signaling axis drove anchorage-independent tumor growth and invasion through the induction of MMP10 expression^19,31–33^. Molecular dissection of signaling downstream of aPKC-ι demonstrated that the anti-rheumatoid agent ATM blocked the formation of the aPKC-ι-Par6 complex by specifically inhibiting PB1 domain-mediated interactions^34–37^. Our previous study confirmed this blocking effect and affect the function of hepatocellular EMT cells^10^. In this study, ATM efficiently inhibited aPKC-ι downstream effector phospho-Erk1/2, EMT markers, viability and invasion that were restored by aPKC-ι rescue in P300-knockdown HCC cells. Since there are no commercially available phospho-Par6 antibodies, the expression of Par6 was not significantly different with aPKC-ι rescue and ATM inhibition. However, the above results are enough to verify that the aPKC-ι-Par6-Rac1-Pak-Mek-Erk signaling axis may plays the same role in HCC cells (Fig. 6c). Furthermore, it was reported that the aPKC-ι-P62-IκKβ-IκB-NF-kB signaling axis induces anti-apoptotic genes and promotes cell survival^38,39^. Whether ATM can block the PB1 domain-mediated interaction between aPKC-ι and P62 is still controversial. The research on chronic myelogenous leukemia (CML) was positive, while the research on rheumatoid arthritis (RA) was negative^35,38,40^. In our study, aPKC-ι has no significant effect on NF-kB phosphorylation, but low ATM concentration inhibited aPKC-ι induced apoptosis of HCC cells, which suggested aPKC-ι may promote cell apoptosis via interference with the IκKβ-IκB-NF-kB complexes through P62 in HCC (Fig. 6c). In this study, we also found that aPKC-ι knockdown reduced the expression of P300, which may be due to downregulation of the aPKC-ι-Erk pathway^17,18^. This indicates that aPKC-ι gene expression may have a positive feedback loop to further amplify its expression (Fig. 6c).

In conclusion, our studies demonstrate that the aPKC-ι signaling pathway inducing the EMT phenotype and promoting cell proliferation, invasion and other malignant events in HCC, and P300 could boost these aPKC-ι-mediated biological functions through increasing aPKC-ι expression. Both P300 and aPKC-ι are independent factors for patient prognosis. We further uncovered that P300 upregulates aPKC-ι expression through increasing the transcription of Elk1, and by stabilizing Elk1 protein and its phosphorylation. Our work suggests that P300, like aPKC-ι, may be used as a prognostic biomarker and therapeutic target to inhibit aPKC-ι-induced EMT, invasion and metastasis in HCC patients. There are currently a variety of P300 blockers and the aPKC-ι signaling pathway blocker ATM has entered clinical research in the field of tumor therapy, which will provide a beneficial prospect for the targeted therapy of HCC in the future.

## Materials and methods

### Patients and clinicopathological data

A total of 76 consecutive patients with HCC during the time period of January 2012 to December 2016 underwent hepatectomies at the Department of Biliary and Pancreatic Surgery affiliated with Tongji Hospital, Huazhong University of Science and Technology, China. Freshly paired HCC and nontumor tissue samples (located 2 cm from tumor tissue) were collected. Ethical approval for this study was obtained from the Tongji Hospital Research Ethics Committee. The patients were not treated with any adjuvant therapy prior to surgery and the cancer was identified by two pathologists. Histopathologic diagnosis was based on the fourth edition of the World Health Organization criteria^41^ as well as the eighth edition of the AJCC Cancer Staging Manual^42^. Utilizing the preoperative Child-Pugh classification system, 75 patients were classified as grade A and 1 patient was classified as grade B. The clinicopathological characteristics of the 76 HCC patients are shown in Supplementary Table 2.

### Immunohistochemistry

Immunostaining (SABC method) and semi-quantitative analysis were performed to determine the expression of aPKC-ι, P300, E-cadherin, β-catenin, Vimentin and N-cadherin, as previously reported^21^. The IHC results were semi-quantitatively classified into five groups according to the percentage of positively stained cells and the signal intensity was evaluated as previously described ref. ^20^. A total score was determined by combining the two parameters. In this study, a total score ≤ 4 was regarded as low expression and >4 was regarded as high expression in the specimens. The IHC scores were assessed by three pathologists who were blind to patients’ conditions.

### Quantitative real-time polymerase chain reaction

Total RNA was extracted from tissue specimens or cells using TRIzol reagent (Life Technologies, CA, USA) according to the manufacturer’s instructions. Complementary DNA (cDNA) was synthesized using 2 μg of the total RNA according to the instructions of the reverse transcriptase kit (Takara Bio, Inc., Dalian, China) in a LifePro Thermal Cycler (Hangzhou Bioer Technology Co. Ltd., Hangzhou, China). qRT-PCR was performed using a SYBR® Premix EX Taq kit (Takara Bio, Inc., Dalian, China) for 40 cycles in a CFX Connect^TM^ Real-Time System (Bio-Rad, Hercules, CA, USA). Primers were designed and synthesized by Shanghai Sango Biotech Co. Ltd., Shanghai, China (listed in Supplementary File 1). The cycle threshold (Ct) of different genes was first normalized to β-actin for the same sample, and fold changes were calculated through relative quantification (2^−ΔΔCt^) as previously reported^21^. Each experiment was performed in triplicate.

### Cell lines and cell culture

Human hepatocellular carcinoma cell lines HepG2 and Hep3B (STR identification are shown in Supplementary Files 2 and 3; Institute of bioengineering, Chinese academy of sciences, China) with high P300 expression were selected based on preliminary studies. Cell lines were cultured in DMEM medium (Gibco, CA, USA) containing 10% fetal bovine serum (Gibco, CA, USA) and 1% penicillin-streptomycin solution (Beijing Solarbio Science & Technology Co., Beijing, China) at 37 °C in a 5% CO_2_ atmosphere. ATM (Sigma-Aldrich, St, Louis, USA) was dissolved using purified water and processed by sterile filtration. In all, 15 μmol/L of ATM was added to the medium when necessary; this concentration was determined based on previous studies^10^. In all, 550 nM of cycloheximide was added to the medium for the protein degradation experiment. The total time from data collection to completion of the cell experiment was one year.

### Lentiviral vector construction and establishment of stable cell clones

Two shRNAs of P300 (Genbank access number: NM_001429) and aPKC-ι (Genbank access number: NM_002740), which expressed P300-siRNA (5′-CAGAGCAGTCCTGGATTAG-3′)^43^ and aPKC-ι-siRNA (5′-TTTAGACTTTATGAGCTAA-3′)^21^, respectively, were synthesized by Shanghai Genechem Co., Ltd. (Shanghai, China). Three independent siRNAs per gene were used in the studies (listed in Supplementary File 1). The shRNAs were cloned into GV248 vector (hU6-MCS-Ubiquitin-EGFP-IRES-Puromycin) and GV112 vector (hU6-MCS-CMV-Puromycin) to construct siRNA-expressing lentivirus (listed in Supplementary File 1). The lentivirus with an empty vector was used as the negative control (NC). Lentivirus transduction was performed according to the GenePharma Recombinant Lentivirus Operation Manual (http://www.genepharma.com). Cells were transduced with 2 μl concentrated lentivirus (MOI = 10) in the presence of polybrebe (8 μg/ml) for 72 h. Then, cells were selected with 5 μg/ml puromycin (Sigma-Aldrich, St, Louis, USA) to generate stable cell lines. Knockdown of P300 and aPKC-ι was confirmed by quantitative real-time polymerase chain reaction (qRT-PCR) and western blot analysis (WB).

### Plasmid cloning and expression of recombinant proteins

The recombinant plasmid (PRKCI pcDNA3.1-3xFlag-C), which overexpresses aPKC-ι (listed in Supplementary File 1)^21^, was generated by Shanghai Genechem Co., Ltd. (Shanghai, China) and transfected into hepatoma cells for the aPKC-ι rescue experiment. The recombinant plasmid (pGL3-basic-pkci), which carries the aPKC-ι promoter sequence, and the pGL3-basic-pkci-elk1-kt plasmid (with deletion of the Elk1 binding site) were generated by Shanghai Generay Biotechnology Co., Ltd. (Shanghai, China) and transfected into hepatoma cells for the double luciferase assay (listed in Supplementary File 1). The Elk1-pcDNA3.1-3xFlag-C plasmid and pcDNA3.1-EP300-6xHis (listed in Supplementary File 1), which were used to overexpress Elk1-Flag and P300-His, respectively, were generated by Shanghai Genechem Co., Ltd. (Shanghai, China). Overexpression using these plasmids was verified by qRT-PCR and western blot analysis after transfection.

### Western blotting

The tissues and cells were lysed with RIPA lysis buffer in addition to 1 mM PMSF. The protein concentration was determined by Coomassie protein assay (Pierce, Rockford, IL, USA). Proteins were separated by SDS-polyacrylamide gel electrophoresis (SDS-PAGE; Bio-Rad, Hercules, CA, USA) and transferred onto a nitrocellulose membrane (Amersham, Chalfont, UK). The membranes were incubated with primary antibodies (aPKC-ι, abcam, Cambridge, UK; P300, abcam, Cambridge, UK; Elk1/phospho-Elk1, abcam, Cambridge, UK; E-cadherin, Proteintech, Rosemont, USA; β-catenin, Proteintech, Rosemont, USA; Vimentin, Proteintech, Rosemont, USA; N-cadherin, Proteintech, Rosemont, USA; Par6, abcam, Cambridge, UK; phospho-NF-kB, abcam, Cambridge, UK; phospho-Erk1/2, abcam, Cambridge, UK; Anti-His, Sigma-Aldrich, St, Louis, USA; Anti-Flag, Sigma-Aldrich, St, Louis, USA) at 4 °C overnight. GAPDH (Proteintech, Rosemont, USA) and β-actin (Proteintech, Rosemont, USA) were used as internal controls. Peroxidase-conjugated secondary antibodies were incubated at room temperature for 60 min. Peroxidase activity was detected using the chemiluminescence method (Amersham) and visualized on X-Omat S films (Amersham). The assays were repeated three times.

### Cell viability assay

Cells were seeded into 96-well plates at a density of 2 × 10^3^ cells/well. At 24, 48, and 72 h after being cultured, the medium was removed and replaced with 100 μl of experimental medium. In all, 10 μl CCK8 (1: 10, Dojindo Laboratories Co., Ltd, Kumamoto, Japan) solution was added to each well and the plates were incubated for 1–4 h at 37 °C. Absorbance was recorded at a wavelength of 450 nm using the mQuant ELISA Reader (Bio Tek Instruments, Inc., Winooski, VT, USA). OD values measured at different time points were used to draw the curve. The assays were conducted in triplicate.

### Colony formation assay

The treated cells were seeded into 6 cm culture dishes at a density of 1000 cells/dish and were cultured in DMEM supplemented with 10% FBS and 100 U/ml antibiotics at 37 °C in a 5% CO_2_ incubator. At day 12, cells were fixed with 4% paraformaldehyde and stained with 1% crystal violet. Subsequently, colonies with a diameter greater than 100μm were counted and analyzed using an Olympus BX-41 microscope. The experiment was performed in triplicate.

### Cell invasion assay

The transwell chambers (8μm in pore size, Corning, NY, USA) were placed into the 24-well transwell units and coated with Matrigel (BD Biosciences, NJ, USA). In total, 1 × 10^4^ (Fig. 2 and Supplementary Fig. 1) or 2 × 10^4^ (Fig. 3) cells/well were placed in the top chamber containing 200 μl of DMEM with 0.2% FBS, whereas the lower chambers received 600 μl medium containing 10% FBS. The chambers were incubated for 48 h at 37 °C in a 5% CO_2_ humidified incubator. Cells were scraped off the inner chamber, then the cells on the bottom of the membrance were fixed and counted. Images were captured with an Olympus BX-41 microscope (Olympus, Tokyo, Japan). Photographs of three random fields in each chamber were captured and the number of colonies was calculated.

### Wound healing assay

In all, 2 × 10^5^ cells were seeded into six-well plates and cultured to reach 95% confluence. A line was scratched across the monolayer cells with a 10 μl pipette tip. The cells were washed with PBS a total of three times and added into serum-free medium. After 36 h, the migration of cells was observed by phase contrast microscopy (Olympus BX-41). Pictures of three random fields in each triplicate well were captured to calculate the area of cell migration.

### Immunofluorescence assay

HepG2 and Hep3B cells were seeded onto coverslips, incubated 24 h and then fixed with 4% paraformaldehyde. Cells were incubated with primary antibodies (P300, 1:100, abcam, Cambridge, UK; Elk1, 1:100, abcam, Cambridge, UK) for 1 h at room temperature. CoraLite 488-conjugated secondary antibodies and CoraLite 594-conjugated secondary antibodies (Proteintech Group, Rosemont, IL, USA) were added for 30 min at room temperature at a dilution of 1:200 in PBS. Cells were then mounted with mounting medium (Vector Laboratories, Burlingame, CA, USA). Images were captured with a Carl Zeiss LSM 710 laser scanning confocal microscope (Carl Zeiss, Oberkochen, Germany).

### Flow cytometry

Cells (5 × 10^4^ cells/well) were seeded into six-well plates. The medium was replaced with 2 ml of experimental medium for 24 h, followed by serum-free experimental medium for an additional 24 h. After the cells were harvested, the rate of apoptosis was determined using an Annexin V-FITC/PI apoptosis detection kit (KeyGen Biotechnology Co., Ltd., Nanjing, China) according to the manufacturer’s instructions. Data analysis was performed using FlowJo software. After 48 h, the cells were collected and fixed in 70% ethanol overnight at 4 °C then stained with propidium iodide. A FACScan flow cytometer (Biosciences, San Jose, CA, USA) was utilized for cell cycle analysis. Data was analyzed using ModFit 3.0 software (Verity Software House, Topsham, ME, USA).

### Dual-luciferase reporter assay

The promoter sequence (−2004 to +208 bp) of aPKC-ι was cloned into a pGL3 basic vector to construct the pGL3-basic-pkci plasmid. The possible complementary binding region (CCCTGCAGGGAAGAGAC, predicted by http://alggen.lsi.upc.es; listed in Supplementary File 1) of Elk1 within the 180–164 bp region in the aPKC-ι promoter was deleted to construct the pGL3-basic-pkci-elk1-kt plasmid. The luciferase reporter plasmids were then transfected into hepatoma cells along with renilla reference plasmid using Lipofectamine 2000. Firefly and renilla luciferase activities were measured at 48 h using the Dual-Luciferase Reporter Assay System (Berthold Centro LB960, Wildbad, Germany). The assay was repeated three times.

### Chromatin immunoprecipitation assay

Chromatin immunoprecipitation (Ch-IP) assays were performed using SimpleChIP Plus Chromatin IP Kit (Cell Signaling, Massachusetts, USA) according to the manufacturer’s instructions. Hepatoma cells were transfected with recombinant plasmids to overexpress aPKC-ι promoter (pGL3-basic-pkci; WT group) or aPKC-ι promoter plasmids with a mutated Elk1 binding site (pGL3-basic-pkci-kt; MUT group). Cells were fixed with formaldehyde to crosslink DNA-proteins, chromatin was sheared using Microson Ultrasonic Cell Disruptor (Misonix, New York, USA) and incubated with antibodies, and IPs were bound to Protein G magnetic beads. The protein-DNA cross-link was reversed, DNA was purified, and enrichment of DNA sequences was detected using PCR and primers (listed in Supplementary File 1). Cell lysates without immunoprecipitation were analyzed with PCR as the positive control (Input), and DNA precipitated by IgG were used as the negative control.

### In vivo tumorigenicity assays

The mice were cared for in strict accordance with the institutional guidelines for animal care and the experiment was approved by the Committee on the Ethics of Animal Care and Use (certification listed in Supplementary File 4). Four to six-week-old male BALB/c (*nu/nu*) mice under specific pathogen-free (SPF) conditions were randomly divided into four groups (5 mice per group). Hepatoma cells (2 × 10^6^ cells) were subcutaneously injected into the upper right flank of nude mice. All mice were observed every 3 days and euthanized after 4 weeks. The tumor volumes were recorded and calculated as previously reported^21^.

### Co-immunoprecipitation (Co-IP) assay

Cells were collected after 48 h and lysed with buffer (1% NP-40, 0.5% sodium deoxycholate, 0.1% SDS), then the lysates were centrifuged at 14,000 g for 10 min at 4 °C. The supernatant was collected and incubated with Anti-Flag antibody overnight at 4 °C, followed by 3 h incubation with protein A/G plus-agarose beads (Beyotime Institute of Biotechnology, Jiangsu, China) at 4 °C. Bead-binding proteins were eluted by boiling in 1x SDS loading buffer for SDS-PAGE and further immunoblot analysis was performed with indicated antibodies. Cell lysates without immunoprecipitation underwent immunoblot analysis with indicated antibodies as the positive control, and proteins precipitated by IgG were used as the negative control.

### Statistical analysis

All experiments were repeated at least three times. The significance of quantitative data was assessed by the two-tailed Student’s *t*-test and one-way ANOVA or Pearson’s correlation test. Categorical data were analyzed using the *χ*^2^ test, and the survival among the subgroups was determined using Kaplan–Meier and log-rank analyses. The univariate and multivariate analyses were assessed by a Cox proportional hazards model. *P* < 0.05 was considered statistically significant.

## Supplementary information

Supplementary table 1

Supplementary table 2

Supplementary table 3

Supplementary table 4

Supplementary figure 1

Supplementary figure legends

Preliminary experiments, plasmids, viruses and gene sequences.

Cell Line Hep 3B STR Profile Report

Cell Line Hep G2 STR Profile Report

Supplementary File 4
