# Supplementary material for: Transcription co-activator P300 activates Elk1-aPKC-ι signaling mediated epithelial-to-mesenchymal transition and malignancy in hepatocellular carcinoma
Source: Oncogenesis. 2020 Mar 6;9(3):32. doi: 10.1038/s41389-020-0212-5 (PMC7060348; doi:10.1038/s41389-020-0212-5)
Supplement: Supplementary file 1 — Supplementary table 1 [file 41389_2020_212_MOESM1_ESM.docx]

**Supplementary table 1. Correlation between P300, aPKC-ι and clinicopathologic characteristics in 76 HCC patients**

| **Group** | **N** | **P300** | | ***X^2^*** | ***P* value** | **aPKC-ι** | | ***X^2^*** | ***P* value** |
| --- | --- | --- | --- | --- | --- | --- | --- | --- | --- |
|  |  | **Low** | **High** |  |  | **Low** | **High** |  |  |
| **Age(y)** | | | | | | | | | |
| ≤ 45 | 26 | 8 | 18 |  |  | 6 | 20 |  |  |
| > 45 | 50 | 20 | 30 | 0.626^a^ | 0.429 | 18 | 32 | 1.322^a^ | 0.250 |
| **Gender** | | | | | | | | | |
| Male | 65 | 24 | 41 |  |  | 21 | 44 |  |  |
| Female | 11 | 4 | 7 | 0.001^a^ | 0.972 | 3 | 8 | 0.110^a^ | 0.740 |
| **Carcinoma/Pericarcinoma tissues** | | | | | | | | | |
| Carcinoma | 76 | 28 | 48 |  |  | 24 | 52 |  |  |
| Pericarcinoma | 76 | 51 | 25 | 13.943^a^ | **<0.001** | 39 | 37 | 6.100^a^ | **0.014** |
| **Tumor size** | | | | | | | | | |
| ≤5cm | 36 | 14 | 22 |  |  | 19 | 17 |  |  |
| >5cm | 40 | 14 | 26 | 0.123^a^ | 0.726 | 5 | 35 | 14.226^a^ | **<0.001** |
| **AFP** | | | | | | | | | |
| <20 ng/ml | 25 | 15 | 10 |  |  | 14 | 11 |  |  |
| ≥20 ng/ml | 51 | 13 | 38 | 8.586^a^ | **0.003** | 10 | 41 | 10.283^a^ | **0.001** |
| **Vascular invasion** | | | | | | | | | |
| No | 62 | 25 | 37 |  |  | 23 | 39 |  |  |
| Yes | 14 | 3 | 11 | 1.752^a^ | 0.186 | 1 | 13 | 4.743^a^ | **0.029** |
| **TNM stage** | | | | | | | | | |
| I - II | 51 | 24 | 27 |  |  | 21 | 30 |  |  |
| III - IV | 25 | 4 | 21 | 6.955^a^ | **0.008** | 3 | 22 | 6.610^a^ | **0.010** |
| **Differentiation** | | | | | | | | | |
| Well | 20 | 15 | 5 |  |  | 13 | 7 |  |  |
| Medium/Poorly | 56 | 13 | 43 | 16.984^a^ | **<0.001** | 11 | 45 | 14.032^a^ | **<0.001** |

**AFP**: alpha fetoprotein; **TNM**: Tumor Node Metastasis.
