# Supplementary material for: Transcription co-activator P300 activates Elk1-aPKC-ι signaling mediated epithelial-to-mesenchymal transition and malignancy in hepatocellular carcinoma
Source: Oncogenesis. 2020 Mar 6;9(3):32. doi: 10.1038/s41389-020-0212-5 (PMC7060348; doi:10.1038/s41389-020-0212-5)
Supplement: Supplementary file 2 — Supplementary table 2 [file 41389_2020_212_MOESM2_ESM.docx]

**Supplementary table 2. Clinicopathologic characteristics of patients with Hepatocellular carcinoma**

| **Variables** | **（n=76）**  **Number** | **Percentage（%）** |
| --- | --- | --- |
| **Age (y)** |  |  |
| ≤ 45 | 26 | 34.21 |
| > 45 | 50 | 65.79 |
| **Gender** |  |  |
| Male | 65 | 85.53 |
| Female | 11 | 14.47 |
| **Tumor size** |  |  |
| ≤5cm | 36 | 47.37 |
| >5cm | 40 | 52.63 |
| **Tumor number** |  |  |
| Single | 65 | 85.53 |
| Multiple | 11 | 14.47 |
| **AFP** |  |  |
| <20 ng/ml | 25 | 32.89 |
| ≥20 ng/ml | 51 | 67.11 |
| **Vascular invasion** |  |  |
| No | 62 | 81.58 |
| Yes | 14 | 18.42 |
| **Adjacent metastasis** |  |  |
| Negative | 67 | 88.16 |
| Positive | 9 | 11.84 |
| **HBV** |  |  |
| Negative | 18 | 23.68 |
| Positive | 58 | 76.32 |
| **Child-Pugh classification** |  |  |
| A | 75 | 98.68 |
| B | 1 | 1.32 |
| **Operation** |  |  |
| R0 | 74 | 97.37 |
| R1 | 2 | 2.63 |
| **TNM stage** |  |  |
| I - II | 51 | 67.11 |
| III - IV | 25 | 32.89 |
| **Differentiation** |  |  |
| Well | 20 | 26.32 |
| Medium/Poorly | 56 | 73.68 |

**AFP**: alpha-fetoprotein; **TNM**: Tumor-Node-Metastasis.
