# Supplementary material for: Transcription co-activator P300 activates Elk1-aPKC-ι signaling mediated epithelial-to-mesenchymal transition and malignancy in hepatocellular carcinoma
Source: Oncogenesis. 2020 Mar 6;9(3):32. doi: 10.1038/s41389-020-0212-5 (PMC7060348; doi:10.1038/s41389-020-0212-5)
Supplement: Supplementary file 3 — Supplementary table 3 [file 41389_2020_212_MOESM3_ESM.docx]

**Numbers at risk of patients**

**P300：**

| Time after surgery (Months) | P300 low | P300 high |
| --- | --- | --- |
| 0.000 | 28. | 48. |
| 2.000 |  | 48. |
| 4.000 |  | 47. |
| 6.000 | 28. | 45. |
| 7.000 | 27. |  |
| 8.000 |  | 44. |
| 9.000 | 26. | 43. |
| 10.000 |  | 40. |
| 11.000 | 25. | 37. |
| 12.000 |  | 33. |
| 13.000 |  | 31. |
| 14.000 |  | 26. |
| 15.000 |  | 25. |
| 16.000 |  | 24. |
| 17.000 |  | 23. |
| 18.000 | 24. | 22. |
| 19.000 | 22. | 18. |
| 21.000 | 18. | 16. |
| 22.000 | 17. |  |
| 23.000 |  | 15. |
| 24.000 |  | 14. |
| 25.000 |  | 13. |
| 26.000 |  | 11. |
| 27.000 |  | 10. |
| 28.000 | 16. | 8. |
| 29.000 | 15. |  |
| 30.000 | 14. | 7. |
| 32.000 | 13. |  |
| 33.000 |  | 6. |
| 35.000 | 12. |  |
| 36.000 | 11. |  |
| 39.000 | 10. |  |
| 41.000 |  | 5. |
| 42.000 | 9. |  |
| 43.000 |  | 4. |
| 45.000 | 7. | 3. |
| 46.000 | 6. |  |
| 47.000 | 5. |  |
| 48.000 |  | 2. |
| 49.000 |  | 1. |
| 52.000 | 4. |  |
| 60.000 | 3. |  |

**aPKC-ι：**

| Time after surgery (Months) | aPKC-ι Low | aPKC-ι High |
| --- | --- | --- |
| 0.000 | 24. | 52. |
| 2.000 |  | 52. |
| 4.000 |  | 51. |
| 6.000 |  | 49. |
| 7.000 |  | 47. |
| 8.000 |  | 46. |
| 9.000 | 24. | 45. |
| 10.000 |  | 42. |
| 11.000 | 23. | 39. |
| 12.000 |  | 37. |
| 13.000 | 20. | 35. |
| 14.000 |  | 31. |
| 15.000 | 19. |  |
| 16.000 | 18. |  |
| 17.000 |  | 30. |
| 18.000 | 17. | 29. |
| 19.000 | 16. | 24. |
| 21.000 | 15. | 19. |
| 22.000 |  | 18. |
| 23.000 |  | 17. |
| 24.000 |  | 16. |
| 25.000 |  | 15. |
| 26.000 |  | 13. |
| 27.000 | 14. | 12. |
| 28.000 | 13. | 11. |
| 29.000 |  | 10. |
| 30.000 |  | 9. |
| 32.000 | 12. |  |
| 33.000 |  | 7. |
| 35.000 |  | 6. |
| 36.000 | 11. |  |
| 39.000 | 10. |  |
| 41.000 |  | 5. |
| 42.000 | 9. |  |
| 43.000 |  | 4. |
| 45.000 | 7. |  |
| 46.000 |  | 3. |
| 47.000 | 5. |  |
| 48.000 |  | 2. |
| 49.000 | 4. |  |
| 52.000 | 3. |  |
| 60.000 | 2. | 1. |

**P300+ aPKC-ι：**

| Time after surgery (Months) | P300^low^aPKC-ι^low^ | P300^high^aPKC-ι^high^ | P300^high^aPKC-ι^low^ + P300^low^aPKC-ι^high^ |
| --- | --- | --- | --- |
| 0.000 | 15. | 39. | 22. |
| 2.000 |  | 39. |  |
| 4.000 |  | 38. |  |
| 6.000 |  | 36. | 22. |
| 7.000 |  |  | 21. |
| 8.000 |  | 35. |  |
| 9.000 |  | 34. | 20. |
| 10.000 |  | 32. |  |
| 11.000 | 15. | 29. | 18. |
| 12.000 |  | 27. |  |
| 13.000 |  | 25. | 16. |
| 14.000 |  | 21. |  |
| 15.000 |  |  | 15. |
| 16.000 |  |  | 14. |
| 17.000 |  | 20. |  |
| 18.000 | 14. | 19. | 13. |
| 19.000 | 13. | 15. | 12. |
| 21.000 | 12. | 13. |  |
| 22.000 |  |  | 9. |
| 23.000 |  | 12. |  |
| 24.000 |  | 11. |  |
| 25.000 |  | 10. |  |
| 26.000 |  | 8. |  |
| 27.000 |  | 7. | 8. |
| 28.000 | 11. | 6. |  |
| 29.000 |  |  | 7. |
| 30.000 |  | 5. | 6. |
| 32.000 | 10. |  |  |
| 33.000 |  | 4. |  |
| 35.000 |  |  | 5. |
| 36.000 | 9. |  |  |
| 39.000 | 8. |  |  |
| 41.000 |  | 3. |  |
| 42.000 | 7. |  |  |
| 43.000 |  | 2. |  |
| 45.000 | 5. |  | 4. |
| 46.000 |  |  | 3. |
| 47.000 | 4. |  |  |
| 48.000 |  | 1. |  |
| 49.000 |  |  | 2. |
| 52.000 | 3. |  |  |
| 60.000 | 2. |  | 1. |
