# Supplementary material for: Transcription co-activator P300 activates Elk1-aPKC-ι signaling mediated epithelial-to-mesenchymal transition and malignancy in hepatocellular carcinoma
Source: Oncogenesis. 2020 Mar 6;9(3):32. doi: 10.1038/s41389-020-0212-5 (PMC7060348; doi:10.1038/s41389-020-0212-5)
Supplement: Supplementary file 4 — Supplementary table 4 [file 41389_2020_212_MOESM4_ESM.docx]

**Supplementary table 4. Univariate and multivariate analysis of factors associated with survival of 76 HCC patients**

|  | **Survival** | | |
| --- | --- | --- | --- |
|  | **Univariate analysis** | **Multivariate analysis** | |
|  | ***P* value** | **95%Cl** | ***P* value** |
| **Age** | 0.007 |  | 0.191 |
| **Gender** | 0.693 |  |  |
| **Tumor size** | **<0.001** | 1.542-5.192 | **0.001** |
| **AFP** | **0.003** | 1.632-5.396 | **<0.001** |
| **Vascular invasion** | **<0.001** |  | 0.216 |
| **Adjacent metastasis** | **0.001** |  | 0.564 |
| **TNM** | **<0.001** | 1.616-5.841 | **0.001** |
| **Differentiation** | **0.001** |  | 0.344 |
| **P300（low vs high）** | **0.001** | 1.020-3.541 | **0.043** |
| **aPKC-ι（low vs high）** | **0.008** |  | 0.595 |

**AFP**: alpha fetoprotein; **TNM**: Tumor Node Metastasis.
