# Supplementary figures and images for: Transcription co-activator P300 activates Elk1-aPKC-ι signaling mediated epithelial-to-mesenchymal transition and malignancy in hepatocellular carcinoma
Source: Oncogenesis. 2020 Mar 6;9(3):32. doi: 10.1038/s41389-020-0212-5 (PMC7060348; doi:10.1038/s41389-020-0212-5)

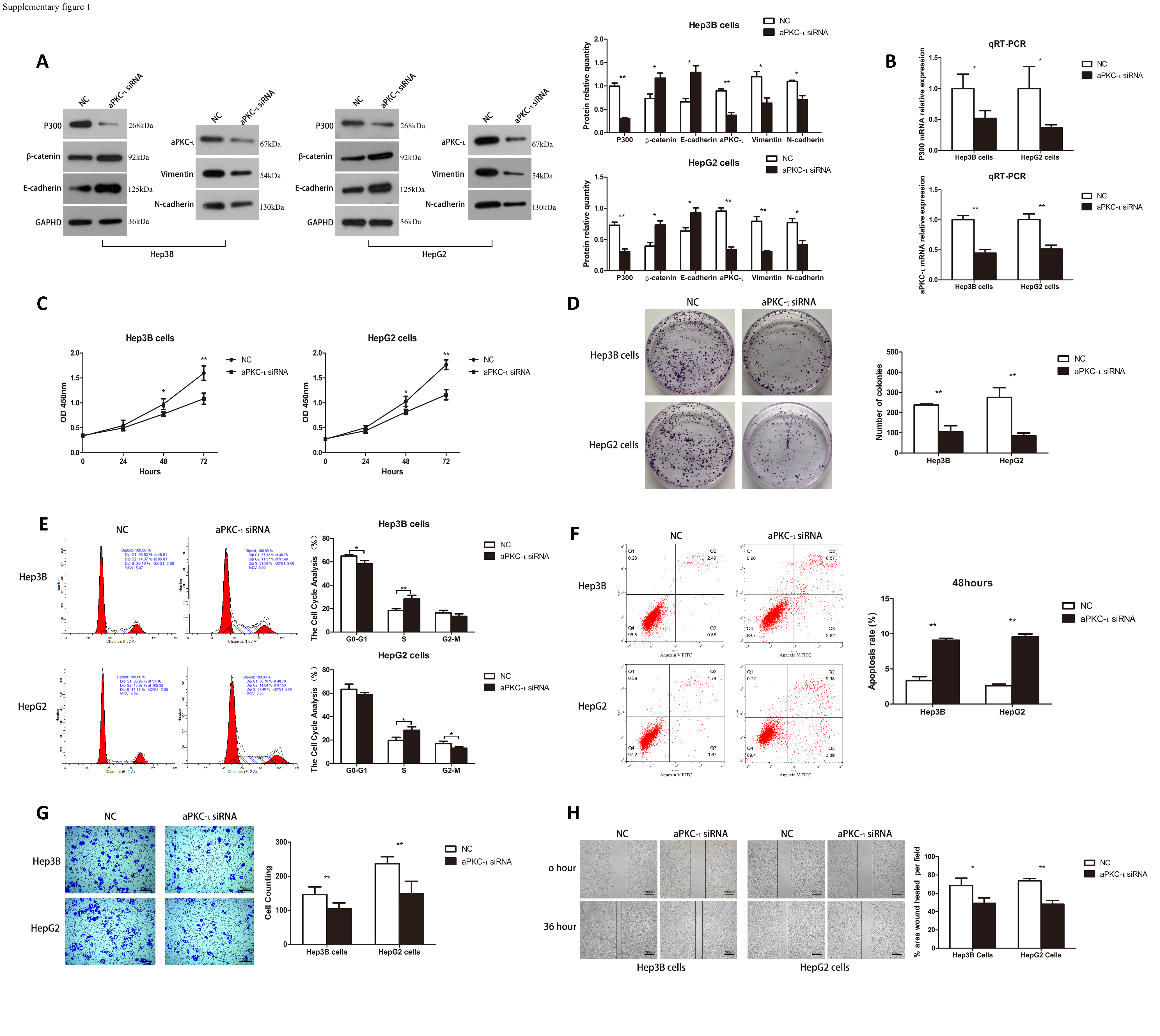

Supplement: Supplementary file 5 — Supplementary figure 1 [file 41389_2020_212_MOESM5_ESM.tif]

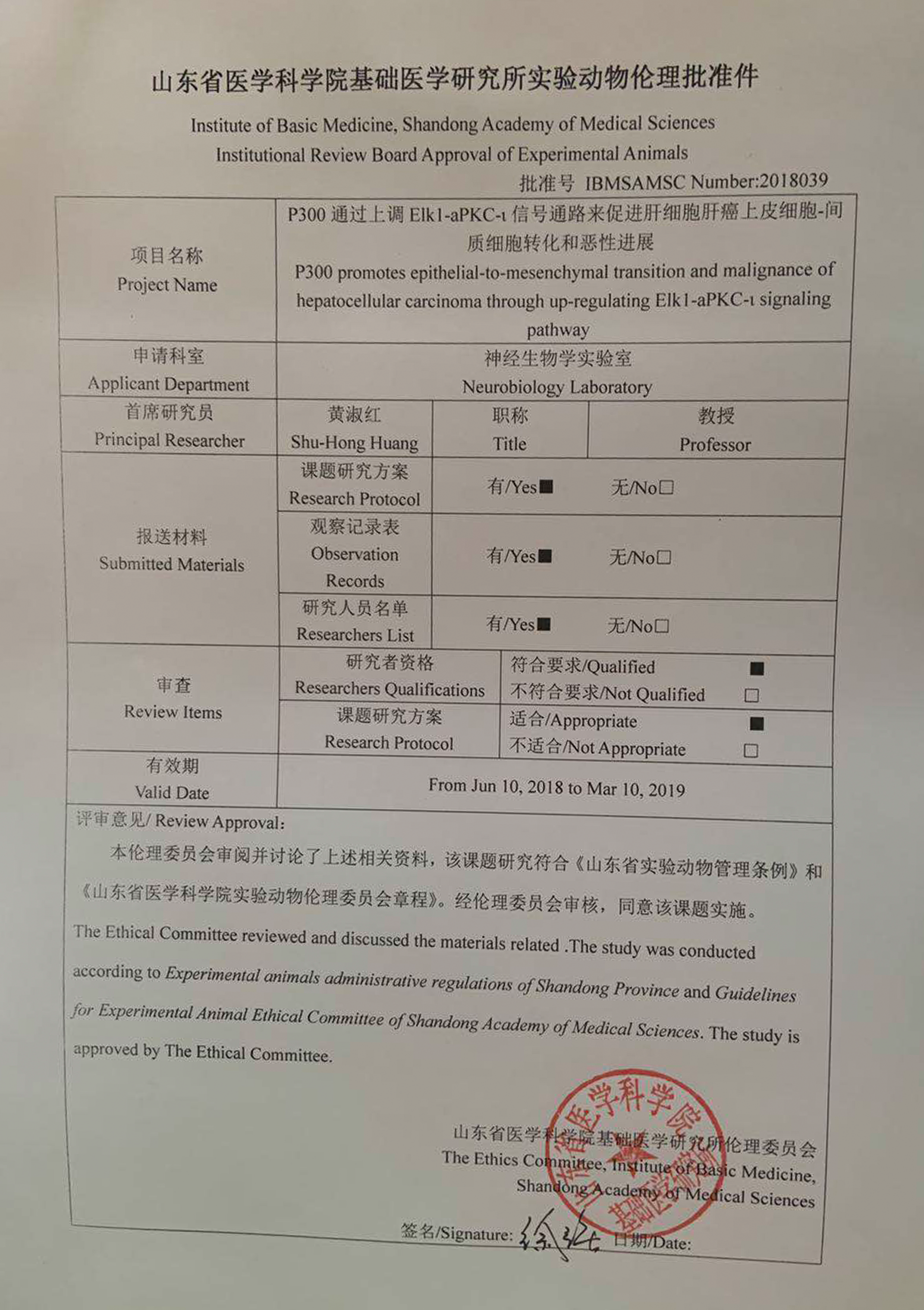

Supplement: Supplementary file 10 — Supplementary File 4 [file 41389_2020_212_MOESM10_ESM.tif]
