# Supplementary material for: Transcription co-activator P300 activates Elk1-aPKC-ι signaling mediated epithelial-to-mesenchymal transition and malignancy in hepatocellular carcinoma
Source: Oncogenesis. 2020 Mar 6;9(3):32. doi: 10.1038/s41389-020-0212-5 (PMC7060348; doi:10.1038/s41389-020-0212-5)
Supplement: Supplementary file 6 — Supplementary figure legends [file 41389_2020_212_MOESM6_ESM.docx]

**Supplementary figure 1. Knockdown of aPKC-ι reduced the expression of P300, inhibited EMT, proliferation, invasion and migration, and promoted apoptosis and cell cycle arrest in HCC cell lines. A.** WB was used to analyze the protein level of P300, aPKC-ι and EMT markers (E-cadherin, β-catenin, N-cadherin and vimentin) in Hep3B (left panel) and HepG2 (middle panel) cells. GAPDH was used as the internal control. Statistical analysis of relative optical density of each band was shown (right panel; n=3, Student’s t-test, mean ± SD, **P<0.05*, ***P<0.01*). **B.** qRT-PCR was used to analyze mRNA expression of P300 (upper panel) or aPKC-ι (lower panel) in Hep3B and HepG2 cells (n=3, Student’s t-test, mean ± SD, **P<0.05, **P<0.01*). **C.** CCK8 assay was used to investigate the proliferative kinetics of Hep3B (left panel) and HepG2 (right panel) cells (n=3, Student’s t-test, mean ± SD, **P<0.05, **P<0.01*). **D.** Colony formation assays were performed to evaluate the proliferative capability of Hep3B (upper panels) and HepG2 (lower panels) cells. A representative image was shown (left panels). Statistical comparison of the indicated groups was performed (right panel; n=3, Student’s t-test, mean ± SD, ***P<0.01*). **E.** FACS analysis was used to investigate differences in cell cycle distribution following aPKC-ι silencing. Representative images were shown in the left panels (Hep3B upper, HepG2 lower). The results were statistically analyzed in the right panels (n=3, Student’s t-test, mean ± SD, **P<0.05, **P<0.01*). **F.** Apoptosis of Hep3B (upper panels) and HepG2 (lower panels) cells was determined by flow cytometry. Representative images were shown in the left panels. The results were statistically analyzed in the right panel (n=3, Student’s t-test, mean ± SD, ***P<0.01*). **G.** Transwell invasion assay was used to evaluate the invasion ability of Hep3B (upper panels) and HepG2 (lower panels). Representative images were shown in the left panels (40X). Scale bar, 100 μm. The results are statistically analyzed in the right panel (n=3, Student’s t-test, mean ± SD, ***P<0.01*). **H.** Wound healing assay was performed to measure the migration ability of Hep3B and HepG2. Representative images were shown in the left panels (4X). Scale bar, 200 μm. The results were statistically analyzed in the right panel (n=3, Student’s t-test, mean ± SD, **P<0.05, **P<0.01*). For all experiments, cells transfected with aPKC-ι siRNA lentivirus were used as the experimental group, while cells transfected with empty vector were used as the negative control (NC).
