# Supplementary material for: Transcription co-activator P300 activates Elk1-aPKC-ι signaling mediated epithelial-to-mesenchymal transition and malignancy in hepatocellular carcinoma
Source: Oncogenesis. 2020 Mar 6;9(3):32. doi: 10.1038/s41389-020-0212-5 (PMC7060348; doi:10.1038/s41389-020-0212-5)

**1. Preliminary experiments to determine the abundance of P300 and PKC expression in different tumor cells**

**qRT-PCR results：**

| **Cells** | **GAPDH** | **EP300** | **∆Ct** | **Reference genes duplication** | **Target gene**  **duplication** |
| --- | --- | --- | --- | --- | --- |
| **HEP3B** | **13.31** | **19.78** | **6.47** | **0.160** | **0.100** |
|  | **13.43** | **19.76** | **6.33** |  |  |
|  | **13.47** | **19.86** | **6.39** |  |  |
| **HEPG2** | **13.05** | **20.54** | **7.49** | **0.140** | **0.380** |
|  | **12.95** | **20.25** | **7.3** |  |  |
|  | **12.91** | **20.16** | **7.25** |  |  |
| **BEL-7402** | **12.76** | **21.43** | **8.67** | **0.150** | **0.160** |
|  | **12.73** | **21.43** | **8.7** |  |  |
|  | **12.61** | **21.27** | **8.66** |  |  |
| **SMMC-7721** | **12.14** | **22.63** | **10.49** | **0.330** | **0.320** |
|  | **12.42** | **22.44** | **10.02** |  |  |
|  | **12.09** | **22.31** | **10.22** |  |  |
| **SK-HEP-1** | **12.99** | **29.05** | **16.06** | **0.220** | **0.360** |
|  | **12.78** | **28.78** | **16** |  |  |
|  | **12.77** | **28.69** | **15.92** |  |  |

| **Cells** | **GAPDH** | **PRKCI** | **∆Ct** | **Reference genes duplication** | **Target gene**  **duplication** |
| --- | --- | --- | --- | --- | --- |
| **HEP3B** | **13.31** | **21.22** | **7.91** | **0.160** | **0.310** |
|  | **13.43** | **21.29** | **7.86** |  |  |
|  | **13.47** | **20.98** | **7.51** |  |  |
| **HEPG2** | **13.05** | **22.17** | **9.12** | **0.140** | **0.230** |
|  | **12.95** | **21.94** | **8.99** |  |  |
|  | **12.91** | **22.02** | **9.11** |  |  |
| **BEL-7402** | **12.76** | **20.81** | **8.05** | **0.150** | **0.070** |
|  | **12.73** | **20.84** | **8.11** |  |  |
|  | **12.61** | **20.88** | **8.27** |  |  |
| **SMMC-7721** | **12.14** | **20.74** | **8.6** | **0.330** | **0.060** |
|  | **12.42** | **20.75** | **8.33** |  |  |
|  | **12.09** | **20.8** | **8.71** |  |  |
| **SK-HEP-1** | **12.99** | **23.95** | **10.96** | **0.220** | **0.500** |
|  | **12.78** | **23.61** | **10.83** |  |  |
|  | **12.77** | **23.45** | **10.68** |  |  |

**Notes**

**ΔCt=Ct value of target gene-CT value of internal reference gene；**

**ΔCt value ≤12，The gene expression in this cell was high；**

**12<ΔCt value<16，The gene expression in this cell was middle；**

**ΔCt value ≥16，The gene expression in this cell was low。**

**2. qRT PCR primers:**

**Sequences of gene-specific primers used for qRT-PCR**

| **Gene** | **Forward (5’-3’)** | **Reverse (5’-3’)** |
| --- | --- | --- |
| aPKC-ι | CCACACTTTCCAAGCCAAGC | ATGGGCATCACTGGTTCCTG |
| P300 | TGGAACAGGAGGAAGAAGAG | GAGAGGTCGTTAGATACATTGG |
| Elk1 | CACATCATCTCCTGGACTTCAC | CGGCTGAGCTTGTCGTAAT |
| β-actin | CCCGAGCCGTGTTTCCT | GTCCCAGTTGGTGACGATGC |
| aPKC-ι Promoter (WT) | GCAGGGAAGAGACTTTAG | CCATCCTTACGGGGGCAG |
| aPKC-ι Promoter (MUT) | TGTCCCTACCATTGTTTTAG | CCATCCTTACGGGGGCAG |

**3.** **shRNA lentivirus (hU6-MCS-CMV-Puromycin)**

**(1) The name of the gene：**PRKCI

**(2) Species：**Human

**(3) Easy-siRNA design：**

| **NO.** | **Accession** | **Target Seq** | **CDS** | **GC%** |
| --- | --- | --- | --- | --- |
| PRKCI-RNAi(26223-1) | NM_002740 | TGAAGAACATGCCAGATTT | 239..2029 | 36.84% |
| PRKCI-RNAi(26224-1) | NM_002740 | AGTCTAGGTCTTCAGGATT | 239..2029 | 42.11% |
| PRKCI-RNAi(26225-1) | NM_002740 | TTTAGACTTTATGAGCTAA | 239..2029 | 26.32% |
| Description | Homo sapiens protein kinase C, iota (PRKCI), mRNA. | | |  |

**(4) Objective lentivirus vector**

Name of the plasmid：GV112

Element order：hU6-MCS-CMV-Puromycin

Plasmid map：http://www.genechem.com.cn/Zaiti.aspx?zt=GV112


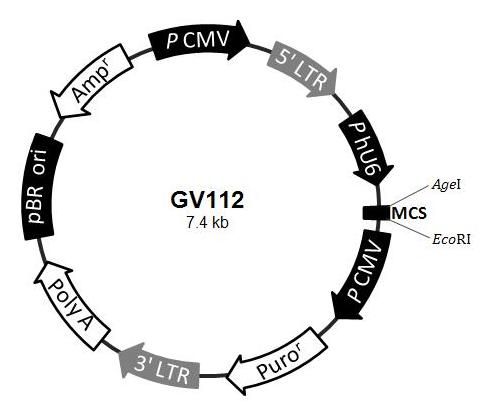


**(5) To synthesize oligo information:**

| **NO.** | **5’** | **STEM** | **Loop** | **STEM** | **3’** |
| --- | --- | --- | --- | --- | --- |
| PRKCI-RNAi(26223-1)-a | Ccgg | ccTGAAGAACATGCCAGATTT | CTCGAG | AAATCTGGCATGTTCTTCAGG | TTTTTg |
| PRKCI-RNAi(26223-1)-b | aattcaaaaa | ccTGAAGAACATGCCAGATTT | CTCGAG | AAATCTGGCATGTTCTTCAGG |  |
| PRKCI-RNAi(26224-1)-a | Ccgg | ccAGTCTAGGTCTTCAGGATT | CTCGAG | AATCCTGAAGACCTAGACTGG | TTTTTg |
| PRKCI-RNAi(26224-1)-b | aattcaaaaa | ccAGTCTAGGTCTTCAGGATT | CTCGAG | AATCCTGAAGACCTAGACTGG |  |
| PRKCI-RNAi(26225-1)-a | Ccgg | ccTTTAGACTTTATGAGCTAA | CTCGAG | TTAGCTCATAAAGTCTAAAGG | TTTTTg |
| PRKCI-RNAi(26225-1)-b | aattcaaaaa | ccTTTAGACTTTATGAGCTAA | CTCGAG | TTAGCTCATAAAGTCTAAAGG |  |

**(6) Sequencing result file：**

| **ID** | **Sequencing result file** |
| --- | --- |
| PRKCI-RNAi(26223-1) | PSC26223-1.rar;  1756339.31.S1312120819.PSC26223-1.PGCSIL-F(Sequencing OK).doc |
| PRKCI-RNAi(26224-1) | PSC26224-1.rar;  1756339.40.S1312120828.PSC26224-1.PGCSIL-F(Sequencing OK).doc |
| PRKCI-RNAi(26225-1) | PSC26225-1.rar;  1756339.23.S1312120811.PSC26225-1.PGCSIL-F(Sequencing OK).doc |

**(7) Titration：**

| **The virus name** | **titers (TU/mL)** |
| --- | --- |
| LV-PRKCI-RNAi(26223-1) | 1E+9 |
| LV-PRKCI-RNAi(26224-1) | 8E+8 |
| LV-PRKCI-RNAi(26225-1) | 6E+8 |

**4. shRNA lentivirus (hU6-MCS-Ubiquitin-EGFP-IRES-puromycin)**

**(1) siRNA design：(refer to: Treand C et al. Requirement for SWI/SNF chromatin-remodeling complex in Tat-mediated activation of the HIV-1 promoter. The EMBO journal 2006; 25: 1690-1699)**

| **Target Seq** | **GC%** |
| --- | --- |
| CAGAGCAGTCCTGGATTAG | 52.63% |

**(2) Objective lentivirus vector**

Name of the plasmid：GV248

Element order：hU6-MCS-Ubiquitin-EGFP-IRES-puromycin

Reference number：CON077

Contrast insertion sequence：TTCTCCGAACGTGTCACGT

Plasmid map：http://www.genechem.com.cn/Zaiti.aspx?zt=GV248


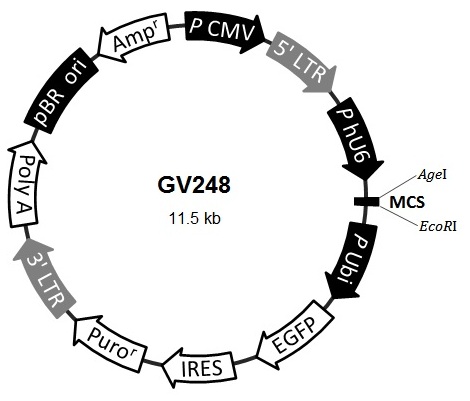


**(3) To synthesize oligo information:**

| **NO.** | **5’** | **STEM** | **Loop** | **STEM** | **3’** |
| --- | --- | --- | --- | --- | --- |
| EP300-RNAi(47783-1)-a | Ccgg | CAGAGCAGTCCTGGATTAG | CTCGAG | CTAATCCAGGACTGCTCTG | TTTTTg |
| EP300-RNAi(47783-1)-b | aattcaaaaa | CAGAGCAGTCCTGGATTAG | CTCGAG | CTAATCCAGGACTGCTCTG |  |

**(4) Sequencing result file：**

| **ID** | **Sequencing result file** |
| --- | --- |
| EP300-RNAi(47783-1) | PSC47783-1.rar; PSC47783-1.PGCSIL-F_B10(Sequencing OK).doc |

**(5) Titration：**

| **The virus name** | **titers (TU/mL)** |
| --- | --- |
| LV-EP300-RNAi(47783-1) | 3E+8 |

**5. aPKC-ι (PRKCI) over expressed plasmid map：**


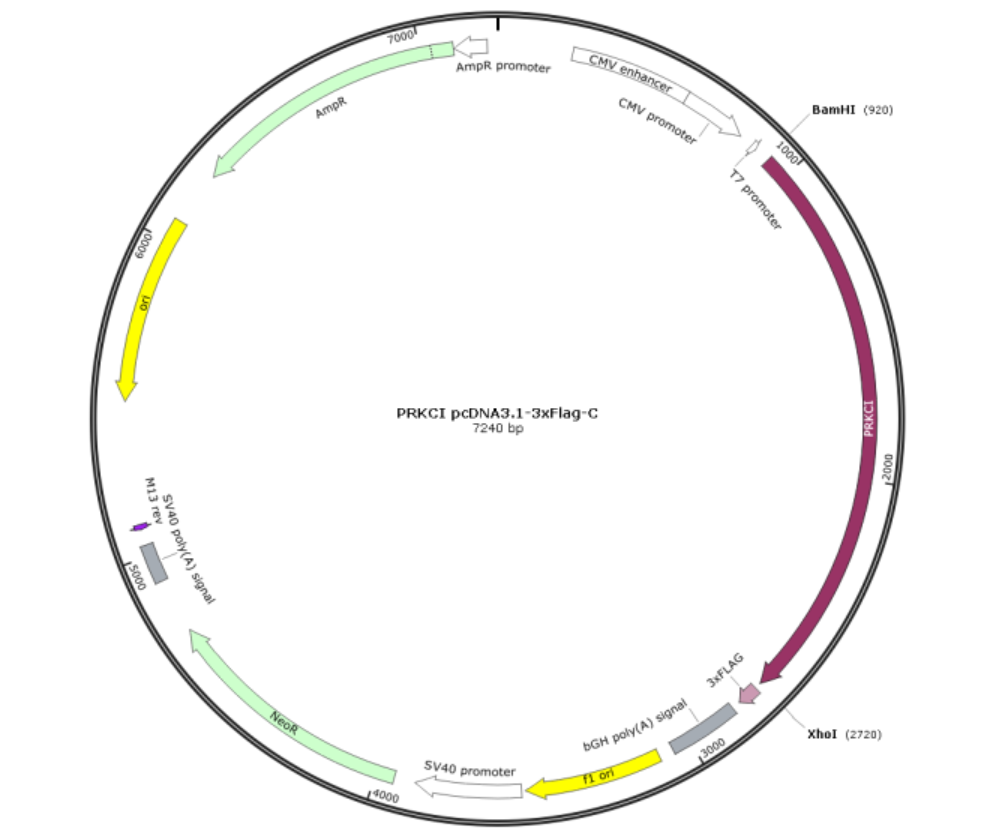


**Plasmid sequence (the red parts are aPKC-ι sequences):**

GACGGATCGGGAGATCTCCCGATCCCCTATGGTGCACTCTCAGTACAATCTGCTCTGATGCCGCATAGTTAAGCCAGTATCTGCTCCCTGCTTGTGTGTTGGAGGTCGCTGAGTAGTGCGCGAGCAAAATTTAAGCTACAACAAGGCAAGGCTTGACCGACAATTGCATGAAGAATCTGCTTAGGGTTAGGCGTTTTGCGCTGCTTCGCGATGTACGGGCCAGATATACGCGTTGACATTGATTATTGACTAGTTATTAATAGTAATCAATTACGGGGTCATTAGTTCATAGCCCATATATGGAGTTCCGCGTTACATAACTTACGGTAAATGGCCCGCCTGGCTGACCGCCCAACGACCCCCGCCCATTGACGTCAATAATGACGTATGTTCCCATAGTAACGCCAATAGGGACTTTCCATTGACGTCAATGGGTGGAGTATTTACGGTAAACTGCCCACTTGGCAGTACATCAAGTGTATCATATGCCAAGTACGCCCCCTATTGACGTCAATGACGGTAAATGGCCCGCCTGGCATTATGCCCAGTACATGACCTTATGGGACTTTCCTACTTGGCAGTACATCTACGTATTAGTCATCGCTATTACCATGGTGATGCGGTTTTGGCAGTACATCAATGGGCGTGGATAGCGGTTTGACTCACGGGGATTTCCAAGTCTCCACCCCATTGACGTCAATGGGAGTTTGTTTTGGCACCAAAATCAACGGGACTTTCCAAAATGTCGTAACAACTCCGCCCCATTGACGCAAATGGGCGGTAGGCGTGTACGGTGGGAGGTCTATATAAGCAGAGCTCTCTGGCTAACTAGAGAACCCACTGCTTACTGGCTTATCGAAATTAATACGACTCACTATAGGGAGACCCAAGCTGGCTAGTTAAGCTTGGTACCGAGCTCGGATCCGCCACCATGCCGACCCAGAGGGACAGCAGCACCATGTCCCACACGGTCGCAGGCGGCGGCAGCGGGGACCATTCCCACCAGGTCCGGGTGAAAGCCTACTACCGCGGGGATATCATGATAACACATTTTGAACCTTCCATCTCCTTTGAGGGCCTTTGCAATGAGGTTCGAGACATGTGTTCTTTTGACAACGAACAGCTCTTCACCATGAAATGGATAGATGAGGAAGGAGACCCGTGTACAGTATCATCTCAGTTGGAGTTAGAAGAAGCCTTTAGACTTTATGAGCTAAACAAGGATTCTGAACTCTTGATTCATGTGTTCCCTTGTGTACCAGAACGTCCTGGGATGCCTTGTCCAGGAGAAGATAAATCCATCTACCGTAGAGGTGCACGCCGCTGGAGAAAGCTTTATTGTGCCAATGGCCACACTTTCCAAGCCAAGCGTTTCAACAGGCGTGCTCACTGTGCCATCTGCACAGACCGAATATGGGGACTTGGACGCCAAGGATATAAGTGCATCAACTGCAAACTCTTGGTTCATAAGAAGTGCCATAAACTCGTCACAATTGAATGTGGGCGGCATTCTTTGCCACAGGAACCAGTGATGCCCATGGATCAGTCATCCATGCATTCTGACCATGCACAGACAGTAATTCCATATAATCCTTCAAGTCATGAGAGTTTGGATCAAGTTGGTGAAGAAAAAGAGGCAATGAACACCAGGGAAAGTGGCAAAGCTTCATCCAGTCTAGGTCTTCAGGATTTTGATTTGCTCCGGGTAATAGGAAGAGGAAGTTATGCCAAAGTACTGTTGGTTCGATTAAAAAAAACAGATCGTATTTATGCAATGAAAGTTGTGAAAAAAGAGCTTGTTAATGATGATGAGGATATTGATTGGGTACAGACAGAGAAGCATGTGTTTGAGCAGGCATCCAATCATCCTTTCCTTGTTGGGCTGCATTCTTGCTTTCAGACAGAAAGCAGATTGTTCTTTGTTATAGAGTATGTAAATGGAGGAGACCTAATGTTTCATATGCAGCGACAAAGAAAACTTCCTGAAGAACATGCCAGATTTTACTCTGCAGAAATCAGTCTAGCATTAAATTATCTTCATGAGCGAGGGATAATTTATAGAGATTTGAAACTGGACAATGTATTACTGGACTCTGAAGGCCACATTAAACTCACTGACTACGGCATGTGTAAGGAAGGATTACGGCCAGGAGATACAACCAGCACTTTCTGTGGTACTCCTAATTACATTGCTCCTGAAATTTTAAGAGGAGAAGATTATGGTTTCAGTGTTGACTGGTGGGCTCTTGGAGTGCTCATGTTTGAGATGATGGCAGGAAGGTCTCCATTTGATATTGTTGGGAGCTCCGATAACCCTGACCAGAACACAGAGGATTATCTCTTCCAAGTTATTTTGGAAAAACAAATTCGCATACCACGTTCTCTGTCTGTAAAAGCTGCAAGTGTTCTGAAGAGTTTTCTTAATAAGGACCCTAAGGAACGATTGGGTTGTCATCCTCAAACAGGATTTGCTGATATTCAGGGACACCCGTTCTTCCGAAATGTTGATTGGGATATGATGGAGCAAAAACAGGTGGTACCTCCCTTTAAACCAAATATTTCTGGGGAATTTGGTTTGGACAACTTTGATTCTCAGTTTACTAATGAACCTGTCCAGCTCACTCCAGATGACGATGACATTGTGAGGAAGATTGATCAGTCTGAATTTGAAGGTTTTGAGTATATCAATCCTCTTTTGATGTCTGCAGAAGAATGTGTCCTCGAGTCTAGAGGGCCCTTCGACTACAAAGACCATGACGGTGATTATAAAGATCATGACATCGACTACAAGGATGACGATGACAAGTGAGTTTAAACCCGCTGATCAGCCTCGACTGTGCCTTCTAGTTGCCAGCCATCTGTTGTTTGCCCCTCCCCCGTGCCTTCCTTGACCCTGGAAGGTGCCACTCCCACTGTCCTTTCCTAATAAAATGAGGAAATTGCATCGCATTGTCTGAGTAGGTGTCATTCTATTCTGGGGGGTGGGGTGGGGCAGGACAGCAAGGGGGAGGATTGGGAAGACAATAGCAGGCATGCTGGGGATGCGGTGGGCTCTATGGCTTCTGAGGCGGAAAGAACCAGCTGGGGCTCTAGGGGGTATCCCCACGCGCCCTGTAGCGGCGCATTAAGCGCGGCGGGTGTGGTGGTTACGCGCAGCGTGACCGCTACACTTGCCAGCGCCCTAGCGCCCGCTCCTTTCGCTTTCTTCCCTTCCTTTCTCGCCACGTTCGCCGGCTTTCCCCGTCAAGCTCTAAATCGGGGGCTCCCTTTAGGGTTCCGATTTAGTGCTTTACGGCACCTCGACCCCAAAAAACTTGATTAGGGTGATGGTTCACGTAGTGGGCCATCGCCCTGATAGACGGTTTTTCGCCCTTTGACGTTGGAGTCCACGTTCTTTAATAGTGGACTCTTGTTCCAAACTGGAACAACACTCAACCCTATCTCGGTCTATTCTTTTGATTTATAAGGGATTTTGCCGATTTCGGCCTATTGGTTAAAAAATGAGCTGATTTAACAAAAATTTAACGCGAATTAATTCTGTGGAATGTGTGTCAGTTAGGGTGTGGAAAGTCCCCAGGCTCCCCAGCAGGCAGAAGTATGCAAAGCATGCATCTCAATTAGTCAGCAACCAGGTGTGGAAAGTCCCCAGGCTCCCCAGCAGGCAGAAGTATGCAAAGCATGCATCTCAATTAGTCAGCAACCATAGTCCCGCCCCTAACTCCGCCCATCCCGCCCCTAACTCCGCCCAGTTCCGCCCATTCTCCGCCCCATGGCTGACTAATTTTTTTTATTTATGCAGAGGCCGAGGCCGCCTCTGCCTCTGAGCTATTCCAGAAGTAGTGAGGAGGCTTTTTTGGAGGCCTAGGCTTTTGCAAAAAGCTCCCGGGAGCTTGTATATCCATTTTCGGATCTGATCAAGAGACAGGATGAGGATCGTTTCGCATGATTGAACAAGATGGATTGCACGCAGGTTCTCCGGCCGCTTGGGTGGAGAGGCTATTCGGCTATGACTGGGCACAACAGACAATCGGCTGCTCTGATGCCGCCGTGTTCCGGCTGTCAGCGCAGGGGCGCCCGGTTCTTTTTGTCAAGACCGACCTGTCCGGTGCCCTGAATGAACTGCAGGACGAGGCAGCGCGGCTATCGTGGCTGGCCACGACGGGCGTTCCTTGCGCAGCTGTGCTCGACGTTGTCACTGAAGCGGGAAGGGACTGGCTGCTATTGGGCGAAGTGCCGGGGCAGGATCTCCTGTCATCTCACCTTGCTCCTGCCGAGAAAGTATCCATCATGGCTGATGCAATGCGGCGGCTGCATACGCTTGATCCGGCTACCTGCCCATTCGACCACCAAGCGAAACATCGCATCGAGCGAGCACGTACTCGGATGGAAGCCGGTCTTGTCGATCAGGATGATCTGGACGAAGAGCATCAGGGGCTCGCGCCAGCCGAACTGTTCGCCAGGCTCAAGGCGCGCATGCCCGACGGCGAGGATCTCGTCGTGACCCATGGCGATGCCTGCTTGCCGAATATCATGGTGGAAAATGGCCGCTTTTCTGGATTCATCGACTGTGGCCGGCTGGGTGTGGCGGACCGCTATCAGGACATAGCGTTGGCTACCCGTGATATTGCTGAAGAGCTTGGCGGCGAATGGGCTGACCGCTTCCTCGTGCTTTACGGTATCGCCGCTCCCGATTCGCAGCGCATCGCCTTCTATCGCCTTCTTGACGAGTTCTTCTGAGCGGGACTCTGGGGTTCGCGAAATGACCGACCAAGCGACGCCCAACCTGCCATCACGAGATTTCGATTCCACCGCCGCCTTCTATGAAAGGTTGGGCTTCGGAATCGTTTTCCGGGACGCCGGCTGGATGATCCTCCAGCGCGGGGATCTCATGCTGGAGTTCTTCGCCCACCCCAACTTGTTTATTGCAGCTTATAATGGTTACAAATAAAGCAATAGCATCACAAATTTCACAAATAAAGCATTTTTTTCACTGCATTCTAGTTGTGGTTTGTCCAAACTCATCAATGTATCTTATCATGTCTGTATACCGTCGACCTCTAGCTAGAGCTTGGCGTAATCATGGTCATAGCTGTTTCCTGTGTGAAATTGTTATCCGCTCACAATTCCACACAACATACGAGCCGGAAGCATAAAGTGTAAAGCCTGGGGTGCCTAATGAGTGAGCTAACTCACATTAATTGCGTTGCGCTCACTGCCCGCTTTCCAGTCGGGAAACCTGTCGTGCCAGCTGCATTAATGAATCGGCCAACGCGCGGGGAGAGGCGGTTTGCGTATTGGGCGCTCTTCCGCTTCCTCGCTCACTGACTCGCTGCGCTCGGTCGTTCGGCTGCGGCGAGCGGTATCAGCTCACTCAAAGGCGGTAATACGGTTATCCACAGAATCAGGGGATAACGCAGGAAAGAACATGTGAGCAAAAGGCCAGCAAAAGGCCAGGAACCGTAAAAAGGCCGCGTTGCTGGCGTTTTTCCATAGGCTCCGCCCCCCTGACGAGCATCACAAAAATCGACGCTCAAGTCAGAGGTGGCGAAACCCGACAGGACTATAAAGATACCAGGCGTTTCCCCCTGGAAGCTCCCTCGTGCGCTCTCCTGTTCCGACCCTGCCGCTTACCGGATACCTGTCCGCCTTTCTCCCTTCGGGAAGCGTGGCGCTTTCTCATAGCTCACGCTGTAGGTATCTCAGTTCGGTGTAGGTCGTTCGCTCCAAGCTGGGCTGTGTGCACGAACCCCCCGTTCAGCCCGACCGCTGCGCCTTATCCGGTAACTATCGTCTTGAGTCCAACCCGGTAAGACACGACTTATCGCCACTGGCAGCAGCCACTGGTAACAGGATTAGCAGAGCGAGGTATGTAGGCGGTGCTACAGAGTTCTTGAAGTGGTGGCCTAACTACGGCTACACTAGAAGAACAGTATTTGGTATCTGCGCTCTGCTGAAGCCAGTTACCTTCGGAAAAAGAGTTGGTAGCTCTTGATCCGGCAAACAAACCACCGCTGGTAGCGGTGGTTTTTTTGTTTGCAAGCAGCAGATTACGCGCAGAAAAAAAGGATCTCAAGAAGATCCTTTGATCTTTTCTACGGGGTCTGACGCTCAGTGGAACGAAAACTCACGTTAAGGGATTTTGGTCATGAGATTATCAAAAAGGATCTTCACCTAGATCCTTTTAAATTAAAAATGAAGTTTTAAATCAATCTAAAGTATATATGAGTAAACTTGGTCTGACAGTTACCAATGCTTAATCAGTGAGGCACCTATCTCAGCGATCTGTCTATTTCGTTCATCCATAGTTGCCTGACTCCCCGTCGTGTAGATAACTACGATACGGGAGGGCTTACCATCTGGCCCCAGTGCTGCAATGATACCGCGAGACCCACGCTCACCGGCTCCAGATTTATCAGCAATAAACCAGCCAGCCGGAAGGGCCGAGCGCAGAAGTGGTCCTGCAACTTTATCCGCCTCCATCCAGTCTATTAATTGTTGCCGGGAAGCTAGAGTAAGTAGTTCGCCAGTTAATAGTTTGCGCAACGTTGTTGCCATTGCTACAGGCATCGTGGTGTCACGCTCGTCGTTTGGTATGGCTTCATTCAGCTCCGGTTCCCAACGATCAAGGCGAGTTACATGATCCCCCATGTTGTGCAAAAAAGCGGTTAGCTCCTTCGGTCCTCCGATCGTTGTCAGAAGTAAGTTGGCCGCAGTGTTATCACTCATGGTTATGGCAGCACTGCATAATTCTCTTACTGTCATGCCATCCGTAAGATGCTTTTCTGTGACTGGTGAGTACTCAACCAAGTCATTCTGAGAATAGTGTATGCGGCGACCGAGTTGCTCTTGCCCGGCGTCAATACGGGATAATACCGCGCCACATAGCAGAACTTTAAAAGTGCTCATCATTGGAAAACGTTCTTCGGGGCGAAAACTCTCAAGGATCTTACCGCTGTTGAGATCCAGTTCGATGTAACCCACTCGTGCACCCAACTGATCTTCAGCATCTTTTACTTTCACCAGCGTTTCTGGGTGAGCAAAAACAGGAAGGCAAAATGCCGCAAAAAAGGGAATAAGGGCGACACGGAAATGTTGAATACTCATACTCTTCCTTTTTCAATATTATTGAAGCATTTATCAGGGTTATTGTCTCATGAGCGGATACATATTTGAATGTATTTAGAAAAATAAACAAATAGGGGTTCCGCGCACATTTCCCCGAAAAGTGCCACCTGACGTC

**6. aPKC-ι (PKCI) promoter plasmid map:**


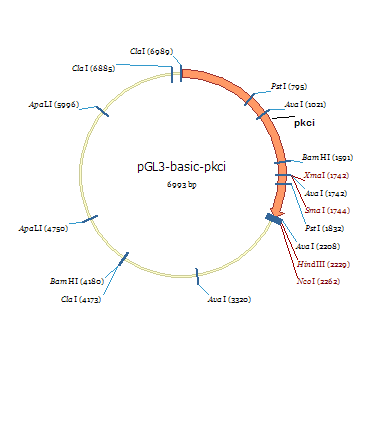


**(1) The promoter sequence (-2004～+208bp; the yellow parts) of aPKC-ι(pkci):**

**(2) the aPKC-ι transcription initiation sequence (the green part):**

**(3) the Elk 1 binding site (-180～-164bp; the blue part):**

| 1  51  101  151  201  251  301  351  401  451  501  551  601  651  701  751  801  851  901  951  1001  1051  1101  1151  1201  1251  1301  1351  1401  1451  1501  1551  1601  1651  1701  1751  1801  1851  1901  1951  2001  2051  2101  2151  2201  2251  2301  2351  2401  2451  2501  2551  2601  2651  2701  2751  2801  2851  2901  2951  3001  3051  3101  3151  3201  3251  3301  3351  3401  3451  3501  3551  3601  3651  3701  3751  3801  3851  3901  3951  4001  4051  4101  4151  4201  4251  4301  4351  4401  4451  4501  4551  4601  4651  4701  4751  4801  4851  4901  4951  5001  5051  5101  5151  5201  5251  5301  5351  5401  5451  5501  5551  5601  5651  5701  5751  5801  5851  5901  5951  6001  6051  6101  6151  6201  6251  6301  6351  6401  6451  6501  6551  6601  6651  6701  6751  6801  6851  6901  6951 | GGTACCAATA GAAAAACTCA TAGCAACAGA AAATATATTA TTGGTTGCCA  GAGGTTGAGG AGAGTGGGGA GTAACTGCTA ATGGATATGC AGTTACTTTC  AGGGTGCTGA AAATACTCTG GATTTAGGTA GAGGTGATGG TTGCAAAACT  TTGTGAAGAT ACTTAAAACT ATCAAATTTT ACACTTTAAA TGAGTAAGTT  GTATGGCATG TGAATTATCT TTTTTTAAAA AAATGAGACG GGATCTCACT  GTGCATTGTG TTGCCCAGGG ACGGAGTCTC GCTGGAGTGC AGTGGTGCAG  TCTCAGAGGC TCACAGCAAC CTCTGCCTCC TGGGTTTAAG CGATTCTCGT  GCCTCAGCCT CCGGAGTAGC TGGGATTACA GGTGCCCGTA TATTTTATAA  TATATTCCAT AATTAATCGC TCCGTATTAA ACTTGCTGAT GCAGGTCTCC  AATTACTGAA AAACCTGATA CCAAGTTTTT CAATGGGATT TTTTTGTTTG  TTTAAGAGAT GGGGTCTCAC CATGTTGCTC AGGCTGGCCT TGAACTCCTG  GGCTCAAGTG ATCCTCCAGC CTCAGCCTCT CAAAGGCTGG GACTACAGGC  ATGTAGCACT TTGCCTGACT CAAGGGGAAT TTTTAGTAAG AGTATATAAT  CCAAGTCTGT AGCAGTTGGA GGATGTCGTA CCACGCTGAA GTGTCACACA  TAACTGATGA CTCAGTCCCC CAAGCCTATT TTATCATCGT AAAGAAAGCT  ATTTCTTTGA TTATTCCAAC TCTTTCAGCA CCAGGACTGC TGCAGGGGCT  GGATTCATGC TACCTGTAGT TGCAGTGTAA ATTATGTGTC AGAGCCAAGA  TCCTAGGAAA GTCAAAGACT AGGTTACAAG AGCGATTCTT TTTTTTTTTT  TTTTTTTTTT TTTTTGAGAC AGAGTCTTGC TCTGTCGCCC AGGCTGGAGT  GCAGTGGCAC AGTCTTGGCT CACTGCAACC TCCGCCTCCC TGGTTCACGC  CATTCTCCTG CCTTAGCCTC CCGAGTAGCT GGGACTACAG GCGCCCGCCA  CCACGCCCGG CTAATTTTTT GTGTTTTTAG TAGAGACGGG GTTTCACCGT  GTTAGCCAGG ATGGTCTCGA TCTCCTGACC TTGTGATCCG CCCGCCTCGG  CCTCCCAAAG TGCTGGGATT ACAGGCTTGA GCCACCGCGC CCGGCCAAGG  ACGATTCTTT CTAATCTTTG GTAAGCCAGT TTTTACAGAA TGAAATGCTC  TTGGATTGAC TGACTTGATT GGGTAAGTAT TCATTGGGCT CTTGTTCCAT  GCAAGGGCAG TCTACAAGGG CCTAGATATG GGGGCCCCCA AAGAAGAAAA  ATCGGTTTTT CATCATATTT TCTGGTTGAG TTTCTTCCTT TATTATATTT  GTTACATATG TTATTAAATT ATTTCCATAA AAGAGGTTAT GCTGAGCAGG  TAACCAAATT CTTGTTCCCC CAAAAGACAT GTATAATTCA GACGATTTAT  GCAATTAGAA GAAGGGAGAC TTCATCAGTT AATAAAGTCT AGAACGTGAG  GAAGCCCAGT CATTTCCAGG ACCTTCAGAT GCCCAAGCTG GATCCAGGTT  TTCCACCCCG ACAGCTCCAG GGAGCACGCT GGAGAGACAG ACTTCACCAG  CGGGAAGGCG CGGCACCCTA CGCGCAAGGA ACCTCAAGTC CCAGAATCCT  ATGAGGACTC CAAATCCCAG AAACCTGCAC GCAAATCTAT CCCGGGTGCC  AAGTCGGCCG ACCGCAGACG TGACCAAGTT GCGAGGCTAG GGTGAGGTGT  CGTAGAATTT GTCCCTACCA TTGT**CCCTGC AGGGAAGAGA C**TTTAGTGAT  CTAGAGCTGG TCATTGTCTC TTTCACAGCT CGCGTGAAAG CACCCCTCTC  GGCGTGTTTG ATGCCATGAT CAGCGTCTCC CATTCCCTCT GTCCCCTTTC  TCTGCCCCCG TAAGGATGGT TCAGCCCGGT ATTGAGGCTC CTTGAGCTGG  CTGG**ATGAGT GGATCA**TTTT TCATATGTAG TATGTCAGAA ATTTGTATAA  GAACAAAGAT GCATCAAAAT TTGTTCAAGA AAAAGTGAAA ATTTCAGAGT  TTGGAATTTT GCTGTACTTA AAGCACCATT CTGGTAGAAC TGATGCTGTC  GTATTTACAT TTAATAATGT TACATTTAGC TGGACATAGT GGCTCACACA  GCACTTCTCG AGATCTGCGA TCTAAGTAAG CTTGGCATTC CGGTACTGTT  GGTAAAGCCA CCATGGAAGA CGCCAAAAAC ATAAAGAAAG GCCCGGCGCC  ATTCTATCCG CTGGAAGATG GAACCGCTGG AGAGCAACTG CATAAGGCTA  TGAAGAGATA CGCCCTGGTT CCTGGAACAA TTGCTTTTAC AGATGCACAT  ATCGAGGTGG ACATCACTTA CGCTGAGTAC TTCGAAATGT CCGTTCGGTT  GGCAGAAGCT ATGAAACGAT ATGGGCTGAA TACAAATCAC AGAATCGTCG  TATGCAGTGA AAACTCTCTT CAATTCTTTA TGCCGGTGTT GGGCGCGTTA  TTTATCGGAG TTGCAGTTGC GCCCGCGAAC GACATTTATA ATGAACGTGA  ATTGCTCAAC AGTATGGGCA TTTCGCAGCC TACCGTGGTG TTCGTTTCCA  AAAAGGGGTT GCAAAAAATT TTGAACGTGC AAAAAAAGCT CCCAATCATC  CAAAAAATTA TTATCATGGA TTCTAAAACG GATTACCAGG GATTTCAGTC  GATGTACACG TTCGTCACAT CTCATCTACC TCCCGGTTTT AATGAATACG  ATTTTGTGCC AGAGTCCTTC GATAGGGACA AGACAATTGC ACTGATCATG  AACTCCTCTG GATCTACTGG TCTGCCTAAA GGTGTCGCTC TGCCTCATAG  AACTGCCTGC GTGAGATTCT CGCATGCCAG AGATCCTATT TTTGGCAATC  AAATCATTCC GGATACTGCG ATTTTAAGTG TTGTTCCATT CCATCACGGT  TTTGGAATGT TTACTACACT CGGATATTTG ATATGTGGAT TTCGAGTCGT  CTTAATGTAT AGATTTGAAG AAGAGCTGTT TCTGAGGAGC CTTCAGGATT  ACAAGATTCA AAGTGCGCTG CTGGTGCCAA CCCTATTCTC CTTCTTCGCC  AAAAGCACTC TGATTGACAA ATACGATTTA TCTAATTTAC ACGAAATTGC  TTCTGGTGGC GCTCCCCTCT CTAAGGAAGT CGGGGAAGCG GTTGCCAAGA  GGTTCCATCT GCCAGGTATC AGGCAAGGAT ATGGGCTCAC TGAGACTACA  TCAGCTATTC TGATTACACC CGAGGGGGAT GATAAACCGG GCGCGGTCGG  TAAAGTTGTT CCATTTTTTG AAGCGAAGGT TGTGGATCTG GATACCGGGA  AAACGCTGGG CGTTAATCAA AGAGGCGAAC TGTGTGTGAG AGGTCCTATG  ATTATGTCCG GTTATGTAAA CAATCCGGAA GCGACCAACG CCTTGATTGA  CAAGGATGGA TGGCTACATT CTGGAGACAT AGCTTACTGG GACGAAGACG  AACACTTCTT CATCGTTGAC CGCCTGAAGT CTCTGATTAA GTACAAAGGC  TATCAGGTGG CTCCCGCTGA ATTGGAATCC ATCTTGCTCC AACACCCCAA  CATCTTCGAC GCAGGTGTCG CAGGTCTTCC CGACGATGAC GCCGGTGAAC  TTCCCGCCGC CGTTGTTGTT TTGGAGCACG GAAAGACGAT GACGGAAAAA  GAGATCGTGG ATTACGTCGC CAGTCAAGTA ACAACCGCGA AAAAGTTGCG  CGGAGGAGTT GTGTTTGTGG ACGAAGTACC GAAAGGTCTT ACCGGAAAAC  TCGACGCAAG AAAAATCAGA GAGATCCTCA TAAAGGCCAA GAAGGGCGGA  AAGATCGCCG TGTAATTCTA GAGTCGGGGC GGCCGGCCGC TTCGAGCAGA  CATGATAAGA TACATTGATG AGTTTGGACA AACCACAACT AGAATGCAGT  GAAAAAAATG CTTTATTTGT GAAATTTGTG ATGCTATTGC TTTATTTGTA  ACCATTATAA GCTGCAATAA ACAAGTTAAC AACAACAATT GCATTCATTT  TATGTTTCAG GTTCAGGGGG AGGTGTGGGA GGTTTTTTAA AGCAAGTAAA  ACCTCTACAA ATGTGGTAAA ATCGATAAGG ATCCGTCGAC CGATGCCCTT  GAGAGCCTTC AACCCAGTCA GCTCCTTCCG GTGGGCGCGG GGCATGACTA  TCGTCGCCGC ACTTATGACT GTCTTCTTTA TCATGCAACT CGTAGGACAG  GTGCCGGCAG CGCTCTTCCG CTTCCTCGCT CACTGACTCG CTGCGCTCGG  TCGTTCGGCT GCGGCGAGCG GTATCAGCTC ACTCAAAGGC GGTAATACGG  TTATCCACAG AATCAGGGGA TAACGCAGGA AAGAACATGT GAGCAAAAGG  CCAGCAAAAG GCCAGGAACC GTAAAAAGGC CGCGTTGCTG GCGTTTTTCC  ATAGGCTCCG CCCCCCTGAC GAGCATCACA AAAATCGACG CTCAAGTCAG  AGGTGGCGAA ACCCGACAGG ACTATAAAGA TACCAGGCGT TTCCCCCTGG  AAGCTCCCTC GTGCGCTCTC CTGTTCCGAC CCTGCCGCTT ACCGGATACC  TGTCCGCCTT TCTCCCTTCG GGAAGCGTGG CGCTTTCTCA TAGCTCACGC  TGTAGGTATC TCAGTTCGGT GTAGGTCGTT CGCTCCAAGC TGGGCTGTGT  GCACGAACCC CCCGTTCAGC CCGACCGCTG CGCCTTATCC GGTAACTATC  GTCTTGAGTC CAACCCGGTA AGACACGACT TATCGCCACT GGCAGCAGCC  ACTGGTAACA GGATTAGCAG AGCGAGGTAT GTAGGCGGTG CTACAGAGTT  CTTGAAGTGG TGGCCTAACT ACGGCTACAC TAGAAGAACA GTATTTGGTA  TCTGCGCTCT GCTGAAGCCA GTTACCTTCG GAAAAAGAGT TGGTAGCTCT  TGATCCGGCA AACAAACCAC CGCTGGTAGC GGTGGTTTTT TTGTTTGCAA  GCAGCAGATT ACGCGCAGAA AAAAAGGATC TCAAGAAGAT CCTTTGATCT  TTTCTACGGG GTCTGACGCT CAGTGGAACG AAAACTCACG TTAAGGGATT  TTGGTCATGA GATTATCAAA AAGGATCTTC ACCTAGATCC TTTTAAATTA  AAAATGAAGT TTTAAATCAA TCTAAAGTAT ATATGAGTAA ACTTGGTCTG  ACAGTTACCA ATGCTTAATC AGTGAGGCAC CTATCTCAGC GATCTGTCTA  TTTCGTTCAT CCATAGTTGC CTGACTCCCC GTCGTGTAGA TAACTACGAT  ACGGGAGGGC TTACCATCTG GCCCCAGTGC TGCAATGATA CCGCGAGACC  CACGCTCACC GGCTCCAGAT TTATCAGCAA TAAACCAGCC AGCCGGAAGG  GCCGAGCGCA GAAGTGGTCC TGCAACTTTA TCCGCCTCCA TCCAGTCTAT  TAATTGTTGC CGGGAAGCTA GAGTAAGTAG TTCGCCAGTT AATAGTTTGC  GCAACGTTGT TGCCATTGCT ACAGGCATCG TGGTGTCACG CTCGTCGTTT  GGTATGGCTT CATTCAGCTC CGGTTCCCAA CGATCAAGGC GAGTTACATG  ATCCCCCATG TTGTGCAAAA AAGCGGTTAG CTCCTTCGGT CCTCCGATCG  TTGTCAGAAG TAAGTTGGCC GCAGTGTTAT CACTCATGGT TATGGCAGCA  CTGCATAATT CTCTTACTGT CATGCCATCC GTAAGATGCT TTTCTGTGAC  TGGTGAGTAC TCAACCAAGT CATTCTGAGA ATAGTGTATG CGGCGACCGA  GTTGCTCTTG CCCGGCGTCA ATACGGGATA ATACCGCGCC ACATAGCAGA  ACTTTAAAAG TGCTCATCAT TGGAAAACGT TCTTCGGGGC GAAAACTCTC  AAGGATCTTA CCGCTGTTGA GATCCAGTTC GATGTAACCC ACTCGTGCAC  CCAACTGATC TTCAGCATCT TTTACTTTCA CCAGCGTTTC TGGGTGAGCA  AAAACAGGAA GGCAAAATGC CGCAAAAAAG GGAATAAGGG CGACACGGAA  ATGTTGAATA CTCATACTCT TCCTTTTTCA ATATTATTGA AGCATTTATC  AGGGTTATTG TCTCATGAGC GGATACATAT TTGAATGTAT TTAGAAAAAT  AAACAAATAG GGGTTCCGCG CACATTTCCC CGAAAAGTGC CACCTGACGC  GCCCTGTAGC GGCGCATTAA GCGCGGCGGG TGTGGTGGTT ACGCGCAGCG  TGACCGCTAC ACTTGCCAGC GCCCTAGCGC CCGCTCCTTT CGCTTTCTTC  CCTTCCTTTC TCGCCACGTT CGCCGGCTTT CCCCGTCAAG CTCTAAATCG  GGGGCTCCCT TTAGGGTTCC GATTTAGTGC TTTACGGCAC CTCGACCCCA  AAAAACTTGA TTAGGGTGAT GGTTCACGTA GTGGGCCATC GCCCTGATAG  ACGGTTTTTC GCCCTTTGAC GTTGGAGTCC ACGTTCTTTA ATAGTGGACT  CTTGTTCCAA ACTGGAACAA CACTCAACCC TATCTCGGTC TATTCTTTTG  ATTTATAAGG GATTTTGCCG ATTTCGGCCT ATTGGTTAAA AAATGAGCTG  ATTTAACAAA AATTTAACGC GAATTTTAAC AAAATATTAA CGCTTACAAT  TTGCCATTCG CCATTCAGGC TGCGCAACTG TTGGGAAGGG CGATCGGTGC  GGGCCTCTTC GCTATTACGC CAGCCCAAGC TACCATGATA AGTAAGTAAT  ATTAAGGTAC GGGAGGTACT TGGAGCGGCC GCAATAAAAT ATCTTTATTT  TCATTACATC TGTGTGTTGG TTTTTTGTGT GAATCGATAG TACTAACATA  CGCTCTCCAT CAAAACAAAA CGAAACAAAA CAAACTAGCA AAATAGGCTG  TCCCCAGTGC AAGTGCAGGT GCCAGAACAT TTCTCTATCG ATA |
| --- | --- |

**7. pcDNA3.1-EP300-6xHis plasmid map：**


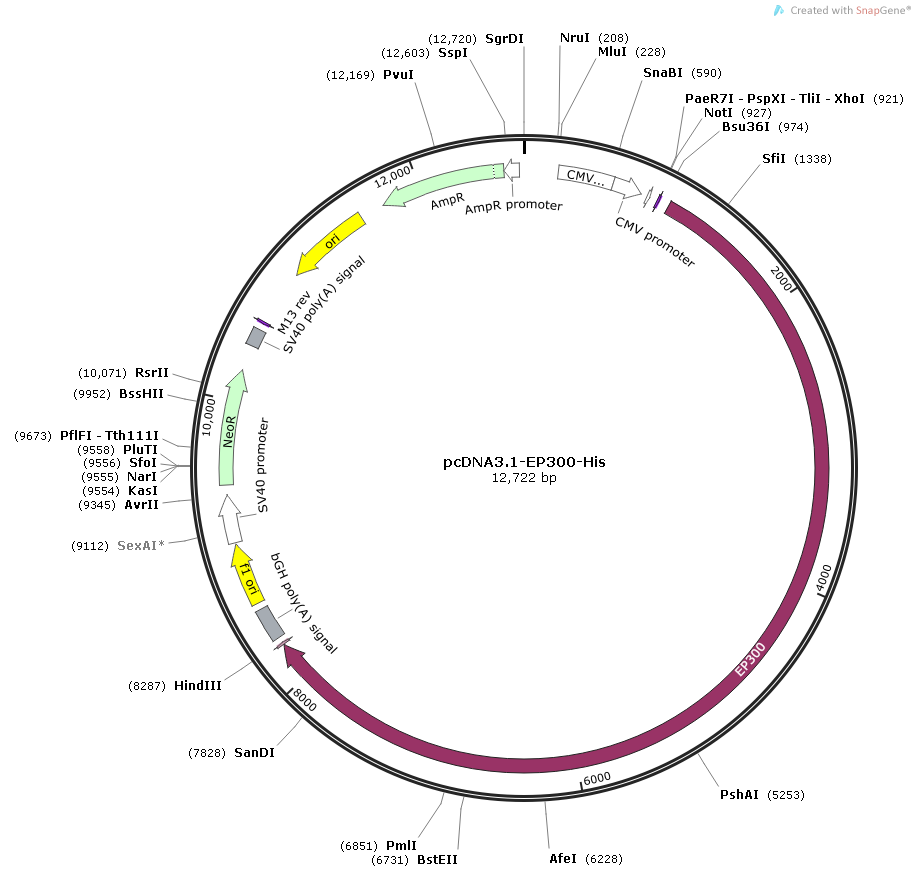


**Plasmid sequence (the red parts are P300 sequences):**

GACGGATCGGGAGATCTCCCGATCCCCTATGGTGCACTCTCAGTACAATCTGCTCTGATGCCGCATAGTTAAGCCAGTATCTGCTCCCTGCTTGTGTGTTGGAGGTCGCTGAGTAGTGCGCGAGCAAAATTTAAGCTACAACAAGGCAAGGCTTGACCGACAATTGCATGAAGAATCTGCTTAGGGTTAGGCGTTTTGCGCTGCTTCGCGATGTACGGGCCAGATATACGCGTTGACATTGATTATTGACTAGTTATTAATAGTAATCAATTACGGGGTCATTAGTTCATAGCCCATATATGGAGTTCCGCGTTACATAACTTACGGTAAATGGCCCGCCTGGCTGACCGCCCAACGACCCCCGCCCATTGACGTCAATAATGACGTATGTTCCCATAGTAACGCCAATAGGGACTTTCCATTGACGTCAATGGGTGGAGTATTTACGGTAAACTGCCCACTTGGCAGTACATCAAGTGTATCATATGCCAAGTACGCCCCCTATTGACGTCAATGACGGTAAATGGCCCGCCTGGCATTATGCCCAGTACATGACCTTATGGGACTTTCCTACTTGGCAGTACATCTACGTATTAGTCATCGCTATTACCATGGTGATGCGGTTTTGGCAGTACATCAATGGGCGTGGATAGCGGTTTGACTCACGGGGATTTCCAAGTCTCCACCCCATTGACGTCAATGGGAGTTTGTTTTGGCACCAAAATCAACGGGACTTTCCAAAATGTCGTAACAACTCCGCCCCATTGACGCAAATGGGCGGTAGGCGTGTACGGTGGGAGGTCTATATAAGCAGAGCTCTCTGGCTAACTAGAGAACCCACTGCTTACTGGCTTATCGAAATTAATACGACTCACTATAGGGAGACCCAAGCTGGCTAGCGTTTAAACGGGCCCTCTAGACTCGAGCGGCCGCTCTAGAACTAGTGGATCCCCCGGGCCGAAGAAGAGATTTCCTGAGGATTCTGGTTTTCCTCGCTTGTATCTCCGAAAGAATTAAAAATGGCCGAGAATGTGGTGGAACCGGGGCCGCCTTCAGCCAAGCGGCCTAAACTCTCATCTCCGGCCCTCTCGGCGTCCGCCAGCGATGGCACAGATTTTGGCTCTCTATTTGACTTGGAGCACGACTTACCAGATGAATTAATCAACTCTACAGAATTGGGACTAACCAATGGTGGTGATATTAATCAGCTTCAGACAAGTCTTGGCATGGTACAAGATGCAGCTTCTAAACATAAACAGCTGTCAGAATTGCTGCGATCTGGTAGTTCCCCTAACCTCAATATGGGAGTTGGTGGCCCAGGTCAAGTCATGGCCAGCCAGGCCCAACAGAGCAGTCCTGGATTAGGTTTGATAAATAGCATGGTCAAAAGCCCAATGACACAGGCAGGCTTGACTTCTCCCAACATGGGGATGGGCACTAGTGGACCAAATCAGGGTCCTACGCAGTCAACAGGTATGATGAACAGTCCAGTAAATCAGCCTGCCATGGGAATGAACACAGGGATGAATGCGGGCATGAATCCTGGAATGTTGGCTGCAGGCAATGGACAAGGGATAATGCCTAATCAAGTCATGAACGGTTCAATTGGAGCAGGCCGAGGGCGACAGAATATGCAGTACCCAAACCCAGGCATGGGAAGTGCTGGCAACTTACTGACTGAGCCTCTTCAGCAGGGCTCTCCCCAGATGGGAGGACAAACAGGATTGAGAGGCCCCCAGCCTCTTAAGATGGGAATGATGAACAACCCCAATCCTTATGGTTCACCATATACTCAGAATCCTGGACAGCAGATTGGAGCCAGTGGCCTTGGTCTCCAGATTCAGACAAAAACTGTACTATCAAATAACTTATCTCCATTTGCTATGGACAAAAAGGCAGTTCCTGGTGGAGGAATGCCCAACATGGGTCAACAGCCAGCCCCGCAGGTCCAGCAGCCAGGTCTGGTGACTCCAGTTGCCCAAGGGATGGGTTCTGGAGCACATACAGCTGATCCAGAGAAGCGCAAGCTCATCCAGCAGCAGCTTGTTCTCCTTTTGCATGCTCACAAGTGCCAGCGCCGGGAACAGGCCAATGGGGAAGTGAGGCAGTGCAACCTTCCCCACTGTCGCACAATGAAGAATGTCCTAAACCACATGACACACTGCCAGTCAGGCAAGTCTTGCCAAGTGGCACACTGTGCATCTTCTCGACAAATCATTTCACACTGGAAGAATTGTACAAGACATGATTGTCCTGTGTGTCTCCCCCTCAAAAATGCTGGTGATAAGAGAAATCAACAGCCAATTTTGACTGGAGCACCCGTTGGACTTGGAAATCCTAGCTCTCTAGGGGTGGGTCAACAGTCTGCCCCCAACCTAAGCACTGTTAGTCAGATTGATCCCAGCTCCATAGAAAGAGCCTATGCAGCTCTTGGACTACCCTATCAAGTAAATCAGATGCCGACACAACCCCAGGTGCAAGCAAAGAACCAGCAGAATCAGCAGCCTGGGCAGTCTCCCCAAGGCATGCGGCCCATGAGCAACATGAGTGCTAGTCCTATGGGAGTAAATGGAGGTGTAGGAGTTCAAACGCCGAGTCTTCTTTCTGACTCAATGTTGCATTCAGCCATAAATTCTCAAAACCCAATGATGAGTGAAAATGCCAGTGTGCCCTCCATGGGTCCTATGCCAACAGCAGCTCAACCATCCACTACTGGAATTCGGAAACAGTGGCACGAAGATATTACTCAGGATCTTCGAAATCATCTTGTTCACAAACTCGTCCAAGCCATATTTCCTACGCCGGATCCTGCTGCTTTAAAAGACAGACGGATGGAAAACCTAGTTGCATATGCTCGGAAAGTTGAAGGGGACATGTATGAATCTGCAAACAATCGAGCGGAATACTACCACCTTCTAGCTGAGAAAATCTATAAGATCCAGAAAGAACTAGAAGAAAAACGAAGGACCAGACTACAGAAGCAGAACATGCTACCAAATGCTGCAGGCATGGTTCCAGTTTCCATGAATCCAGGGCCTAACATGGGACAGCCGCAACCAGGAATGACTTCTAATGGCCCTCTACCTGACCCAAGTATGATCCGTGGCAGTGTGCCAAACCAGATGATGCCTCGAATAACTCCACAATCTGGTTTGAATCAATTTGGCCAGATGAGCATGGCCCAGCCCCCTATTGTACCCCGGCAAACCCCTCCTCTTCAGCACCATGGACAGTTGGCTCAACCTGGAGCTCTCAACCCGCCTATGGGCTATGGGCCTCGTATGCAACAGCCTTCCAACCAGGGCCAGTTCCTTCCTCAGACTCAGTTCCCATCACAGGGAATGAATGTAACAAATATCCCTTTGGCTCCGTCCAGCGGTCAAGCTCCAGTGTCTCAAGCACAAATGTCTAGTTCTTCCTGCCCGGTGAACTCTCCTATAATGCCTCCAGGGTCTCAGGGGAGCCACATTCACTGTCCCCAGCTTCCTCAACCAGCTCTTCATCAGAATTCACCCTCGCCTGTACCTAGTCGTACCCCCACCCCTCACCATACTCCCCCAAGCATAGGGGCTCAGCAGCCACCAGCAACAACAATTCCAGCCCCTGTTCCTACACCTCCTGCCATGCCACCTGGGCCACAGTCCCAGGCTCTACATCCCCCTCCAAGGCAGACACCTACACCACCAACAACACAACTTCCCCAACAAGTGCAGCCTTCACTTCCTGCTGCACCTTCTGCTGACCAGCCCCAGCAGCAGCCTCGCTCACAGCAGAGCACAGCAGCGTCTGTTCCTACCCCAACAGCACCGCTGCTTCCTCCGCAGCCTGCAACTCCACTTTCCCAGCCAGCTGTAAGCATTGAAGGACAGGTATCAAATCCTCCATCTACTAGTAGCACAGAAGTGAATTCTCAGGCCATTGCTGAGAAGCAGCCTTCCCAGGAAGTGAAGATGGAGGCCAAAATGGAAGTGGATCAACCAGAACCAGCAGATACTCAGCCGGAGGATATTTCAGAGTCTAAAGTGGAAGACTGTAAAATGGAATCTACCGAAACAGAAGAGAGAAGCACTGAGTTAAAAACTGAAATAAAAGAGGAGGAAGACCAGCCAAGTACTTCAGCTACCCAGTCATCTCCGGCTCCAGGACAGTCAAAGAAAAAGATTTTCAAACCAGAAGAACTACGACAGGCACTGATGCCAACTTTGGAGGCACTTTACCGTCAGGATCCAGAATCCCTTCCCTTTCGTCAACCTGTGGACCCTCAGCTTTTAGGAATCCCTGATTACTTTGATATTGTGAAGAGCCCCATGGATCTTTCTACCATTAAGAGGAAGTTAGACACTGGACAGTATCAGGAGCCCTGGCAGTATGTCGATGATATTTGGCTTATGTTCAATAATGCCTGGTTATATAACCGGAAAACATCACGGGTATACAAATACTGCTCCAAGCTCTCTGAGGTCTTTGAACAAGAAATTGACCCAGTGATGCAAAGCCTTGGATACTGTTGTGGCAGAAAGTTGGAGTTCTCTCCACAGACACTGTGTTGCTACGGCAAACAGTTGTGCACAATACCTCGTGATGCCACTTATTACAGTTACCAGAACAGGTATCATTTCTGTGAGAAGTGTTTCAATGAGATCCAAGGGGAGAGCGTTTCTTTGGGGGATGACCCTTCCCAGCCTCAAACTACAATAAATAAAGAACAATTTTCCAAGAGAAAAAATGACACACTGGATCCTGAACTGTTTGTTGAATGTACAGAGTGCGGAAGAAAGATGCATCAGATCTGTGTCCTTCACCATGAGATCATCTGGCCTGCTGGATTCGTCTGTGATGGCTGTTTAAAGAAAAGTGCACGAACTAGGAAAGAAAATAAGTTTTCTGCTAAAAGGTTGCCATCTACCAGACTTGGCACCTTTCTAGAGAATCGTGTGAATGACTTTCTGAGGCGACAGAATCACCCTGAGTCAGGAGAGGTCACTGTTAGAGTAGTTCATGCTTCTGACAAAACCGTGGAAGTAAAACCAGGCATGAAAGCAAGGTTTGTGGACAGTGGAGAGATGGCAGAATCCTTTCCATACCGAACCAAAGCCCTCTTTGCCTTTGAAGAAATTGATGGTGTTGACCTGTGCTTCTTTGGCATGCATGTTCAAGAGTATGGCTCTGACTGCCCTCCACCCAACCAGAGGAGAGTATACATATCTTACCTCGATAGTGTTCATTTCTTCCGTCCTAAATGCTTGAGGACTGCAGTCTATCATGAAATCCTAATTGGATATTTAGAATATGTCAAGAAATTAGGTTACACAACAGGGCATATTTGGGCATGTCCACCAAGTGAGGGAGATGATTATATCTTCCATTGCCATCCTCCTGACCAGAAGATACCCAAGCCCAAGCGACTGCAGGAATGGTACAAAAAAATGCTTGACAAGGCTGTATCAGAGCGTATTGTCCATGACTACAAGGATATTTTTAAACAAGCTACTGAAGATAGATTAACAAGTGCAAAGGAATTGCCTTATTTCGAGGGTGATTTCTGGCCCAATGTTCTGGAAGAAAGCATTAAGGAACTGGAACAGGAGGAAGAAGAGAGAAAACGAGAGGAAAACACCAGCAATGAAAGCACAGATGTGACCAAGGGAGACAGCAAAAATGCTAAAAAGAAGAATAATAAGAAAACCAGCAAAAATAAGAGCAGCCTGAGTAGGGGCAACAAGAAGAAACCCGGGATGCCCAATGTATCTAACGACCTCTCACAGAAACTATATGCCACCATGGAGAAGCATAAAGAGGTCTTCTTTGTGATCCGCCTCATTGCTGGCCCTGCTGCCAACTCCCTGCCTCCCATTGTTGATCCTGATCCTCTCATCCCCTGCGATCTGATGGATGGTCGGGATGCGTTTCTCACGCTGGCAAGGGACAAGCACCTGGAGTTCTCTTCACTCCGAAGAGCCCAGTGGTCCACCATGTGCATGCTGGTGGAGCTGCACACGCAGAGCCAGGACCGCTTTGTCTACACCTGCAATGAATGCAAGCACCATGTGGAGACACGCTGGCACTGTACTGTCTGTGAGGATTATGACTTGTGTATCACCTGCTATAACACTAAAAACCATGACCACAAAATGGAGAAACTAGGCCTTGGCTTAGATGATGAGAGCAACAACCAGCAGGCTGCAGCCACCCAGAGCCCAGGCGATTCTCGCCGCCTGAGTATCCAGCGCTGCATCCAGTCTCTGGTCCATGCTTGCCAGTGTCGGAATGCCAATTGCTCACTGCCATCCTGCCAGAAGATGAAGCGGGTTGTGCAGCATACCAAGGGTTGCAAACGGAAAACCAATGGCGGGTGCCCCATCTGCAAGCAGCTCATTGCCCTCTGCTGCTACCATGCCAAGCACTGCCAGGAGAACAAATGCCCGGTGCCGTTCTGCCTAAACATCAAGCAGAAGCTCCGGCAGCAACAGCTGCAGCACCGACTACAGCAGGCCCAAATGCTTCGCAGGAGGATGGCCAGCATGCAGCGGACTGGTGTGGTTGGGCAGCAACAGGGCCTCCCTTCCCCCACTCCTGCCACTCCAACGACACCAACTGGCCAACAGCCAACCACCCCGCAGACGCCCCAGCCCACTTCTCAGCCTCAGCCTACCCCTCCCAATAGCATGCCACCCTACTTGCCCAGGACTCAAGCTGCTGGCCCTGTGTCCCAGGGTAAGGCAGCAGGCCAGGTGACCCCTCCAACCCCTCCTCAGACTGCTCAGCCACCCCTTCCAGGGCCCCCACCTGCAGCAGTGGAAATGGCAATGCAGATTCAGAGAGCAGCGGAGACGCAGCGCCAGATGGCCCACGTGCAAATTTTTCAAAGGCCAATCCAACACCAGATGCCCCCGATGACTCCCATGGCCCCCATGGGTATGAACCCACCTCCCATGACCAGAGGTCCCAGTGGGCATTTGGAGCCAGGGATGGGACCGACAGGGATGCAGCAACAGCCACCCTGGAGCCAAGGAGGATTGCCTCAGCCCCAGCAACTACAGTCTGGGATGCCAAGGCCAGCCATGATGTCAGTGGCCCAGCATGGTCAACCTTTGAACATGGCTCCACAACCAGGATTGGGCCAGGTAGGTATCAGCCCACTCAAACCAGGCACTGTGTCTCAACAAGCCTTACAAAACCTTTTGCGGACTCTCAGGTCTCCCAGCTCTCCCCTGCAGCAGCAACAGGTGCTTAGTATCCTTCACGCCAACCCCCAGCTGTTGGCTGCATTCATCAAGCAGCGGGCTGCCAAGTATGCCAACTCTAATCCACAACCCATCCCTGGGCAGCCTGGCATGCCCCAGGGGCAGCCAGGGCTACAGCCACCTACCATGCCAGGTCAGCAGGGGGTCCACTCCAATCCAGCCATGCAGAACATGAATCCAATGCAGGCGGGCGTTCAGAGGGCTGGCCTGCCCCAGCAGCAACCACAGCAGCAACTCCAGCCACCCATGGGAGGGATGAGCCCCCAGGCTCAGCAGATGAACATGAACCACAACACCATGCCTTCACAATTCCGAGACATCTTGAGACGACAGCAAATGATGCAACAGCAGCAGCAACAGGGAGCAGGGCCAGGAATAGGCCCTGGAATGGCCAACCATAACCAGTTCCAGCAACCCCAAGGAGTTGGCTACCCACCACAGCAGCAGCAGCGGATGCAGCATCACATGCAACAGATGCAACAAGGAAATATGGGACAGATAGGCCAGCTTCCCCAGGCCTTGGGAGCAGAGGCAGGTGCCAGTCTACAGGCCTATCAGCAGCGACTCCTTCAGCAACAGATGGGGTCCCCTGTTCAGCCCAACCCCATGAGCCCCCAGCAGCATATGCTCCCAAATCAGGCCCAGTCCCCACACCTACAAGGCCAGCAGATCCCTAATTCTCTCTCCAATCAAGTGCGCTCTCCCCAGCCTGTCCCTTCTCCACGGCCACAGTCCCAGCCCCCCCACTCCAGTCCTTCCCCAAGGATGCAGCCTCAGCCTTCTCCACACCACGTTTCCCCACAGACAAGTTCCCCACATCCTGGACTGGTAGCTGCCCAGGCCAACCCCATGGAACAAGGGCATTTTGCCAGCCCGGACCAGAATTCAATGCTTTCTCAGCTTGCTAGCAATCCAGGCATGGCAAACCTCCATGGTGCAAGCGCCACGGACCTGGGACTCAGCACCGATAACTCAGACTTGAATTCAAACCTCTCACAGAGTACACTAGACATACACCACCATCACCACCATTAGTGATACTAAGCTTAAGTTTAAACCGCTGATCAGCCTCGACTGTGCCTTCTAGTTGCCAGCCATCTGTTGTTTGCCCCTCCCCCGTGCCTTCCTTGACCCTGGAAGGTGCCACTCCCACTGTCCTTTCCTAATAAAATGAGGAAATTGCATCGCATTGTCTGAGTAGGTGTCATTCTATTCTGGGGGGTGGGGTGGGGCAGGACAGCAAGGGGGAGGATTGGGAAGACAATAGCAGGCATGCTGGGGATGCGGTGGGCTCTATGGCTTCTGAGGCGGAAAGAACCAGCTGGGGCTCTAGGGGGTATCCCCACGCGCCCTGTAGCGGCGCATTAAGCGCGGCGGGTGTGGTGGTTACGCGCAGCGTGACCGCTACACTTGCCAGCGCCCTAGCGCCCGCTCCTTTCGCTTTCTTCCCTTCCTTTCTCGCCACGTTCGCCGGCTTTCCCCGTCAAGCTCTAAATCGGGGGCTCCCTTTAGGGTTCCGATTTAGTGCTTTACGGCACCTCGACCCCAAAAAACTTGATTAGGGTGATGGTTCACGTAGTGGGCCATCGCCCTGATAGACGGTTTTTCGCCCTTTGACGTTGGAGTCCACGTTCTTTAATAGTGGACTCTTGTTCCAAACTGGAACAACACTCAACCCTATCTCGGTCTATTCTTTTGATTTATAAGGGATTTTGCCGATTTCGGCCTATTGGTTAAAAAATGAGCTGATTTAACAAAAATTTAACGCGAATTAATTCTGTGGAATGTGTGTCAGTTAGGGTGTGGAAAGTCCCCAGGCTCCCCAGCAGGCAGAAGTATGCAAAGCATGCATCTCAATTAGTCAGCAACCAGGTGTGGAAAGTCCCCAGGCTCCCCAGCAGGCAGAAGTATGCAAAGCATGCATCTCAATTAGTCAGCAACCATAGTCCCGCCCCTAACTCCGCCCATCCCGCCCCTAACTCCGCCCAGTTCCGCCCATTCTCCGCCCCATGGCTGACTAATTTTTTTTATTTATGCAGAGGCCGAGGCCGCCTCTGCCTCTGAGCTATTCCAGAAGTAGTGAGGAGGCTTTTTTGGAGGCCTAGGCTTTTGCAAAAAGCTCCCGGGAGCTTGTATATCCATTTTCGGATCTGATCAAGAGACAGGATGAGGATCGTTTCGCATGATTGAACAAGATGGATTGCACGCAGGTTCTCCGGCCGCTTGGGTGGAGAGGCTATTCGGCTATGACTGGGCACAACAGACAATCGGCTGCTCTGATGCCGCCGTGTTCCGGCTGTCAGCGCAGGGGCGCCCGGTTCTTTTTGTCAAGACCGACCTGTCCGGTGCCCTGAATGAACTGCAGGACGAGGCAGCGCGGCTATCGTGGCTGGCCACGACGGGCGTTCCTTGCGCAGCTGTGCTCGACGTTGTCACTGAAGCGGGAAGGGACTGGCTGCTATTGGGCGAAGTGCCGGGGCAGGATCTCCTGTCATCTCACCTTGCTCCTGCCGAGAAAGTATCCATCATGGCTGATGCAATGCGGCGGCTGCATACGCTTGATCCGGCTACCTGCCCATTCGACCACCAAGCGAAACATCGCATCGAGCGAGCACGTACTCGGATGGAAGCCGGTCTTGTCGATCAGGATGATCTGGACGAAGAGCATCAGGGGCTCGCGCCAGCCGAACTGTTCGCCAGGCTCAAGGCGCGCATGCCCGACGGCGAGGATCTCGTCGTGACCCATGGCGATGCCTGCTTGCCGAATATCATGGTGGAAAATGGCCGCTTTTCTGGATTCATCGACTGTGGCCGGCTGGGTGTGGCGGACCGCTATCAGGACATAGCGTTGGCTACCCGTGATATTGCTGAAGAGCTTGGCGGCGAATGGGCTGACCGCTTCCTCGTGCTTTACGGTATCGCCGCTCCCGATTCGCAGCGCATCGCCTTCTATCGCCTTCTTGACGAGTTCTTCTGAGCGGGACTCTGGGGTTCGAAATGACCGACCAAGCGACGCCCAACCTGCCATCACGAGATTTCGATTCCACCGCCGCCTTCTATGAAAGGTTGGGCTTCGGAATCGTTTTCCGGGACGCCGGCTGGATGATCCTCCAGCGCGGGGATCTCATGCTGGAGTTCTTCGCCCACCCCAACTTGTTTATTGCAGCTTATAATGGTTACAAATAAAGCAATAGCATCACAAATTTCACAAATAAAGCATTTTTTTCACTGCATTCTAGTTGTGGTTTGTCCAAACTCATCAATGTATCTTATCATGTCTGTATACCGTCGACCTCTAGCTAGAGCTTGGCGTAATCATGGTCATAGCTGTTTCCTGTGTGAAATTGTTATCCGCTCACAATTCCACACAACATACGAGCCGGAAGCATAAAGTGTAAAGCCTGGGGTGCCTAATGAGTGAGCTAACTCACATTAATTGCGTTGCGCTCACTGCCCGCTTTCCAGTCGGGAAACCTGTCGTGCCAGCTGCATTAATGAATCGGCCAACGCGCGGGGAGAGGCGGTTTGCGTATTGGGCGCTCTTCCGCTTCCTCGCTCACTGACTCGCTGCGCTCGGTCGTTCGGCTGCGGCGAGCGGTATCAGCTCACTCAAAGGCGGTAATACGGTTATCCACAGAATCAGGGGATAACGCAGGAAAGAACATGTGAGCAAAAGGCCAGCAAAAGGCCAGGAACCGTAAAAAGGCCGCGTTGCTGGCGTTTTTCCATAGGCTCCGCCCCCCTGACGAGCATCACAAAAATCGACGCTCAAGTCAGAGGTGGCGAAACCCGACAGGACTATAAAGATACCAGGCGTTTCCCCCTGGAAGCTCCCTCGTGCGCTCTCCTGTTCCGACCCTGCCGCTTACCGGATACCTGTCCGCCTTTCTCCCTTCGGGAAGCGTGGCGCTTTCTCATAGCTCACGCTGTAGGTATCTCAGTTCGGTGTAGGTCGTTCGCTCCAAGCTGGGCTGTGTGCACGAACCCCCCGTTCAGCCCGACCGCTGCGCCTTATCCGGTAACTATCGTCTTGAGTCCAACCCGGTAAGACACGACTTATCGCCACTGGCAGCAGCCACTGGTAACAGGATTAGCAGAGCGAGGTATGTAGGCGGTGCTACAGAGTTCTTGAAGTGGTGGCCTAACTACGGCTACACTAGAAGAACAGTATTTGGTATCTGCGCTCTGCTGAAGCCAGTTACCTTCGGAAAAAGAGTTGGTAGCTCTTGATCCGGCAAACAAACCACCGCTGGTAGCGGTGGTTTTTTTGTTTGCAAGCAGCAGATTACGCGCAGAAAAAAAGGATCTCAAGAAGATCCTTTGATCTTTTCTACGGGGTCTGACGCTCAGTGGAACGAAAACTCACGTTAAGGGATTTTGGTCATGAGATTATCAAAAAGGATCTTCACCTAGATCCTTTTAAATTAAAAATGAAGTTTTAAATCAATCTAAAGTATATATGAGTAAACTTGGTCTGACAGTTACCAATGCTTAATCAGTGAGGCACCTATCTCAGCGATCTGTCTATTTCGTTCATCCATAGTTGCCTGACTCCCCGTCGTGTAGATAACTACGATACGGGAGGGCTTACCATCTGGCCCCAGTGCTGCAATGATACCGCGAGACCCACGCTCACCGGCTCCAGATTTATCAGCAATAAACCAGCCAGCCGGAAGGGCCGAGCGCAGAAGTGGTCCTGCAACTTTATCCGCCTCCATCCAGTCTATTAATTGTTGCCGGGAAGCTAGAGTAAGTAGTTCGCCAGTTAATAGTTTGCGCAACGTTGTTGCCATTGCTACAGGCATCGTGGTGTCACGCTCGTCGTTTGGTATGGCTTCATTCAGCTCCGGTTCCCAACGATCAAGGCGAGTTACATGATCCCCCATGTTGTGCAAAAAAGCGGTTAGCTCCTTCGGTCCTCCGATCGTTGTCAGAAGTAAGTTGGCCGCAGTGTTATCACTCATGGTTATGGCAGCACTGCATAATTCTCTTACTGTCATGCCATCCGTAAGATGCTTTTCTGTGACTGGTGAGTACTCAACCAAGTCATTCTGAGAATAGTGTATGCGGCGACCGAGTTGCTCTTGCCCGGCGTCAATACGGGATAATACCGCGCCACATAGCAGAACTTTAAAAGTGCTCATCATTGGAAAACGTTCTTCGGGGCGAAAACTCTCAAGGATCTTACCGCTGTTGAGATCCAGTTCGATGTAACCCACTCGTGCACCCAACTGATCTTCAGCATCTTTTACTTTCACCAGCGTTTCTGGGTGAGCAAAAACAGGAAGGCAAAATGCCGCAAAAAAGGGAATAAGGGCGACACGGAAATGTTGAATACTCATACTCTTCCTTTTTCAATATTATTGAAGCATTTATCAGGGTTATTGTCTCATGAGCGGATACATATTTGAATGTATTTAGAAAAATAAACAAATAGGGGTTCCGCGCACATTTCCCCGAAAAGTGCCACCTGACGTC

**8. ELK1 pcDNA3.1-3xFlag-C plasmid map:**


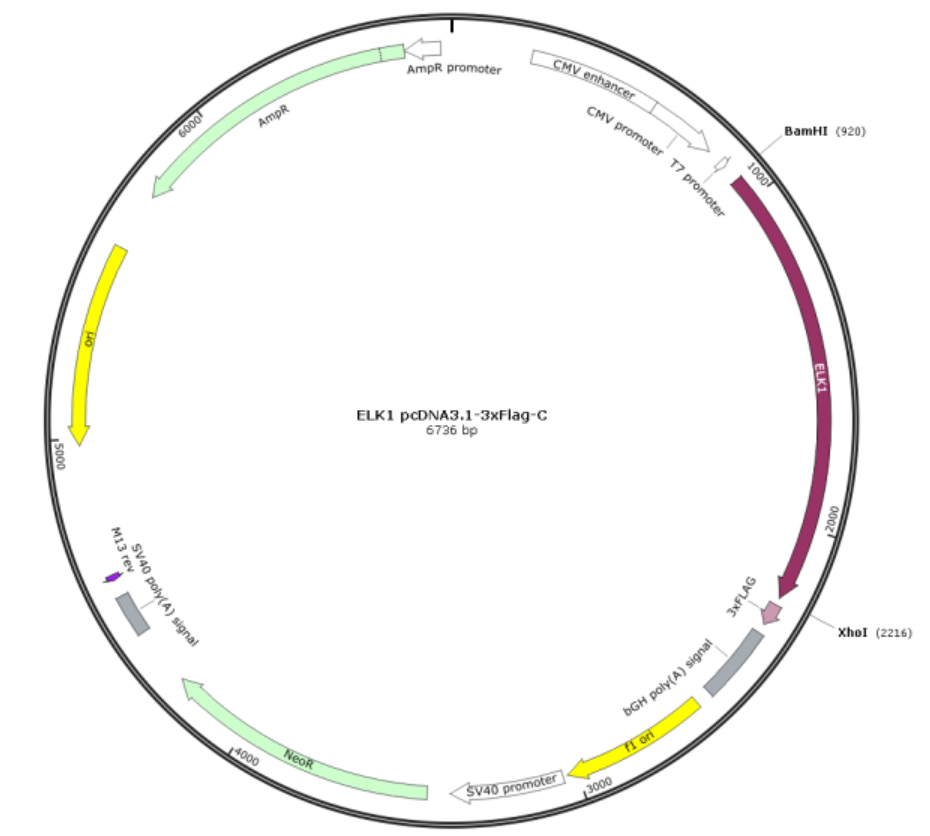


**Plasmid sequence (the red parts are ELK1 sequences):**

GACGGATCGGGAGATCTCCCGATCCCCTATGGTGCACTCTCAGTACAATCTGCTCTGATGCCGCATAGTTAAGCCAGTATCTGCTCCCTGCTTGTGTGTTGGAGGTCGCTGAGTAGTGCGCGAGCAAAATTTAAGCTACAACAAGGCAAGGCTTGACCGACAATTGCATGAAGAATCTGCTTAGGGTTAGGCGTTTTGCGCTGCTTCGCGATGTACGGGCCAGATATACGCGTTGACATTGATTATTGACTAGTTATTAATAGTAATCAATTACGGGGTCATTAGTTCATAGCCCATATATGGAGTTCCGCGTTACATAACTTACGGTAAATGGCCCGCCTGGCTGACCGCCCAACGACCCCCGCCCATTGACGTCAATAATGACGTATGTTCCCATAGTAACGCCAATAGGGACTTTCCATTGACGTCAATGGGTGGAGTATTTACGGTAAACTGCCCACTTGGCAGTACATCAAGTGTATCATATGCCAAGTACGCCCCCTATTGACGTCAATGACGGTAAATGGCCCGCCTGGCATTATGCCCAGTACATGACCTTATGGGACTTTCCTACTTGGCAGTACATCTACGTATTAGTCATCGCTATTACCATGGTGATGCGGTTTTGGCAGTACATCAATGGGCGTGGATAGCGGTTTGACTCACGGGGATTTCCAAGTCTCCACCCCATTGACGTCAATGGGAGTTTGTTTTGGCACCAAAATCAACGGGACTTTCCAAAATGTCGTAACAACTCCGCCCCATTGACGCAAATGGGCGGTAGGCGTGTACGGTGGGAGGTCTATATAAGCAGAGCTCTCTGGCTAACTAGAGAACCCACTGCTTACTGGCTTATCGAAATTAATACGACTCACTATAGGGAGACCCAAGCTGGCTAGTTAAGCTTGGTACCGAGCTCGGATCCGCCACCATGGACCCATCTGTGACGCTGTGGCAGTTTCTGCTGCAGCTGCTGAGAGAGCAAGGCAATGGCCACATCATCTCCTGGACTTCACGGGATGGTGGTGAATTCAAGCTGGTGGATGCAGAGGAGGTGGCCCGGCTGTGGGGGCTACGCAAGAACAAGACCAACATGAATTACGACAAGCTCAGCCGGGCCTTGCGGTACTACTATGACAAGAACATCATCCGCAAGGTGAGCGGCCAGAAGTTCGTCTACAAGTTTGTGTCCTACCCTGAGGTCGCAGGGTGCTCCACTGAGGACTGCCCGCCCCAGCCAGAGGTGTCTGTTACCTCCACCATGCCAAATGTGGCCCCTGCTGCTATACATGCCGCCCCAGGGGACACTGTCTCTGGAAAGCCAGGCACACCCAAGGGTGCAGGAATGGCAGGCCCAGGCGGTTTGGCACGCAGCAGCCGGAACGAGTACATGCGCTCGGGCCTCTATTCCACCTTCACCATCCAGTCTCTGCAGCCGCAGCCACCCCCTCATCCTCGGCCTGCTGTGGTGCTCCCCAGTGCAGCTCCTGCAGGGGCAGCAGCGCCCCCCTCGGGGAGCAGGAGCACCAGTCCAAGCCCCTTGGAGGCCTGTCTGGAGGCTGAAGAGGCCGGCTTGCCTCTGCAGGTCATCCTGACCCCGCCCGAGGCCCCAAACCTGAAATCGGAAGAGCTTAATGTGGAGCCGGGTTTGGGCCGGGCTTTGCCCCCAGAAGTGAAAGTAGAAGGGCCCAAGGAAGAGTTGGAAGTTGCGGGGGAGAGAGGGTTTGTGCCAGAAACCACCAAGGCCGAGCCAGAAGTCCCTCCACAGGAGGGCGTGCCAGCCCGGCTGCCCGCGGTTGTTATGGACACCGCAGGGCAGGCGGGCGGCCATGCGGCTTCCAGCCCTGAGATCTCCCAGCCGCAGAAGGGCCGGAAGCCCCGGGACCTAGAGCTTCCACTCAGCCCGAGCCTGCTAGGTGGGCCGGGACCCGAACGGACCCCAGGATCGGGAAGTGGCTCCGGCCTCCAGGCTCCGGGGCCGGCGCTGACCCCATCCCTGCTTCCTACGCATACATTGACCCCGGTGCTGCTGACACCCAGCTCGCTGCCTCCTAGCATTCACTTCTGGAGCACCCTGAGTCCCATTGCGCCCCGTAGCCCGGCCAAGCTCTCCTTCCAGTTTCCATCCAGTGGCAGCGCCCAGGTGCACATCCCTTCTATCAGCGTGGATGGCCTCTCGACCCCCGTGGTGCTCTCCCCAGGGCCCCAGAAGCCACTCGAGTCTAGAGGGCCCTTCGACTACAAAGACCATGACGGTGATTATAAAGATCATGACATCGACTACAAGGATGACGATGACAAGTGAGTTTAAACCCGCTGATCAGCCTCGACTGTGCCTTCTAGTTGCCAGCCATCTGTTGTTTGCCCCTCCCCCGTGCCTTCCTTGACCCTGGAAGGTGCCACTCCCACTGTCCTTTCCTAATAAAATGAGGAAATTGCATCGCATTGTCTGAGTAGGTGTCATTCTATTCTGGGGGGTGGGGTGGGGCAGGACAGCAAGGGGGAGGATTGGGAAGACAATAGCAGGCATGCTGGGGATGCGGTGGGCTCTATGGCTTCTGAGGCGGAAAGAACCAGCTGGGGCTCTAGGGGGTATCCCCACGCGCCCTGTAGCGGCGCATTAAGCGCGGCGGGTGTGGTGGTTACGCGCAGCGTGACCGCTACACTTGCCAGCGCCCTAGCGCCCGCTCCTTTCGCTTTCTTCCCTTCCTTTCTCGCCACGTTCGCCGGCTTTCCCCGTCAAGCTCTAAATCGGGGGCTCCCTTTAGGGTTCCGATTTAGTGCTTTACGGCACCTCGACCCCAAAAAACTTGATTAGGGTGATGGTTCACGTAGTGGGCCATCGCCCTGATAGACGGTTTTTCGCCCTTTGACGTTGGAGTCCACGTTCTTTAATAGTGGACTCTTGTTCCAAACTGGAACAACACTCAACCCTATCTCGGTCTATTCTTTTGATTTATAAGGGATTTTGCCGATTTCGGCCTATTGGTTAAAAAATGAGCTGATTTAACAAAAATTTAACGCGAATTAATTCTGTGGAATGTGTGTCAGTTAGGGTGTGGAAAGTCCCCAGGCTCCCCAGCAGGCAGAAGTATGCAAAGCATGCATCTCAATTAGTCAGCAACCAGGTGTGGAAAGTCCCCAGGCTCCCCAGCAGGCAGAAGTATGCAAAGCATGCATCTCAATTAGTCAGCAACCATAGTCCCGCCCCTAACTCCGCCCATCCCGCCCCTAACTCCGCCCAGTTCCGCCCATTCTCCGCCCCATGGCTGACTAATTTTTTTTATTTATGCAGAGGCCGAGGCCGCCTCTGCCTCTGAGCTATTCCAGAAGTAGTGAGGAGGCTTTTTTGGAGGCCTAGGCTTTTGCAAAAAGCTCCCGGGAGCTTGTATATCCATTTTCGGATCTGATCAAGAGACAGGATGAGGATCGTTTCGCATGATTGAACAAGATGGATTGCACGCAGGTTCTCCGGCCGCTTGGGTGGAGAGGCTATTCGGCTATGACTGGGCACAACAGACAATCGGCTGCTCTGATGCCGCCGTGTTCCGGCTGTCAGCGCAGGGGCGCCCGGTTCTTTTTGTCAAGACCGACCTGTCCGGTGCCCTGAATGAACTGCAGGACGAGGCAGCGCGGCTATCGTGGCTGGCCACGACGGGCGTTCCTTGCGCAGCTGTGCTCGACGTTGTCACTGAAGCGGGAAGGGACTGGCTGCTATTGGGCGAAGTGCCGGGGCAGGATCTCCTGTCATCTCACCTTGCTCCTGCCGAGAAAGTATCCATCATGGCTGATGCAATGCGGCGGCTGCATACGCTTGATCCGGCTACCTGCCCATTCGACCACCAAGCGAAACATCGCATCGAGCGAGCACGTACTCGGATGGAAGCCGGTCTTGTCGATCAGGATGATCTGGACGAAGAGCATCAGGGGCTCGCGCCAGCCGAACTGTTCGCCAGGCTCAAGGCGCGCATGCCCGACGGCGAGGATCTCGTCGTGACCCATGGCGATGCCTGCTTGCCGAATATCATGGTGGAAAATGGCCGCTTTTCTGGATTCATCGACTGTGGCCGGCTGGGTGTGGCGGACCGCTATCAGGACATAGCGTTGGCTACCCGTGATATTGCTGAAGAGCTTGGCGGCGAATGGGCTGACCGCTTCCTCGTGCTTTACGGTATCGCCGCTCCCGATTCGCAGCGCATCGCCTTCTATCGCCTTCTTGACGAGTTCTTCTGAGCGGGACTCTGGGGTTCGCGAAATGACCGACCAAGCGACGCCCAACCTGCCATCACGAGATTTCGATTCCACCGCCGCCTTCTATGAAAGGTTGGGCTTCGGAATCGTTTTCCGGGACGCCGGCTGGATGATCCTCCAGCGCGGGGATCTCATGCTGGAGTTCTTCGCCCACCCCAACTTGTTTATTGCAGCTTATAATGGTTACAAATAAAGCAATAGCATCACAAATTTCACAAATAAAGCATTTTTTTCACTGCATTCTAGTTGTGGTTTGTCCAAACTCATCAATGTATCTTATCATGTCTGTATACCGTCGACCTCTAGCTAGAGCTTGGCGTAATCATGGTCATAGCTGTTTCCTGTGTGAAATTGTTATCCGCTCACAATTCCACACAACATACGAGCCGGAAGCATAAAGTGTAAAGCCTGGGGTGCCTAATGAGTGAGCTAACTCACATTAATTGCGTTGCGCTCACTGCCCGCTTTCCAGTCGGGAAACCTGTCGTGCCAGCTGCATTAATGAATCGGCCAACGCGCGGGGAGAGGCGGTTTGCGTATTGGGCGCTCTTCCGCTTCCTCGCTCACTGACTCGCTGCGCTCGGTCGTTCGGCTGCGGCGAGCGGTATCAGCTCACTCAAAGGCGGTAATACGGTTATCCACAGAATCAGGGGATAACGCAGGAAAGAACATGTGAGCAAAAGGCCAGCAAAAGGCCAGGAACCGTAAAAAGGCCGCGTTGCTGGCGTTTTTCCATAGGCTCCGCCCCCCTGACGAGCATCACAAAAATCGACGCTCAAGTCAGAGGTGGCGAAACCCGACAGGACTATAAAGATACCAGGCGTTTCCCCCTGGAAGCTCCCTCGTGCGCTCTCCTGTTCCGACCCTGCCGCTTACCGGATACCTGTCCGCCTTTCTCCCTTCGGGAAGCGTGGCGCTTTCTCATAGCTCACGCTGTAGGTATCTCAGTTCGGTGTAGGTCGTTCGCTCCAAGCTGGGCTGTGTGCACGAACCCCCCGTTCAGCCCGACCGCTGCGCCTTATCCGGTAACTATCGTCTTGAGTCCAACCCGGTAAGACACGACTTATCGCCACTGGCAGCAGCCACTGGTAACAGGATTAGCAGAGCGAGGTATGTAGGCGGTGCTACAGAGTTCTTGAAGTGGTGGCCTAACTACGGCTACACTAGAAGAACAGTATTTGGTATCTGCGCTCTGCTGAAGCCAGTTACCTTCGGAAAAAGAGTTGGTAGCTCTTGATCCGGCAAACAAACCACCGCTGGTAGCGGTGGTTTTTTTGTTTGCAAGCAGCAGATTACGCGCAGAAAAAAAGGATCTCAAGAAGATCCTTTGATCTTTTCTACGGGGTCTGACGCTCAGTGGAACGAAAACTCACGTTAAGGGATTTTGGTCATGAGATTATCAAAAAGGATCTTCACCTAGATCCTTTTAAATTAAAAATGAAGTTTTAAATCAATCTAAAGTATATATGAGTAAACTTGGTCTGACAGTTACCAATGCTTAATCAGTGAGGCACCTATCTCAGCGATCTGTCTATTTCGTTCATCCATAGTTGCCTGACTCCCCGTCGTGTAGATAACTACGATACGGGAGGGCTTACCATCTGGCCCCAGTGCTGCAATGATACCGCGAGACCCACGCTCACCGGCTCCAGATTTATCAGCAATAAACCAGCCAGCCGGAAGGGCCGAGCGCAGAAGTGGTCCTGCAACTTTATCCGCCTCCATCCAGTCTATTAATTGTTGCCGGGAAGCTAGAGTAAGTAGTTCGCCAGTTAATAGTTTGCGCAACGTTGTTGCCATTGCTACAGGCATCGTGGTGTCACGCTCGTCGTTTGGTATGGCTTCATTCAGCTCCGGTTCCCAACGATCAAGGCGAGTTACATGATCCCCCATGTTGTGCAAAAAAGCGGTTAGCTCCTTCGGTCCTCCGATCGTTGTCAGAAGTAAGTTGGCCGCAGTGTTATCACTCATGGTTATGGCAGCACTGCATAATTCTCTTACTGTCATGCCATCCGTAAGATGCTTTTCTGTGACTGGTGAGTACTCAACCAAGTCATTCTGAGAATAGTGTATGCGGCGACCGAGTTGCTCTTGCCCGGCGTCAATACGGGATAATACCGCGCCACATAGCAGAACTTTAAAAGTGCTCATCATTGGAAAACGTTCTTCGGGGCGAAAACTCTCAAGGATCTTACCGCTGTTGAGATCCAGTTCGATGTAACCCACTCGTGCACCCAACTGATCTTCAGCATCTTTTACTTTCACCAGCGTTTCTGGGTGAGCAAAAACAGGAAGGCAAAATGCCGCAAAAAAGGGAATAAGGGCGACACGGAAATGTTGAATACTCATACTCTTCCTTTTTCAATATTATTGAAGCATTTATCAGGGTTATTGTCTCATGAGCGGATACATATTTGAATGTATTTAGAAAAATAAACAAATAGGGGTTCCGCGCACATTTCCCCGAAAAGTGCCACCTGACGTC

**9. aPKC-ι Promoter RT-qPCR primers & amplicon sequence (Amplicon length: WT 141, MUT 143):**


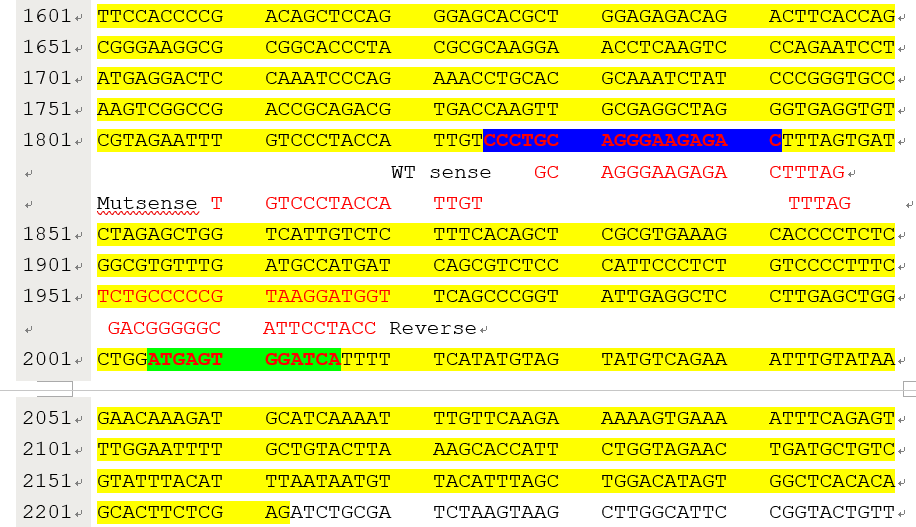

Supplement: Supplementary file 7 — Preliminary experiments, plasmids, viruses and gene sequences. [file 41389_2020_212_MOESM7_ESM.docx]
