# Supplementary material for: Transcription co-activator P300 activates Elk1-aPKC-ι signaling mediated epithelial-to-mesenchymal transition and malignancy in hepatocellular carcinoma
Source: Oncogenesis. 2020 Mar 6;9(3):32. doi: 10.1038/s41389-020-0212-5 (PMC7060348; doi:10.1038/s41389-020-0212-5)
Supplement: Supplementary file 9 — Cell Line Hep G2 STR Profile Report [file 41389_2020_212_MOESM9_ESM.pdf]

HepG2细胞STR鉴定说明书

1、细胞信息

- **细胞名称**：HepG2
- **细胞来源**：中科院上海细胞库
- **STR鉴定时间**：2017/10/13

2、STR鉴定

应用于生物医学研究领域的哺乳动物细胞存在被错误鉴定和交叉污染的问题，NIH 和 ATCC 近两年都对此发出呼吁，要求研究者对细胞进行鉴定。每个被分析的人源细胞系都有其独特的 DNA 重复模式，因此通过与基础图谱进行比对，即可对每一批新细胞系的种类进行确认。

本鉴定采用 ATCC 推荐的标准方法 STR 对所用细胞进行鉴定[1]。

- **鉴定流程图**

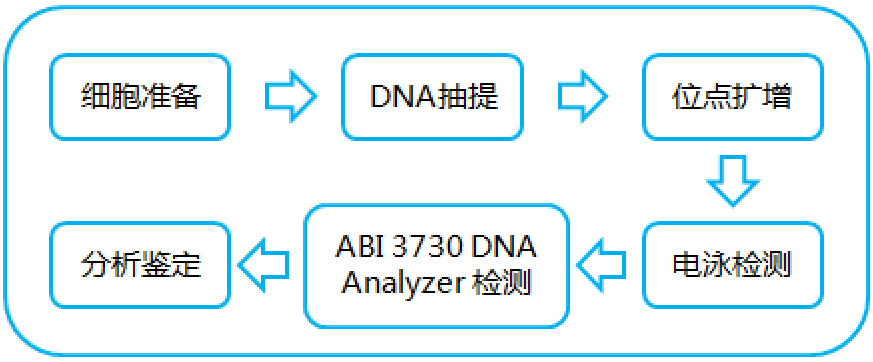

- **鉴定引物**
  本鉴定参考ATCC和JCRB数据库中的基因座，引物参考 http://www.cstl.nist.gov/div831/strbase/网站公布，可方便与权威的数据库进行比对，准确性极高。

具体引物序列：

位点名称	碱基排列顺序（5’ ...3’ ）	
Amelogenin	Forward primer:	ACCTCA TCC TgggCACCCTggTT
	Reverse primer:	AggCTTgAggCCAACCATCAg
CSF1PO	Forward primer:	AAC CTgAgTCTgCCAAggACTAgC
	Reverse primer:	TTC CACACACCACTggCCATCTTC
D13S317	Forward primer:	ACAgAAgTCTgggATgTggA
	Reverse primer:	gCCCAAAAAgACAgACAgAA
D16S539	Forward primer:	gATCCCAAgCTC TTC CTC TT
	Reverse primer:	ACgTTTgTgTgTgCATCTgT

D5S818	Forward primer:	gggTgATTTTCCTCTTTggT
	Reverse primer:	TgA TTC CAA TCATAgCCA CA
TPOX	Forward primer:	ACTggCACAgAACAggCACTTAgg
	Reverse primer:	ggAggAACTgggAACCACACAggT
D7S820	Forward primer:	TgTCATAgTTTAgAACgA ACTAAC g
	Reverse primer:	CTgAggTATCAAAAACTCAgAgg
D21S11	Forward primer: ATA TGT GAG TCA ATT CCC CAA G<br>Reverse primerTGT ATT AGT CAA TGT TCT CCA G	
TH01	Forward primer:	ATTCAA AgggTATCTgggCTCTgg
	Reverse primer:	gTgggCTgA AAA gCTCCCgATTAT
vWA	Forward primer:	gCC CTA gTggATgATAAgAATAATCAgTATgTg
	Reverse primer:	ggACAgATgATA AATACA TAggATggATgg

3、STR鉴定结果及说明

- **STR鉴定结果**

编号	位点	理论分型	理论大小	实际大小	实际分型	是否相符
1	Amelogenin	X,Y	212,218	211.86,217.44	X,Y	是
2	CSF1PO	10,11	307,311	306.52,310.77	10,11	是
3	D13S317	9,13	180,196	177.9,193.94	9,13	是
4	D16S539	12.13	161,165	159.07	12	是
5	D5S818	11,12	149,153	147.55,151.92	11,12	是
6	TPOX	8,9	232,236	230.32,234.19	8,9	是
7	D7S820	10	214	212.37	10	是
8	D21S11	29,31	222,230	223.44/231.32	29,31	是
9	TH01	9	195	195.19	9	是
10	vWA	17	151	151.24	17	是

（各细胞株的STR位点和Amelogenin位点的分型图谱见附图。）

- **鉴定说明**

1、STR分型鉴定结果参考ATCC数据库，国家实验细胞资源共享平台或者已有其他公司鉴定细胞的分型结果。

2、检测的细胞株与ATCC数据库共享平台公布的标准分型的匹配度≥80%，则认为此细胞株与标准细胞系相关。

3、每个位点的不同分型理论大小由http://www.cstl.nist.gov/div831/strbase/网站公布(详见附件)。

4、鉴定结论

该细胞10个基因座均未出现三等位基因现象，说明未与其他细胞交叉污染；该株细胞DNA分型与ATCC细胞库(国家实验细胞资源共享平台细胞库)中细胞分型匹配度≥80%，细胞应为HepG2细胞。

【参考文献】：

1. Shahram Azaria,Nahid Ahmadia, Mahmood Jeddi Tehrania,Fazel Shokria. Profiling andauthenticationofhumancelllinesusingshorttandemrepeat(STR)loci:Reportfromthe NationalCellBankofIran. Biologicals,Volume 35, Issue 3,June2007,Pages 195–202.

原始数据：

按照检测仪器（ABI 3730 DNA Analyzer）的标准，信号值大于1000可信，小于200的样品是不可信的，200-1000之间的根据经验，有的峰值是可信的

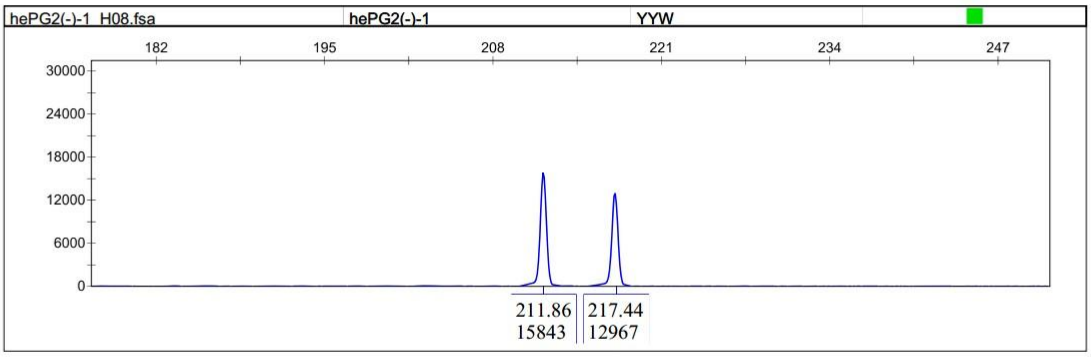

Amelogenin

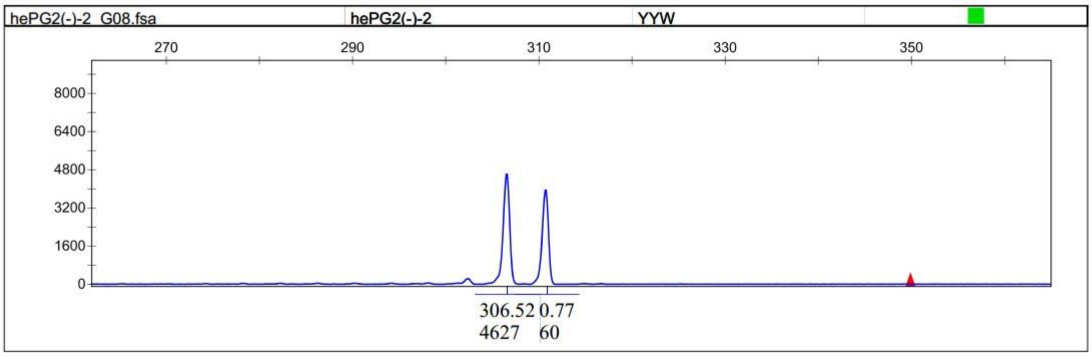

CSF1PO

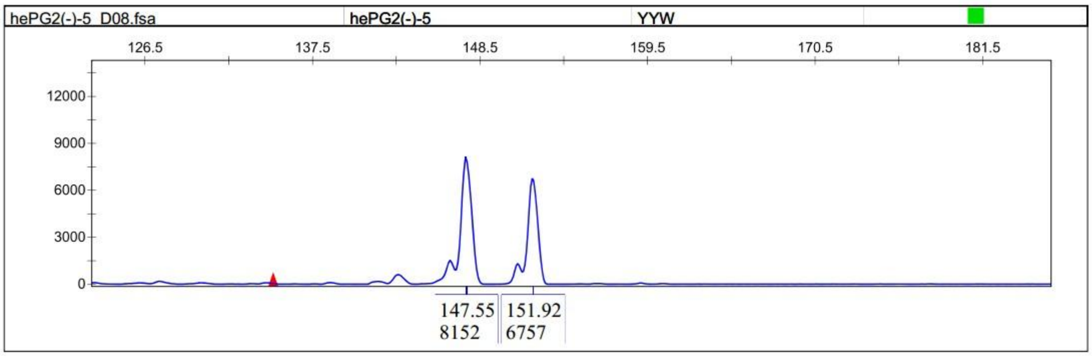

D5S818

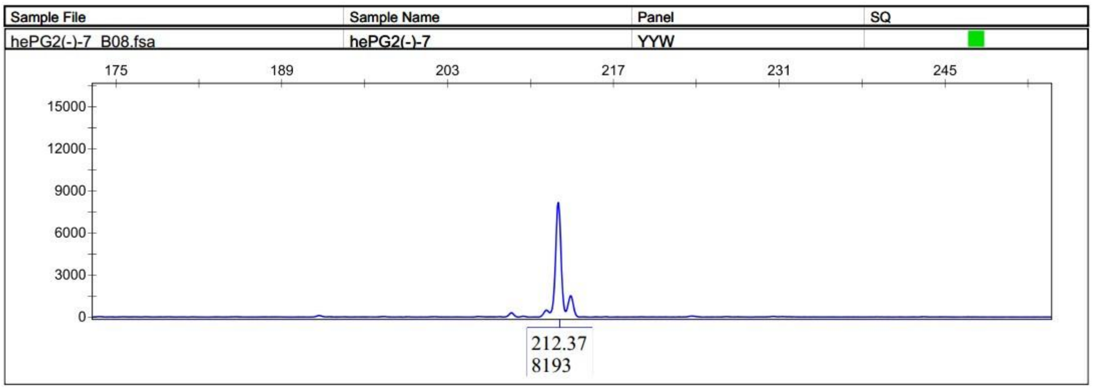

D7S820

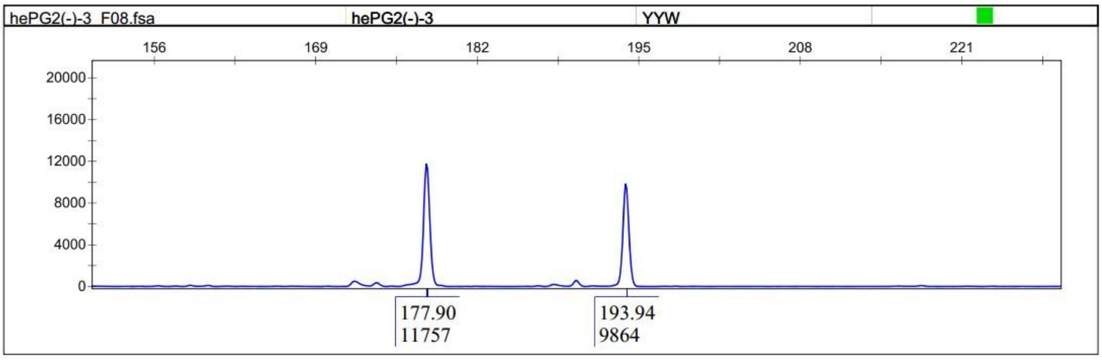

D13S317

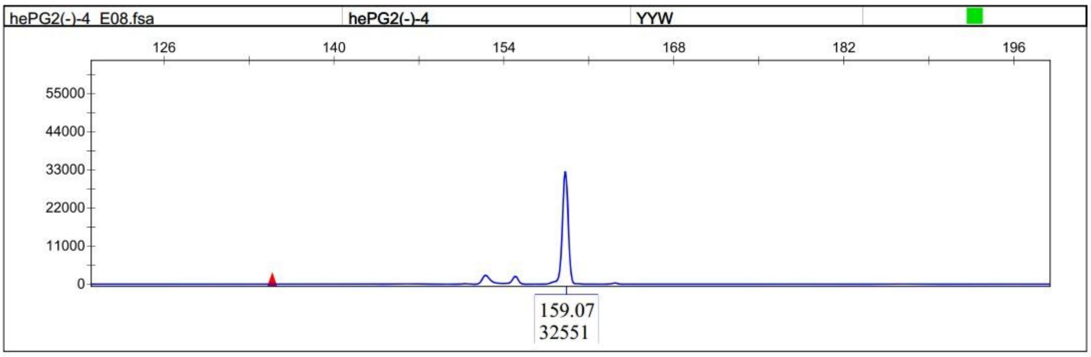

D16S539

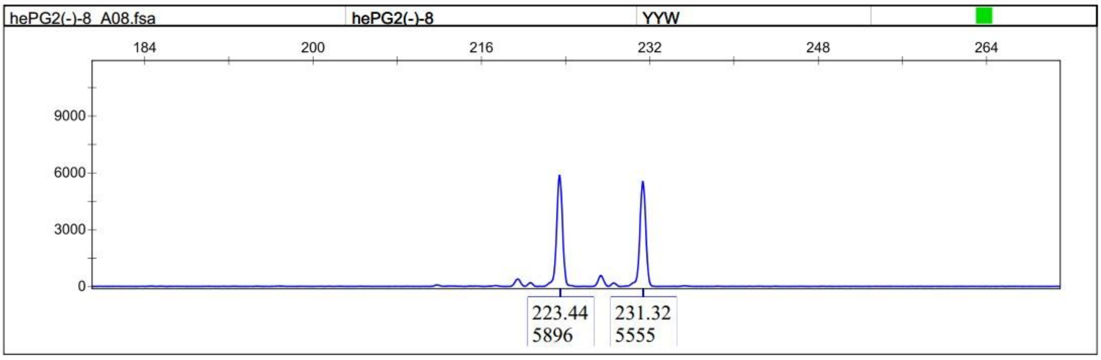

D21S11

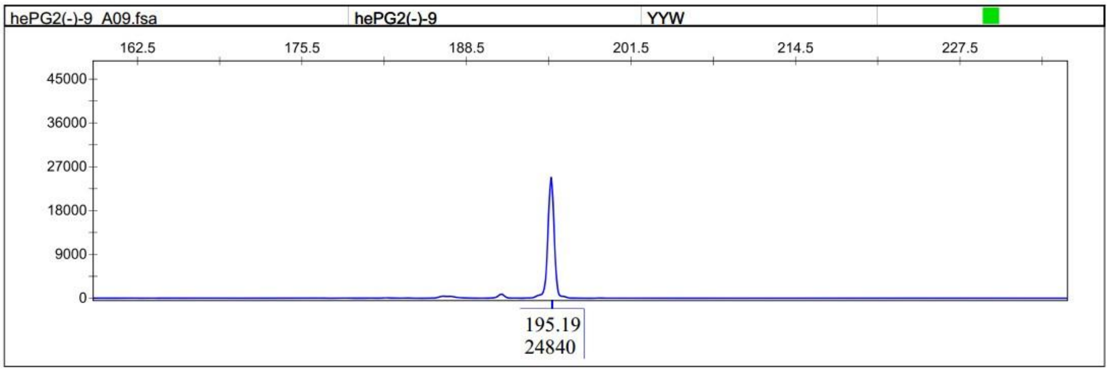

TH01

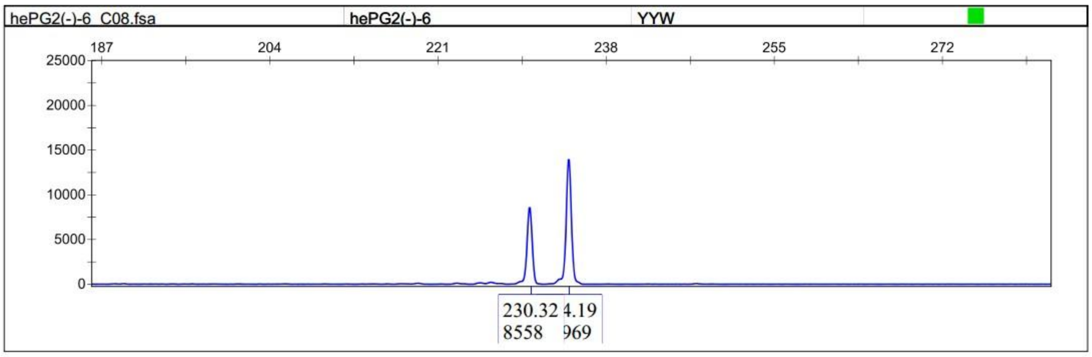

TPOX

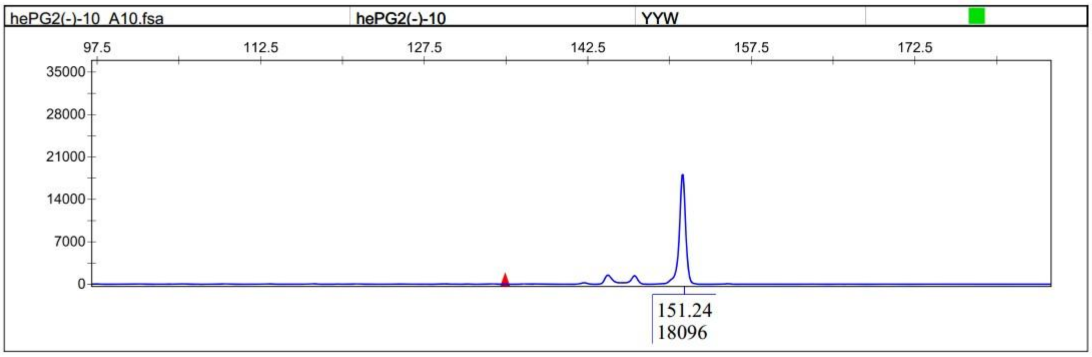

vWA

上海吉凯基因化学技术有限公司　Shanghai GenechemCo.,Ltd.
地址：上海张江高科技园区爱迪生路 332 号　电话：(86)21 51320189　传真：(86)21 51370635
Add：NO.332, Aidisheng Road, Zhangjiang Hi-Tech Park, Shanghai 201203 P.R.China　Tel : (86)21 51320189　Fax : (86)21 51370635
